# Genetic basis of unstable expression of high gamma-tocopherol content in sunflower seeds

**DOI:** 10.1186/1471-2229-12-71

**Published:** 2012-05-18

**Authors:** María J García-Moreno, José M Fernández-Martínez, Leonardo Velasco, Begoña Pérez-Vich

**Affiliations:** 1 Instituto de Agricultura Sostenible (IAS-CSIC), Alameda del Obispo s/n, 14004, Córdoba, Spain

## Abstract

**Background:**

Tocopherols are natural antioxidants with both *in vivo* (vitamin E) and *in vitro* activity. Sunflower seeds contain predominantly alpha-tocopherol (>90% of total tocopherols), with maximum vitamin E effect but lower *in vitro* antioxidant action than other tocopherol forms such as gamma-tocopherol. Sunflower germplasm with stable high levels of gamma-tocopherol (>85%) has been developed. The trait is controlled by recessive alleles at a single locus *Tph2* underlying a gamma-tocopherol methyltransferase (gamma-TMT). Additionally, unstable expression of increased gamma-tocopherol content in the range from 5 to 85% has been reported. The objective of this research was to determine the genetic basis of unstable expression of high gamma-tocopherol content in sunflower seeds.

**Results:**

Male sterile plants of nuclear male sterile line nmsT2100, with stable high gamma-tocopherol content, were crossed with plants of line IAST-1, with stable high gamma-tocopherol content but derived from a population that exhibited unstable expression of the trait. F_2_ seeds showed continuous segregation for gamma-tocopherol content from 1.0 to 99.7%. Gamma-tocopherol content in F_2_ plants (average of 24 individual F_3_ seeds) segregated from 59.4 to 99.4%. A genetic linkage map comprising 17 linkage groups (LGs) was constructed from this population using 109 SSR and 20 INDEL marker loci, including INDEL markers for tocopherol biosynthesis genes. QTL analysis revealed a major QTL on LG 8 that corresponded to the gamma-TMT *Tph2* locus, which suggested that high gamma-tocopherol lines nmsT2100 and IAST-1 possess different alleles at this locus. Modifying genes were identified at LGs 1, 9, 14 and 16, corresponding in most cases with gamma-TMT duplicated loci.

**Conclusions:**

Unstable expression of high gamma-tocopherol content is produced by the effect of modifying genes on *tph2*^*a*^ allele at the gamma-TMT *Tph2* gene. This allele is present in line IAST-1 and is different to allele *tph2* present in line nmsT2100, which is not affected by modifying genes. No sequence differences at the gamma-TMT gene were found associated to allelic unstability. Our results suggested that modifying genes are mostly epistatically interacting gamma-TMT duplicated loci.

## Background

Tocopherols are the main antioxidants present in seed oils. They form a family of four fat-soluble compounds with vitamin E activity named alpha-, beta-, gamma-, and delta tocopherol. Tocopherols contain a 6-chromanol ring structure methylated to varying degrees at positions 5, 7, and 8, and an isoprenoid-derived C16 saturated side chain at position 2. The four tocopherols differ by the number and positions of the methyl groups on the 6-chromanol ring. Alpha-tocopherol is trimethylated, beta- and gamma-tocopherol are dimethylated, and delta-tocopherol is monomethylated [1].

Tocopherols are important antioxidants operating both *in vivo*, protecting cells from oxidative damage, as well as *in vitro*, protecting oils and oil-based products from oxidation [2]. The relative *in vivo* and *in vitro* antioxidant properties of the specific tocopherols is determined by their chemical structure. The relative biological activity of the tocopherols is estimated as 100% for alpha-tocopherol, 15 to 27% for beta-tocopherol, 3 to 20% for gamma-tocopherol, and 0.3 to 2% for delta-tocopherol [3]. However, there is a widespread confusion concerning their relative potency *in vitro*[4], though it is generally accepted that alpha-tocopherol shows better antioxidant activity in fats and oils at lower concentrations, but at higher concentrations gamma-tocopherol is a more active antioxidant [5]. Studies conducted in sunflower seed oil, in which alpha-tocopherol accounts for more than >90% of the total tocopherols, concluded that substitution of alpha-tocopherol by gamma-tocopherol has a positive impact on the stability of the oil [6-9].

Four sunflower germplasms named LG-17, T2100, IAST-1, and IAST-540 in which alpha-tocopherol in the seeds was almost completely replaced by gamma-tocopherol have been developed [6,10,11]. Gamma-tocopherol in the novel germplasm accounts for more than 85% of the total seed tocopherols, compared with more than 90% alpha-tocopherol in conventional sunflower seeds. The increased gamma-tocopherol levels are produced by recessive alleles at the *Tph2* locus [6,12,13], which encodes a gamma-tocopherol methyltransferase (gamma-TMT) enzyme [14]. This enzyme catalyzes the methylation of delta- and gamma-tocopherol to yield beta- and alpha-tocopherol, respectively [15]. Gamma-TMT mutation in sunflower disrupts the synthesis of alpha-tocopherol and causes the accumulation of gamma-tocopherol [14]. In a detailed sequence analysis of the gamma-TMT gene in sunflower, Hass et al. [14] identified two gamma-TMT paralogs (*gamma-TMT-1* and *gamma-TMT-2*) and five different haplotypes (haplotypes 4 and 5 corresponding to paralogs 1 and 2, respectively). Both gamma-TMT paralogs 1 and 2 cosegregated with *Tph2* and were mapped to linkage group (LG) 8 of the sunflower linkage map. Even though the *Tph2* mutation reduced or disrupted the expression of the two paralogs in developing sunflower seeds, none of the DNA polymorphisms found within the gamma-TMT *Tph2* gene were associated with the high gamma-tocopherol phenotype [14]. The authors suggested that the *Tph2* mutation must be very tightly linked to the gamma-TMT locus on LG 8 and may disrupt regulatory sequences.

Phenotypic studies of Demurin et al. [16] and García-Moreno et al. [13] concluded that the four high gamma-tocopherol lines LG-17, T2100, IAST-1, and IAST-540 possess the same allele at *Tph2*, as no transgressive segregations were observed in crosses involving the four lines. Lines LG-17, T2100 and IAST-540 were isolated from germplasm or M_1_ mutant plants that segregated for high alpha-tocopherol (>90%) and high gamma-tocopherol (>85%), with no intermediate levels of both tocopherol forms being observed [6,10-12]. Conversely, IAST-1 was isolated from an M_2_ plant that exhibited large variation for alpha- and gamma-tocopherol levels, with gamma-tocopherol content in M_3_ seeds showing a continuous variation from zero to 84.6% and M_4_ seeds from selected M_3_ plants showing a variation from 60.4 to 97.4% gamma-tocopherol, which was uniformly high (>90%) in M_5_ seeds from selected M_4_ plants [11]. García-Moreno et al. [17] suggested that the intermediate gamma-tocopherol levels observed during the isolation of IAST-1 might be produced by the presence of modifying genes that determined unstable expression of mutated alleles at *Tph2* locus. Modifying genes have been found to influence important traits in sunflower such as high oleic acid content [18] or broomrape resistance [19].

In this study, the genetic analysis of a population that showed segregation from low to high gamma-tocopherol values obtained from the cross between the two high gamma-tocopherol lines IAST-1 and nmsT2100 has been carried out. In the course of this analysis, we identified two different alleles at the gamma-TMT *Tph2* locus at LG 8. The allele *tph2* was present in line nmsT2100, whereas the allele *tph2*^*a*^ was identified in line IAST-1. Additionally, we found four modifying genes at LGs 1, 9, 14 and 16 that in most cases corresponded to duplicated gamma-TMT loci. Modifying genes influenced the expression of *tph2*^*a*^ alleles, but did not affect *tph2* alleles.

## Results

### Phenotypic segregations

**Figure 1:**
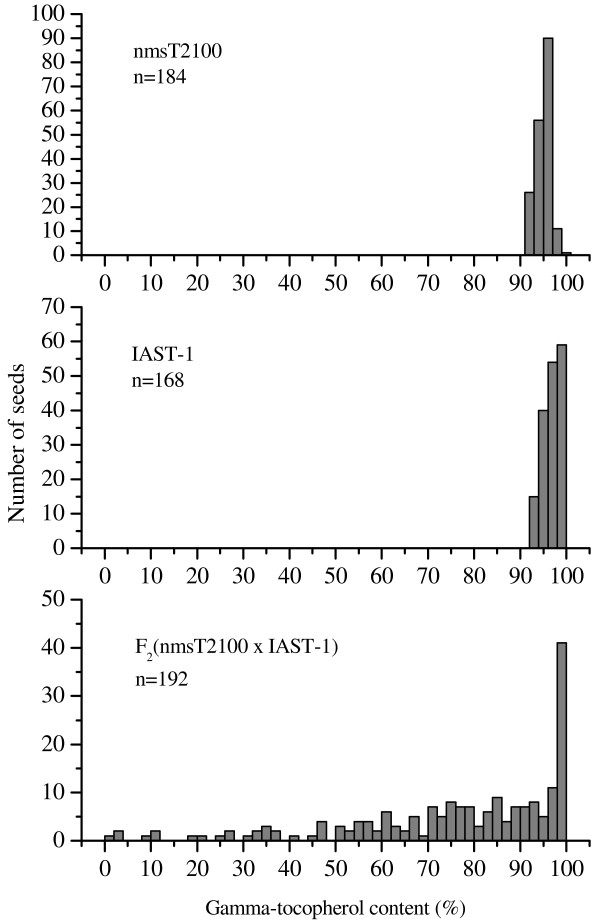
**Phenotypic segregation of the F**_**2**_** generation.** Histograms of gamma-tocopherol content (% of total tocopherols) in sunflower lines nmsT2100, IAST-1, and the F_2_ generation from their crosss.

**Figure 2:**
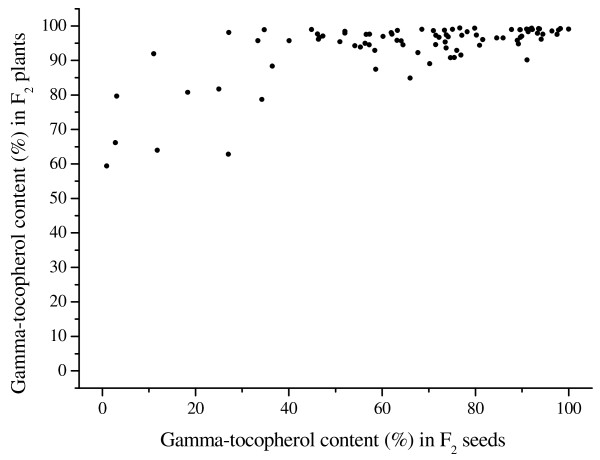
**Phenotypic segregation of the F**_**2**_**vs F**_**3**_** generations.** Gamma-tocopherol content (% of total tocopherols) in F_2_ seeds from the cross nmsT2100 x IAST-1 and their corresponding F_2_ plants (24 F_3_ seeds averaged per F_2_ plant).

Seeds of sunflower lines nmsT2100 (fertile plants) and IAST-1 showed uniformly high gamma-tocopherol content, from 91.2 to 99.8% in nmsT2100 and from 92.3 to 99.4% in IAST-1. F_1_ seeds from the cross between nmsT2100 and IAST-1 had also high gamma-tocopherol content, from 92.6 to 97.2%. However, large segregation for gamma-tocopherol content was observed in F_2_ seeds from some F_1_ plants. Particularly, the analysis of 192 F_2_ seeds from an F_1_ plant derived from an F_1_ seed with 95.5% gamma-tocopherol revealed a continuous large segregation for the trait, from 1.0 to 99.7%, with no discrete phenotypic classes being observed and a non-normal distribution (p < 0.0001; Kolmogorov-Smirnov test) (Figure 1). Variation for gamma-tocopherol content was smaller at the F_2_ plant generation (average of 24 individual F_3_ seeds), which segregated from 59.4 to 99.4% (Figure 2). However, examination of variation of individual F_3_ seeds within each F_2_ plant showed that minimum gamma-tocopherol content in individual F_3_ seeds from the different F_3_ families ranged from 0.0 to 98.4%, whereas maximum gamma-tocopherol content was in all cases above 94%. No discrete classes could be distinguished for minimum gamma-tocopherol content in the F_2:3_ population (data not shown).

### Map construction and candidate gene mapping

One hundred and sixty three out of 437 (37.3%) simple sequence repeat (SSR) and insertion-deletion (INDEL) markers were polymorphic in the screening of the high gamma-tocopherol lines nmsT2100 and IAST-1 and four randomly selected F_2_ individuals from their cross. A set of 128 high quality, evenly spaced, and preferably co-dominant markers were genotyped in the F_2_ population. Final linkage map for QTL analyses comprised 129 marker loci (109 SSR and 20 INDEL) grouped on 17 linkage groups (Table 1). The total genetic distance covered by these marker loci was 792.4 cM, with an average marker interval of 7.0 cM (Table 1). Linkage groups contained between 3 and 16 marker loci, with 99.6% of the mapped genome being within 20 cM to the nearest marker. No marker loci had significantly distorted segregation ratios (P < 0.001).

**Table 1:** nmsT2100 x IAST-1 linkage map coverage

**LG coverage**^**2**^					
**Coverage (cM)**					
**LG**^**1**^	**SSR**	**INDEL**	**Mean**	**Largest interval**	**Total**
1	13	3	4.3	19.3	64.0
2	6	0	4.4	8.2	22.1
3	6	1	16.6	27.8	99.7
4	5	1	4.2	8.1	20.9
5	5	1	5.5	15.5	27.6
6	5	2	3.6	13.4	21.5
7	7	1	10.7	43.4	74.9
8	8	3	4.5	14.9	44.6
9	6	0	6.6	22.8	32.9
10	7	4	10.1	29.8	100.9
11	3	0	6.6	8.9	13.2
12	4	2	4.3	7.5	21.3
13	7	0	8.3	31.2	50.0
14	7	1	4.6	10.2	32.4
15	6	0	10.1	36.5	50.5
16	8	1	9.6	32.1	76.8
17	6		7.8	17.1	39.1
Total	109	20			792.4

^1^LG = Linkage group.

^2^SSR = Number of SSR loci; INDEL = Number of INDEL loci.

Genome coverage offered by the marker set used for QTL analysis in the nmsT2100 x IAST-1 population. Distances are expressed in Haldane cM.

**Figure 3:**
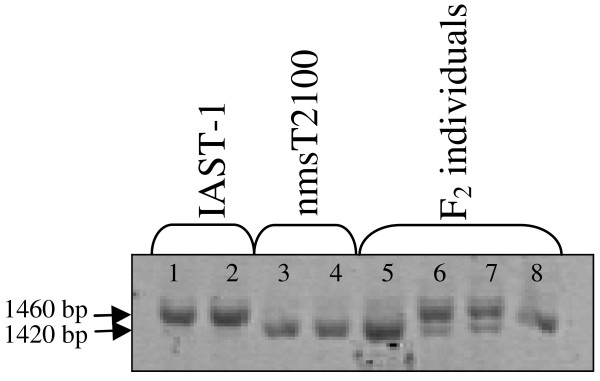
**Amplification profile of gamma-TMT_F1/F2/R24 INDEL marker.** Amplification profile of gamma-TMT_F1/F2/R24 INDEL marker in replicate samples of the high gamma-tocopherol parent line IAST-1, the high gamma-tocopherol parent line nmsT2100 and four F_2_ individuals. Lane 1–2, replicate samples of IAST-1; lanes 3–4, replicate samples of nmsT2100 and lanes 5–8, F_2_ individuals.

**Figure 4:**
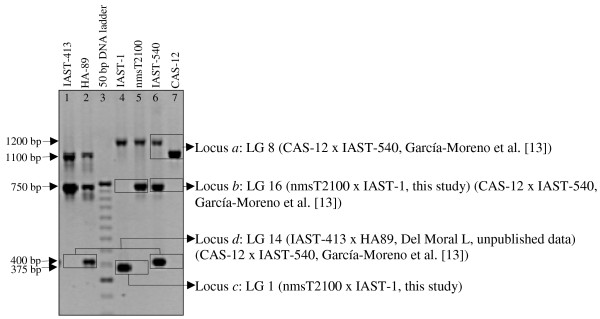
**Amplification profile of gamma-TMT_F9/R24 INDEL marker.** Amplification profile of gamma-TMT_F9/R24 INDEL marker in single samples of the high gamma-tocopherol IAST-1, nmsT2100, IAST-540 lines and the standard gamma-tocopherol IAST-413, HA-89 and CAS-12 lines. Lane 1, IAST-413; lane 2, HA-89; lane 3, 50 bp DNA ladder; lane 4, IAST-1; lane 5, nmsT2100; lane 6, IAST-540 and lane 7, CAS-12. Polymorphic loci are highlighted by boxes, and the linkage group and the population in which they have been mapped are shown at the right of the figure.

INDEL markers for the gamma-TMT gene described by Hass et al. [14] were not polymorphic between nmsT2100 and IAST-1 or showed polymorphism in secondary loci. Therefore, new primer combinations based on the full-lenght sequence of the gamma-TMT gene in nmsT2100 and IAST-1 (see below) were designed and tested. A primer combination (gamma-TMT-F1/F2/R24) based on the forward primers F1 and F2 placed upstream and dowstream, respectively, of the T39 transcription initiation site [14], and a reverse primer R24 complementary to the DNA sequence in exon 2 amplified a primary gamma-TMT locus that was polymorphic between IAST-1 and nmsT2100 (Figure 3). The polymorphic band was about 1460 bp in IAST-1 and 1420 bp in nmsT2100 (Figure 3). When screened against the individuals from the F_2_ population, the gamma-TMT-F1/F2/R24 locus was co-dominantly mapped to LG 8 at the previously described position of the gamma-TMT *Tph2* gene [14]. Other INDEL markers for the gamma-TMT gene were also tested. The F9/R24 gamma-TMT INDEL marker showed three loci in the nmsT2100 and IAST-1 parental lines (Figure 4). A primary locus (gamma-TMT-F9/R24a) corresponding to a band of about 1200 bp was not polymorphic with this primer combination (Figure 4). This locus, however, was demostrated to co-segregate with *Tph2* and was co-dominantly mapped to LG 8 in the population CAS-12 x IAST-540, also segregating for gamma-tocopherol content [13]. A second locus (gamma-TMT-F9/R24b) that showed a dominant polymorphism, with a band of about 750 bp present in nmsT2100 and absent in IAST-1, was mapped to LG 16 (Figure 4). The locus was also mapped to LG 16 in the CAS-12 x IAST-540 population. Finally, a third locus (gamma-TMT-F9/R24c) that also showed a dominant polymorphism, with a band of 375 bp absent in nmsT2100 and present in IAST-1, was mapped to LG 1 (Figure 4). In addition to the gamma-TMT-F9/R24a, b and c loci, a fourth gamma-TMT locus was identified in populations other than nmsT2100 x IAST-1. This locus was named gamma-TMT-F9/R24d and was mapped to LG 14 in populations CAS-12 x IAST-540 [13] and IAST-413 x HA-89 (Del Moral L, unpublished data) (Figure 4). Finally, the locus *MT-2* of the 2-methyl-6-phytyl-1,4-benzoquinone/2-methyl-6-solanyl-1,4-benzoquinone methyltransferase (MPBQ/MSBQ-MT) gene was mapped to LG 4 using the F24/R25 INDEL marker [20]. None of the other markers for tocopherol biosynthesis genes tested were polymorphic between nmsT2100 and IAST-1.

### QTL analyses

One-factor analysis of variance revealed that the gamma-TMT-F1/F2/R24 locus on LG 8 was underlying a major QTL affecting gamma-tocopherol content. This locus had a significant effect on gamma-tocopherol content of the F_2_ and the F_3_ generations (Table 2) and corresponded to the gamma-TMT *Tph2* gene also mapped to the same position on LG 8 by Hass et al. [14]. The mean and the standard deviation for gamma-tocopherol content in the F_2_ seed generation of plants homozygous for the IAST-1 allele were 52.3 ± 29.6%, whereas those for plants homozygous for the nmsT2100 allele were 98.9 ± 0.8% (Table 2). These results suggested the existence to two different alleles at the gamma-TMT *Tph2* locus in lines nmsT2100 and IAST-1. The allele at IAST-1 was unstable, producing in homozygous condition a broad distribution ranging from 0.97 to 89.02% in the F_2_ seed generation, whereas the allele at nmsT2100 was highly stable and resulted in gamma-tocopherol values above 97%. Similar results were observed in the F_3_ seed generation (Table 2). It is interesting to note that only three F_2_ plants homozygous for the nmsT2100 allele produced sufficient number of F_3_ seeds for tocopherol analyses, compared to 26 F_2_ plants homozygous for the IAST-1 allele (Table 2). This was probably caused by a close linkage between *Tph2* and *Ms11* loci at LG 8, the latter responsible for male sterility in the nuclear male sterile (NMS) line P21 [21] from which *Ms11* was introgressed to nmsT2100.

**Table 2:** Effect of gamma-TMT-F1/F2/R24 on seed gamma-tocopherol content

**Seed generation**	**No. individuals within each marker class**			**Mean ± SD for gamma-tocopherol content (% total tocopherols)within each marker class**			**ANOVA analysis**	
	A	H	B	A(IAST-1)	H	B(nmsT2100)	*F*	*P*
F_2_	32	73	27	52.3a ± 29.6	72.2b ± 19.1	98.9c ± 0.8	38.6	<0.001
F_3_	26	58	3	86.8a ± 11.8	96.9b ± 2.6	98.6b ± 0.9	20.4	<0.001

Mean values within rows followed by the same letter are not significantly different at 0.05 level of probability (Duncan’s multiple range test).

Association between the gamma-TMT-F1/F2/R24 locus on LG 8 and gamma-tocopherol content (% of the total tocopherols) determined by variance analysis in the nmsT2100 x IAST-1 population. Mean gamma-tocopherol ± standard deviation (SD) are presented in different genotypic classes: A = homozygous with respect to the allele derived from IAST-1, B = homozygous with respect to the allele derived from nmsT2100, H = heterozygous.

**Figure 5:**
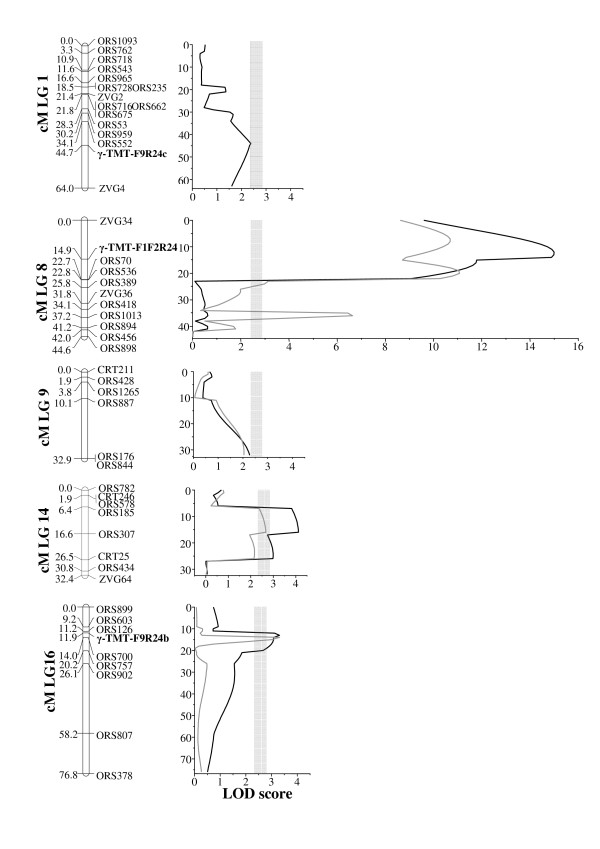
**LODs for gamma-tocopherol QTL.** Likelihood odds (LODs) for F_2_ (black line) and F_3_ (grey line) gamma-tocopherol QTL on linkage groups (LGs) 1, 8, 9, 14 and 16 in nmsT2100 x IAST-1. Gamma-tocopherol-TMT (γ-TMT) loci are highlighted in bold. The thresholds of the LOD score are indicated by *grey boxes* corresponding to their 0.95 confidence intervals (from 2.48 to 2.76) for gamma-tocopherol content in the F_2_ and the F_3_ generations.

Composite interval mapping analyses confirmed the existence of a QTL with a main effect centered on the gamma-TMT-F1/F2/R24 locus on LG 8 (Table 3 and Figure 5). This QTL was named *Tph2-gamma-TMT* and explained 41.2% and 44.4% of the F_2_ and F_3_ gamma-tocopherol phenotypic variance, respectively (Table 3). A second QTL peak on LG 8 16 cM apart from *Tph2-Gamma-TMT* was identified only in the QTL analysis of the F_3_ data (Figure 5). This adjacent peak might be a ghost QTL resulting from the distorted F_3_ data in this region and was not taken into consideration to protect against type I errors in declaring QTLs.

**Table 3:** QTL affecting gamma-tocopherol content in the nmsT2100 x IAST-1 population

								**Significant gene effects**^**5**^	
**Generation**	**QTL**	**LG**^**1**^	**Pos.**^**2**^	**Supp int.**^**3**^	**Marker interval**^**4**^	**LOD**	***R***^**2**^**(%)**	***a***	***d***
F_2_	*GamT1.1*	1	44	29-63	ORS552 to Gamma_TMT_F9/R24c	2.4	7.8		−2.96*
	*Tph2_Gamma-TMT*	8	12	7-15	ZVG34 to Gamma_TMT_F1/F2/R24	15.0	41.2	20.9**	
	*GamT9.1*	9	32	19-32	ORS887 to ORS176	2.3^6^	7.5	−7.7**	
	*GamT14.1*	14	16	6-17	ORS185 to ORS307	4.1	13.1	−11.5**	
	*GamT16.1*	16	13	11-21	Gamma_TMT_F9/R24b to ORS700	3.4	10.9		
					**Total**	17.2	44.4		
					addTph2_Gamma-TMT*add*GamT14.1*			9.9*	
					addTph2_Gamma-TMT*add*GamT16.1*			14.0**	
					**Total epistasis**	21.7	52.4		
								**Significant gene effects**^**5**^	
**Generation**	**QTL**	**LG**^**1**^	**Pos.**^**2**^	**Supp int.**^**3**^	**Marker interval**^**4**^	**LOD**	***R***^**2**^**(%)**	***a***	***d***
F_3_	*Tph2_Gamma-TMT*	8	20	16-23	Gamma_TMT_F1/F2/R24 to ORS70	11.1	44.0	10.9**	
	*GamT9.1*	9	31	13-32	ORS887 to ORS176	2.04^6^	10.1	−2.6**	
	*GamT14.1*	14	16	6-27	ORS185 to ORS307	2.7	13.0	−3.5**	2.5*
	*GamT16.1*	16	14	13-16	ORS700 to ORS757	3.3	15.9	10.6**	−22.9**
					**Total**	18.1	61.2		
					addTph2_Gamma-TMT*add*GamT9.1*			6.8**	
					addTph2_Gamma-TMT*add*GamT14.1*			6.7**	
					addTph2_Gamma-TMT*add*GamT16.1*			8.5**	
					**Total epistasis**	23.8	71.3		

** = significant at the 0.01 and * = significant at the 0.05 probability level. ^1^LG = Linkage group. ^2^Absolute position from the top of the LG in centiMorgans (cM). ^3^One-LOD support interval in centiMorgans: Refers to the region flanking each QTL peak in which LOD scores decline by one. ^4^Markers flanking the likelihood peak for a putative QTL. ^5 ^a = additive effect. A positive sign means an increase of the trait value due to nmsT2100 alleles. d = dominant effect. a and d estimates, as well as total R2 and LOD score values were obtained from a simultaneous fit of all putative QTL using multiple regression. ^6^QTL detected below the LOD threshold.

Besides *Tph2-Gamma-TMT*, four other QTL with a moderate effect on gamma-tocopherol content were observed at the F_2_ level. These QTL were located at LG 1 (*GamT1.1*), 9 (*GamT9.1)*, 14 (*GamT14.1*), and 16 (*GamT16.1*). They individually accounted for 2.3-4.1% of trait variation (Table 3 and Figure 5). The QTL on LG 9, 14 and 16 were also detected in the F_3_ analyses at practically identical positions (Table 3 and Figure 5). The model with all the QTL explained 44.4% and 61.2% of the phenotypic variance for gamma-tocopherol content in the F_2_ and the F_3_ generations, respectively. The QTL peaks of *GamT1.1* and *GamT16.1* were centered on the gamma-TMT loci mapped at these linkage groups (gamma-TMT-F9/R24c in LG1 and gamma-TMT-F9/R24b in LG 16) (Figure 5). Additionally, the QTL peak of *GamT14.1* was likely to correspond to the gamma-TMT-F9/R24d locus mapped to LG 14 in populations CAS-12 x IAST-540 [13] and IAST-413 x HA-89 (Del Moral L, unpublished data), located 1.5 cM from the ZVG64 marker locus at this LG.

Analysis for epistasis in the F_2_ generation through two-way ANOVA revealed significant interactions for gamma-tocopherol content involving the *Tph2-gamma-TMT* marker locus on LG 8 and markers on LG 1, LG 14 and LG 16. The most significant interactions involved the ORS185 marker locus on LG 14 (*F* = 10.5, *P* < 0.0001), the gamma-TMT_F9R24b locus on LG 16 (*F* = 4.22, *P* = 0.017), and the gamma-TMT_F9R24c locus on LG 1 (*F* = 3.12, *P* = 0.048), which were associated to the *GamT14.1*, *GamT16.1*, and *GamT1.1* QTL, respectively. Significant interactions involving the *Tph2-Gamma-TMT* QTL on LG 8, the *gamT14.1* on LG 14, and the *gamT16.1* QTL on LG 16 were also detected in the composite interval mapping analyses, with the percentage of explained phenotypic variance increasing from 44.4% to 52.4% in the F_2_ and from 61.2% to 71.3% in the F_3_ when epistatic interactions were included in the multiple-locus model (Table 3). Genotypic means for F_2_ gamma-tocopherol content in allelic combinations of the epistatically interacting marker loci were calculated to detail these interactions. Since ORS185 and gamma-TMT_F9R24b marker loci were dominant, genotypic means were computed using close co-dominant markers, ORS578 on LG 14 and ORS126 on LG 16 (Table 4). The results showed that QTL regions on LG 14 and LG 16 only had a significant phenotypic effect when the IAST-1 allele was present at the *Tph2-gamma-TMT* locus on LG 8 (gamma-TMT-F1/F2/R24). Thus, no significant differences were observed between different genotypes at ORS578 on LG 14 or ORS126 on LG 16 in presence of the nmsT2100 allele at *Tph2*. However, when the *Tph2* locus was homozygous for the IAST-1 allele, the presence of nmsT2100 alleles at ORS578 or ORS126 resulted in a drastic reduction of gamma-tocopherol content as compared to the presence of IAST-1 alleles (Table 4).

**Table 4:** Effect of epistatically interacting marker loci on seed gamma-tocopherol content

		**F**_**2**_**gamma-T**^**1**^				**F**_**2**_**gamma-T**^**1**^		
**Genotype ofγ-TMT-F1/F2/R24(LG 8)**	**Genotype of ORS578(LG 14)**	**Mean**	**SD**	**n**	**Genotype of ORS126(LG 16)**	**Mean**	**SD**	**n**
A (IAST-1)	B (nmsT2100)	23.48a	24.10	9	B (nmsT2100)	51.68ab	31.89	14
	H	64.60b	20.27	12	H	44.55a	27.08	14
	A (IAST-1)	67.48b	22.69	10	A (IAST-1)	81.26 cd	6.96	4
H	B (nmsT2100)	71.61b	18.93	18	B (nmsT2100)	77.84c	16.68	22
	H	71.00b	19.19	42	H	69.17bc	18.71	36
	A (IAST-1)	76.91b	19.60	13	A (IAST-1)	71.23c	22.38	15
B (nmsT2100)	B (nmsT2100)	98.92c	0.53	9	B (nmsT2100)	98.59d	0.97	7
	H	98.55c	0.87	9	H	98.96d	0.71	16
	A (IAST-1)	99.15c	0.86	9	A (IAST-1)	99.03d	0.82	4

Values within columns followed by the same letter are not significantly different at 0.05 level of probability (Duncan’s multiple range test).

^1^Mean and standard deviation (SD) for F_2_ gamma-T content and number of F_2_ individuals per genotypic class (n).

Genotypic means for F_2_ gamma-tocopherol (gamma-T) content (% of the total tocopherols) in allelic combinations at epistatically interacting marker loci. A = homozygous with respect to the allele derived from IAST-1, B = homozygous with respect to the allele derived from nmsT2100, H = heterozygous.

### Sequence analysis of gamma-TMT loci

Several loci amplified by the gamma-TMT F9/R24 INDEL marker in IAST-1 and nmsT2100 (Figure 4) were cloned and sequenced. A band of about 1200 bp from the non-polymorphic locus *a* and a band of about 375 bp from the dominant locus *c* that mapped to LG 1 were sequenced from IAST-1. A band of about 1200 bp from the non-polymorphic locus *a* and a band of about 750 bp from the dominant locus *b* that mapped to LG 16 were sequenced from nmsT2100. The locus *d* that mapped to LG 14 was sequenced from lines IAST-540 and HA-89.

The locus *a* fragment isolated from nmsT2100 harboured alleles from both gamma-TMT paralogs, which showed showed 100% sequence identity to gamma-TMT haplotype 4 (paralog 1) and 5 (paralog 2) from Hass et al. [14]. For IAST-1, the locus *a* sequence showed 100% sequence identity to gamma-TMT haplotype 4. The locus *b* consensus sequence was 785 bp long and showed a significant homology to gamma-TMT haplotypes 2 and 3 (GenBank accessions nos. DQ229829 and DQ229830, 3e^−58^), with 94% maximum sequence identity spanning 61% of the locus *b* fragment coverage. The consensus sequence for locus *c* (388 bp long) showed a significant homology to gamma-TMT haplotypes 4 and 5 (GenBank accessions nos. DQ229831 to DQ229834, 4e^-14^), with 86% maximum sequence identity spanning 19% of the locus *c* fragment coverage. The sequences of locus *d* from IAST-540 (405 bp) and HA-89 (406 bp) were very similar, with a 96% of sequence identity between them. The consensus sequence obtained from this alignment showed significant homology to gamma-TMT haplotypes 1, 3, 4 and 5 (GenBank accession nos. DQ229828, DQ229830, and DQ229831 and DQ229834; 8e^−18^), with 89% maximum sequence identity spanning 18% of the fragment coverage.

**Figure 6:**
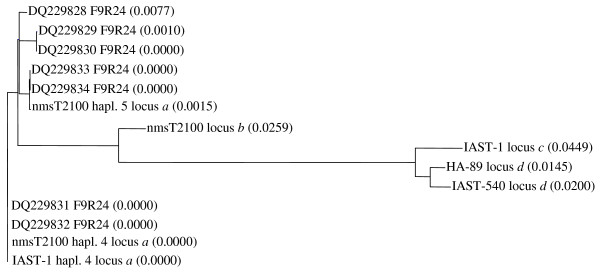
**Sequence alignment tree for gamma-TMT loci.** Sequence alignment tree obtained by aligning DNA genomic sequences from the different loci amplified with primer combination gamma-TMT F9/R24 and constructed using the AlignX program in the Vector NTI software suite. Calculated distance based on distances between all pairs of sequence values are shown in parenthesis following the molecule name. The locus *a* fragment isolated from nmsT2100 and IAST-1, the locus *b* isolated from nmsT2100, the locus *c* isolated from IAST-1, and the locus *d* isolated from HA-89 and IAST-540, together with the F9 to R24 region from gamma-TMT haplotypes 1 (DQ DQ229828), 2 (DQ DQ229829), 3 (DQ DQ229830), 4 (DQ DQ229831 and DQ DQ229832), and 5 (DQ DQ229833 and DQ DQ229834) are included.

Nucleotide sequences from IAST-1 locus *a*, nmsT2100 locus *a*, nmsT2100 locus *b*, IAST-1 locus *c*, HA-89 locus *d*, and IAST-540 locus *d* were aligned. The sequence alignment tree (Figure 6) revealed great similarity between loci *a* and *b* (85 to 87% of sequence identity) and between loci *c* and *d* (92–93% of sequence identity). Both groups were more distantly related, with 56 to 58% of sequence identity between locus *a* and the cluster of loci *c* and *d*.

### Full-length gamma-TMT genomic DNA sequences

Agarose gels from PCR products obtained with the F1 and R92 primers flanking the gamma-TMT gene mapped to LG 8 [14] revealed two bands in nmsT2100 and IAST-1 lines that were identified as the two gamma-TMT paralogs 1 and 2 described by Hass et al. [14]. Consensus nucleotide sequences for IAST-1 gamma-TMT paralog 1 (4126 bp) and paralog 2 (4280 bp) were identical to gamma-TMT haplotype 4 (GenBank accessions nos. DQ229831 and DQ229832) and 5 (GenBank accessions nos. DQ229833 to DQ229834), respectively, from Hass et al. [14] ( Additional file 1: Figure S1). Consensus nucleotide sequence for nmsT2100 gamma-TMT paralog 1 (4126 bp) was identical to gamma-TMT haplotype 4 [14] ( Additional file 1: Figure S1). Consensus nucleotide sequence for nmsT2100 gamma-TMT paralog 2 (4281 bp) was almost identical to gamma-TMT haplotype 5 [14], with the exception of five SNPs, one in the 5´UTR (G to A), one in intron 1 (C to T), and three in intron 4 (A to T) ( Additional file 1: Figure S1).

## Discussion

The results of this research suggest that high gamma-tocopherol lines IAST-1 and nmsT2100 possess different mutated alleles at the gamma-TMT *Tph2* locus on LG 8. The gamma-TMT catalyses the methylation step from gamma-tocopherol to alpha-tocopherol in sunflower seeds [14]. Mutated *tph2* alleles disrupt the activity of gamma-TMT, which results in accumulation of gamma-tocopherol [14]. The allele at nmsT2100 (*tph2*) is stable and not affected by modifying genes, whereas the allele at IAST-1 (*tph2*^*a*^) is unstable and affected by modifying genes. Putative modifying genes located at LG 1, 9, 14 and 16 were identified in the present research, being the effect of those on LG 1, 14 and 16 highly significant in the epistatic interaction with the *Tph2* locus on LG 8. Studies on expression of *Tph2* mutations in plant tissues other than seeds also pointed to differences between T2100 and IAST-1 lines, as the latter showed lower gamma-tocopherol content in leaves, roots, and pollen [22].

High gamma-tocopherol lines T2100 and IAST-1 were isolated following different strategies. T2100 derived from an open-pollinated cultivar that showed variation for high gamma-tocopherol content (>85%) at the single-seed level [9]. Genetic study of the trait in T2100 seeds indicated that the trait was controlled by recessive alleles at a single locus *Tph2* in such a way that *Tph2Tph2* and *Tph2tph2* genotypes produced low levels of gamma-tocopherol (<5%) and *tph2tph2* genotypes showed high levels of gamma-tocopherol (>85%), with no intermediate levels being observed [12]. On the contrary, IAST-1 derived from a mutagenesis program in which an M_2_ seed with intermediate gamma-tocopherol content (19.2%) was identified. The trait showed continuous variation for gamma-tocopherol levels (zero to 84.5%) in the M_3_ generation, which allowed selection of genotypes with stable high gamma-tocopherol content in the M_4_ generation [11]. A comparative genetic study between both lines concluded that they shared the same allele at *Tph2*, as no transgressive segregation was observed in the F_1_ and F_2_ generations from crosses between them [13]. However, the evaluation of several F_2_ populations from crosses of IAST-1 with T2100 and the conventional sunflower line HA89 showed that intermediate gamma-tocopherol values (5 to 85%) occurred in some F_2_ populations, whereas other F_2_ populations showed uniformly high gamma-tocopherol content (IAST-1 x T2100) or segregated into well-defined low and high gamma-tocopherol classes (IAST-1 x HA89) [17]. The results of this research suggest that intermediate gamma-tocopherol levels are produced by the effect of modifying genes on *tph2*^*a*^ alleles from IAST-1. We hypothesize that modifying genes were present in the mutagenized population from which IAST-1 was developed, where selection against negative alleles at modifying genes led to the isolation of IAST-1, and also in other lines such as HA89 and T2100. Modifying genes have no phenotypic effect in absence of alteration in the major gene [23]. In this research, it was also found that modifying genes have no phenotypic effect on the mutated allele at T2100 (*tph2*). Accordingly, they are expected to be segregating in HA89 and T2100 lines, which produced different segregation patterns in crosses with IAST-1 [17]. The occurrence of different segregation patterns depending on allelic configuration of modifying genes has been previously reported in sunflower for high oleic acid content [24,25] and broomrape resistance [19].

Full length genomic gamma-TMT sequences corresponding to the *Tph2* locus on LG 8 have been obtained in this study for both IAST-1 and nmsT2100. Both lines have two gamma-TMT paralogs. Paralog 1 is identical between the two lines and also to the gamma-TMT haplotype 4 from Hass et al. [14], which included sequences from both high and low gamma-tocopherol lines. Paralog 2 from IAST-1 is identical to gamma-TMT haplotype 5 from Hass et al. [14], which also included sequences from both high and low gamma-tocopherol lines, while paralog 2 from nmsT2100 carries slight SNP differences. Consequently, we have not found any sequence difference in the gamma-TMT gene from IAST-1 that would explain why the *tph2*^*a*^ allele present in this line is unstable and affected by modifying genes. Previous research did not identify sequence differences in the gamma-TMT gene related to the high gamma-tocopherol phenotype [14]. The authors found identical gamma-TMT paralog 1 and 2 sequences in both high and low gamma-tocopherol lines and reported that mutation leading to the high gamma-tocopherol trait in the sunflower material derived from the high gamma-tocopherol line LG-17 [6] may disrupt regulatory sequences of the gamma-TMT gene. Relating the nmsT2100 line, the slight sequence differences in gamma-TMT paralog 2 were changes to those nucleotides also present in paralog 1 sequences from IAST-1 and nmsT2100 (2 SNPs in the 5´UTR region and in intron 1) or found in a repetitive sequence within an intron (3 SNPs in intron 4) and were not likely to be involved in determining a more stable gamma-TMT allele.

Modifying genes affecting important traits for sunflower breeding such as high oleic acid content [24,25] and broomrape resistance [19] have been reported. The discovery of modifying genes affecting high gamma-tocopherol content confirms that the occurrence of modifying genes is not a rare phenomenon in sunflower genetics. Thus far there was no indication on the nature and mode of action of modifying genes. The results of the present research suggest that *Tph2* modifying genes are in most cases additional gamma-TMT loci duplicated in the sunflower genome. This was confirmed for modifying genes at LG 1 and 16, whereas there is also indication that the modifying gene at LG 14 might be an additional copy of a gamma-TMT. Duplicated gamma-TMT loci have also been found in safflower [26] and rapeseed [27]. In fact, the two gamma-TMT loci from safflower (one of them co-segregating with the safflower *Tph2* gene determining high gamma-tocopherol content in this crop) were identified by using the F9/R24 primer combination from the sunflower gamma-TMT [26], the same one used in this study to map different gamma-TMT loci. These results suggested that the F9 and R24 primer sequences are within a highly conserved region of the gamma-TMT gene. In sunflower, Hass et al. [14] mapped, in addition to *gamma-TMT-Tph2* on LG 8, another gamma-TMT locus on LG 16, although the authors considered this locus a randomly amplified polymorphic DNA (RAPD). In this study, the locus on LG 16 (gamma-TMT locus *b*) showed significant homology to the sunflower gamma-TMT gene and underlay a gamma-tocopherol QTL. Our results indicated that interaction between duplicated gamma-TMT loci revert the high gamma-tocopherol phenotype to intermediate-low gamma-tocopherol values. This effect has also been reported for modifying genes affecting the high oleic acid phenotype in sunflower, where modifying genes suppressed the effect of the *Ol-FAD2-1* allele that is essential for accumulating high oleic acid content [18,28]. Also, epistatically interacting duplicated MPBQ/MSBQ-MT genes that modify beta-tocopherol levels have been reported in sunflower [20]. It is well known that duplicate gene pairs can form negative epistasis due to their overlapping functions [29] and that suppression of a mutant phenotype can be altered by gene dosage [30]. However, additional biochemical, functional, and sequence analyses are required to determine the mode of action of duplicated gamma-TMT loci in sunflower.

From a breeding perspective, the existence of several modifying genes influencing high gamma-tocopherol content should not be a constraint for the development of cultivars with seeds rich in gamma-tocopherol, as this research revealed that modifying genes do not alter the phenotypic expression of the mutated allele *tph2* present at nmsT2100. On the other hand, the influence of modifying genes on expressivity of mutated *tph2*^*a*^ alleles opens up the possibility of selection for balanced levels of alpha- and gamma-tocopherol content, provided that stable combinations of modifying genes can be obtained. The development of mid oleic acid hybrids of sunflower was based on genetic stocks carrying modifying genes that limit the expression of high oleic acid content produced by mutated alleles at *Ol* locus (>80%) to the mid-range level (55–75%) [31]. In this sense, determination of allelic diversity at gamma-TMT loci underlying gamma-tocopherol modifying genes present in hybrid parental lines might be a useful tool to predict the presence of adequate allelic combinations giving rise to the desired levels of gamma-tocopherol content. Further studies should elucidate whether the other two gamma-tocopherol sources developed thus far, IAST-540 and LG-17, possess stable or unstable alleles at *Tph2*. Demurin et al. [6] reported differences in the expressivity of the recessive homozygotes of the *Tph2* gene from LG-17 in different genetic backgrounds, which might be indicative of allelic instability.

## Conclusions

The results of this research suggest that high gamma-tocopherol in sunflower lines IAST-1 and nmsT2100 is determined by different mutated alleles at the gamma-TMT *Tph2* locus on LG 8. The allele at nmsT2100 is not affected by modifying genes, whereas the allele at IAST-1 is unstable and affected by modifying genes. Putative modifying genes have been located at LG 1, 9, 14 and 16, being the effect of those on LG 1, 14 and 16 highly significant in their epistatic interaction with the *Tph2* locus on LG 8. Finally, our results suggest that modifying genes correspond to gamma-TMT loci duplicated in the sunflower genome. Phenotypic effects of modifying genes altering the expression of important genes in crop plants are known [23], but in most cases the genetic basis for modification remains unclear. In this study, we shed light into the mode of action and nature of modifier genes in sunflower, and suggest the relevance of duplicated loci affecting the expression of seed quality specific mutations.

## Methods

### Plant material, phenotypic analyses and DNA extraction

The study included the sunflower lines nmsT2100 and IAST-1, both with high gamma-tocopherol content (>85%). nmsT2100 is a NMS line developed by introgressing monogenic recessive NMS from line P21 [32] into high gamma-tocopherol line T2100 [10]. IAST-1 was isolated in the course of a chemical mutagenesis program on seeds of an accession of ‘Peredovik’ [11]. Twenty-four half seeds of nmsT2100 and IAST-1 were nondestructively analyzed for tocopherol profile as described below, germinated and planted in pots under open air conditions in the spring of 2005. NMS plants of nmsT2100 were pollinated with pollen of IAST-1 plants. Half seeds of the parents as well as F_1_ half seeds were analysed for tocopherol profile. F_1_ and parent half seeds were sown in March 2006 and the corresponding plants were grown in pots under open air conditions. F_1_ plants were bagged before flowering to obtain the F_2_ generation. F_2_ seeds from several F_1_ plants were analysed for seed tocopherol profile. In most cases, F_2_ seeds had uniformly high gamma-tocopherol content (>90%). However, some F_1_ plants showed segregation for gamma-tocopherol content at the F_2_ seed level, which indicated the expression of modifier genes. One population of 192 F_2_ seeds from a single F_1_ plant that showed large segregation for gamma-tocopherol content was selected for the molecular analyses. F_2_ half seeds were germinated and the corresponding plants were grown in pots under open air conditions in 2007. F_2_ plants were bagged before flowering to produce the F_3_ seed. Five fully expanded leaves from each F_2_ plant (135 F_2_ plants) were cut, frozen at −80°C, lyophilised and ground to a fine powder in a laboratory mill. DNA was isolated from ground leaf tissue from each F_2_ plant and from five plants of nmsT2100 and IAST-1 parental lines as described in Berry et al. [33]. Twenty four F_3_ seeds from each F_2_ plant that produced sufficient amount of seeds were analysed for tocopherol profile.

Two additional F_2_ populations were also used in this study with the objective of mapping tocopherol biosynthesis loci. One population, described in García-Moreno et al. [13], derived from a cross between CAS-12, with wild-type tocopherol profile mainly made up of alpha-tocopherol, and IAST-540, with high gamma-tocopherol content. The other population derived from a cross between lines IAST-413 and HA-89, both with wild-type tocopherol profile mainly made up of alpha-tocopherol, though IAST-413 is characterized by increased total tocopherol content [34].

The analysis of tocopherol profile was made for all analyzed generations and populations in half seeds. The half-seed technique is a common technique used in sunflower breeding consisting in cutting a small seed piece from the seed part distal to the embryo, which is used for nondestructive analysis of seed quality traits, as the remaining seed containing the embryo can be germinated after the corresponding analysis [35]. Individual half seeds were analysed for tocopherol profile following the method of Goffman et al. [36]. Half seeds were placed into 10-ml tubes with 2 ml iso-octane. The half seeds were then crushed with a stainless steel rod as fine as possible. The samples were stirred and extracted overnight at room temperature in darkness (extraction time about 16 h). After extraction, the samples were stirred again, centrifuged, and filtered. Twenty-five μl of the extract were analysed by HPLC using a fluorescence detector at 295 nm excitation and 330 nm emission and iso-octane/tert-butylmethylether (94:6) as eluent at an isocratic flow rate of 1 ml min^-1^. Chromatographic separation of the tocopherols was performed on a LiChrospher 100 diol column (250 mm x 2 mm I.D.) with 5-μm spherical particles, connected to a silica guard column (LiChrospher Si 60, 5 mm x 4 mm I.D.). The peak areas of the individual tocopherols were corrected according to their previously calculated response factors: alpha-tocopherol = 1.0; beta-tocopherol = 1.80; gamma-tocopherol = 1.85; delta-tocopherol = 2.30.

### Map construction and molecular analysis

A complete linkage map for the nmsT2100 x IAST-1 population was constructed to scan the genome for modifier genes affecting the expression of the *Tph2* gene. For this, the parental lines IAST-1 and nmsT2100 were initially screened for polymorphisms in two replicate samples together with four F_2_ individuals, using a genome-wide framework of 95 sunflower SSRs [37]. A preliminary genetic linkage map from this population was constructed. A set of INDEL markers [38], identified by ZVG prefixes, and an additional set of SSR markers mapped by Tang et al. [39] and Yu et al. [38], identified by ORS and CRT prefixes, were additionally screened for polymorphisms between these parental lines to complete the linkage map. INDEL markers for the tocopherol biosynthesis genes gamma-TMT, MPBQ/MSBQ-MT and tocopherol cyclase described by Hass et al. [14] and Tang et al. [20] were also screened for polymorphisms between nmsT2100 and IAST-1. PCRs for SSRs analyses were performed as described by Pérez-Vich et al. [40]. INDEL analyses were carried out following Yu et al. [38] and Hass et al. [14]. SSR and INDEL amplification products were separated on 3% (w/v) Metaphor® (BMA, Rockland, ME, USA) and 1.5% agarose gels, respectively, in 1x TBE buffer with ethidium bromide incorporated in the gel. SSR and INDEL markers revealing polymorphisms were then genotyped in the nmsT2100 x IAST-1 F_2_ population, following the protocols mentioned above.

Chi-square statistics were computed on each genotyped locus to detect deviations from the expected Mendelian ratios for codominant (1:2:1) or dominant (3:1) markers. The nmsT2100 x IAST-1 linkage map was constructed using the software MAPMAKER/EXP version 3.0b (Whitehead Institute, Cambridge, MA, USA) [41]. Two-point analysis was used to identify linkage groups (LGs) at a LOD score of 3 and a maximum recombination frequency of 0.40. Three-point and multi-point analyses were used to determine the order and interval distances between the markers at each LG. The Haldane mapping function was used to compute the map distances in centiMorgans (cM) from the recombination fractions. Multiple loci detected by a single maker were coded with the marker name plus the suffix a, b, c, or d to indicate each duplicate locus. Linkage group maps were drawn using the MapChart software [42].

Genetic analysis of modifier genes was performed in several stages. In the first stage, the significance of each marker’s association with the phenotypic trait [gamma-tocopherol content at the F_2_ seed and F_2_ plant (average value of 24 F_3_ seeds per F_2_ plant) generations] was determined by one-way analysis of variance (ANOVA) using the statistical package SPSS Statistics v. 19, with marker genotypes being classes. In this analysis, we identified an unexpected macromutation on LG 8 at the *Tph2* locus. The effects of the macromutations, if ignored, could dramatically reduce the power for identifying other genes or QTL affecting the studied trait.

In a second stage, composite interval mapping (CIM) [43,44] was used to scan the genome for QTL affecting gamma-tocopherol content, in order to strengthen and corroborate the results of the analyses of variance, evaluate the existence of additional QTL, and estimate the interaction and global effect of all the detected QTL. Computations were carried out using the software PLABQTL Version 1.1 [45]. The phenotypic data consisted on gamma-tocopherol content in the F_2_ seed and F_2_ plant generations. Additional analyses were carried out by using other parameters calculated from the F_3_ seed data such as the minimum, maximum and the standard deviation of gamma-tocopherol content in F_3_ seeds per F_2_ plant, and the number of F_3_ seeds within each F_2_ plant with less than 90% of gamma-tocopherol content. Since these analyses gave similar results to those obtained with the mean gamma-tocopherol value per F_3_ family, only results based on the F_3_ mean value are shown. Analyses were made initially with the “first” statement to check the database for errors and outliers. Next, simple interval mapping (SIM) was carried out for an initial scan and detection of QTL with main effects. Finally, CIM was performed with markers closest to the main QTL as co-factors. Genome-wide threshold values (α = 0.05) for declaring the presence of QTL were estimated from 1000 permutations of each phenotypic trait [46]. The thresholds of the LOD score (and their 0.95 confidence intervals) were 2.57 (2.48–2.70) and 2.65 (2.58–2.76) for gamma-tocopherol content in the F_2_ and the F_3_ generations, respectively. Estimates of QTL positions were obtained at the point where the LOD score reaches its maximum in the region under consideration. One-LOD support limits for the position of each QTL were also calculated [47]. The proportion of phenotypic variance explained by each individual QTL was calculated as the square of the partial correlation coefficient (*R*^*2*^). Estimates of the additive (a_i_) and dominance (d_i_) effects, as defined by Falconer [48], for the ith putative QTL, the total LOD score, as well as the total proportion of the phenotypic variance explained by all QTL, were obtained by fitting a multiple regression model including all putative QTL for the respective trait simultaneously [47]. The occurrence of QTL x QTL interactions was tested by adding digenic epistatic effects to the model.

QTL software such as PLABQTL estimate epistatic interactions among previously identified QTL. Since modifying genes are defined as genes having no known effect except to intensify or diminish the expression of a major gene [23], their effect as individual loci and subsequently their interaction with major loci may be undetectable with this type of analyses. In consequence, two-way interactions between the *Tph2* major locus and all the marker loci genotyped in this study were also tested. Two-way interactions were analyzed at a significance threshold of *P* ≤ 0.05 by analysis of variance using the general linear model (GLM) of SPSS Statistics v. 19. Statistical significance of differences for gamma-tocopherol content in different genotypes combining two marker loci were also computed using Duncan’s multiple range test. The significant epistatic interaction terms were combined with those of the previously identified QTL in multiple locus models using the “seq” statement of PLABQTL.

### Sequence analysis gamma-TMT loci

In the course of the genetic analyses of the nmsT2100 x IAST-1 population, we identified different gamma-TMT loci amplified with INDEL marker gamma-TMT-F9/R24 associated to gamma-tocopherol QTL. In order to confirm their nature, these loci were sequenced as follows. F9/R24 INDEL fragments amplified from the IAST-1 and nmsT2100 parental lines were separated on a 1.5% agarose gel, excised and purified by means of the QIAquick gel extraction kit (Qiagen GmbH, Hilden, Germany). The purified fragments were ligated into the T/A vector (pCR2.1) and the recombinants were transformed to TOP10 Chemically Competent *E. coli* using the TOPO-TA cloning kit (Invitrogen, San Diego, CA, USA) as described by the manufacturer. Five recombinant bacterial colonies (white) per isolated band were picked from the plate containing ampicillin and X-gal as selective media and cultured overnight at 37°C. Plasmids were extracted and purified using QIAprep Spin Miniprep Kit (Qiagen GmbH, Hilden, Germany). PCR with M13 forward and reverse vector primers and F9 and R24 primers, and restriction enzyme digestion was performed to confirm the presence and size of the insert. Sequencing in both forward and reverse orientations of the cloned fragments (two clones per locus) was performed at GATC Biotechnology (Konstanz, Germany) using the M13 forward and reverse sequencing primers. Sequence analysis was conducted with the aid of the software Vector NTI Advance 10.3.0 (Invitrogen, San Diego, CA, USA).

### Full-length sequence analysis of the IAST-1 and nmsT2100 gamma-TMT gene

Full-length gamma-TMT genomic DNA sequences were isolated from the high gamma-tocopherol lines nmsT2100 and IAST-1 by long distance PCR using primers developed at the 5´end (forward primer gamma-TMT-F1 from Hass et al. [14]) and the 3´end (reverse primer R92: TAATTCCTTGGGATGCCATT) of the sunflower gamma-TMT gene (GenBank accessions nos. DQ229828 to DQ229834). AccuPrime High Fidelity Taq DNA Polymerase (Invitrogen Life Technologies, Carlsbad, CA, USA) was used for PCR amplification in three individuals of nmsT2100 and five of IAST-1 as described by the manufacturer. The amplified products from each individual were separated on 1.5% agarose gels, showing in both lines two bands of a size higher than 4 kb that corresponded to the two gamma-TMT paralogs described by Hass et al. [14]. The upper (paralog 2) and the lower (paralog 1) bands were independently purified in each individual and cloned using the TOPO-TA cloning kit (Invitrogen Life Technologies, Carlsbad, CA, USA) as described above, with the exception that twenty recombinant bacterial colonies (white) per isolated band were picked from the plate. Restriction enzyme digestion was performed to confirm the presence of the insert and the restriction patterns characteristic for each gamma-TMT paralog. Sequencing in both forward and reverse orientations of the cloned fragments (a total of 10 clones for IAST-1 paralog 1, 13 clones for IAST-1 paralog 2, 5 clones for nmsT2100 paralog 1, and 6 clones for nmsT2100 paralog 2) was performed at GATC Biotechnology (Konstanz, Germany) using the universal M13 forward and reverse primers and internal primers (Gamma-TMT-F9, F27, F67, R10, R24, R35 and R78 from Hass et al. [14], and primers from Table 5) designed at 500 to 1000 bp intervals from the sunflower gamma-TMT gene. Sequence analysis was conducted using Vector NTI Advance 10.3.0 (Invitrogen, San Diego, CA, USA). A consensus sequence for IAST-1 gamma-TMT paralog 1, IAST-1 gamma-TMT paralog 2, nmsT2100 gamma-TMT paralog 1, and nmsT2100 gamma-TMT paralog 2 was made from the analysis of 10, 13, 5, and 6 sequenced clones, respectively. Changes in the nucleotide sequence were only included in the consensus sequence when they were conserved among the different clones and individuals sequenced from each line.

**Table 5:** Sequencing primers designed each 500–1000 bp into the gamma-TMT gene

**Primer**	**Sequence (5′-3′)**
F90	GGATGAATCGTTTGTTATTG
F91	GTCAATGGAGAGTGGAGAGC
F92	AGGAAGAAAAAATCTTGAATAA
F93	ATCGCTTCATCATCATCATA
F94	CACTAAATTTGACATCCACAAC
F95	GCCACTAATGATTGAAGGATT
R94	ACCACAACGTAAAAATGTTT
R95	CCACTACGTAGCAATGAAGT
R96	CCTTTAGTTTGCCAATTCAC
R97	CCGAGTCAACTCACTAACAA
R98	TCATTCACAAACTGCAGTAG

## Abbreviations

Gamma-TMT: gamma-tocopherol methyltransferase; INDEL: insertion-deletion; LG: linkage group; MPBQ/MSBQ-MT: 2-methyl-6-phytyl-1,4-benzoquinone/2-methyl-6-solanyl-1,4-benzoquinone methyltransferase; NMS: nuclear male sterile; SSR: simple sequence repeat.

## Authors’ contributions

MJG-M carried out genetic map construction and all other molecular analyses. JMF-M and LV selected the sunflower lines, crossed them, produced and studied the phenotypic data, and participated in the design of the study. BP-V conceived and designed the study and supervised molecular analyses and interpretation of results. All authors contributed to the manuscript preparation, and read and approved the final manuscript.

## Supplementary Material

Additional file 1 **Figure S1. Gamma-TMT sequence alignment.** Sunflower gamma-tocopherol methyltransferase genomic DNA sequence alignment for the high gamma-tocopherol lines IAST-1 and nmsT2100 as well as genbank sequences from gamma-TMT haplotypes 1 (DQ DQ229828), 2 (DQ DQ229829), 3 (DQ DQ229830), 4 (DQ DQ229831 and DQ DQ229832), and 5 (DQ DQ229833 and DQ DQ229834) and the cDNA gamma-TMT EF495161 sequence. Click here for file

## References

[B1] Eitenmiller R Lee J Vitamin E. Food Chemistry, Composition, and Analysis 2004 Marcel Dekker, New York

[B2] Hunter SC Cahoon EB Enhancing vitamin E in oilseeds: Unravelling tocopherol and tocotrienol biosynthesis Lipids 2007 42 97 108 10.1007/s11745-007-3028-6 17393215

[B3] Chow CK Stipanuk MH Vitamin E 2000 Saunders, Philadelphia 584 598

[B4] Kamal-Eldin A Appelqvist L-Å The chemistry and antioxidant properties of tocopherols and tocotrienols Lipids 1996 31 671 701 10.1007/BF02522884 8827691

[B5] Seppanen CM Song Q Csallany AS The antioxidant functions of tocopherol and tocotrienol homologues in oils, fats, and food systems Journal of the American Oil Chemists’ Society 2010 87 469 481 10.1007/s11746-009-1526-9

[B6] Demurin Y Skoric D Karlovic D Genetic variability of tocopherol composition in sunflower seeds as a basis of breeding for improved oil quality Plant Breeding 1996 115 33 36 10.1111/j.1439-0523.1996.tb00867.x

[B7] Fuster MD Lampi AM Hopia A Kamal-Eldin A Effects of alpha- and gamma-tocopherols on the autoxidation of purified sunflower triacylglycerols Lipids 1998 33 715 722 10.1007/s11745-998-0261-3 9688175

[B8] Yanishlieva NV Kamal-Eldin A Marinova EM Toneva AG Kinetics of antioxidant action of alpha- and gamma-tocopherols in sunflower and soybean triacylglycerols European Journal of Lipid Science and Technology 2002 104 262 270 10.1002/1438-9312(200205)104:5<262::AID-EJLT262>3.0.CO;2-B

[B9] Marmesat S Velasco L Ruiz-Méndez MV Fernández-Martínez JM Dobarganes C Thermostability of genetically modified sunflower oils differing in fatty acid and tocopherol compositions European Journal of Lipid Science and Technology 2008 110 776 782 10.1002/ejlt.200800040

[B10] Velasco L Dominguez J Fernández-Martínez JM Registration of T589 and T2100 sunflower germplasms with modified tocopherols Crop Sci 2004 44 362 363 10.2135/cropsci2004.0362

[B11] Velasco L Pérez-Vich B Fernández-Martínez JM Novel variation for tocopherol profile in a sunflower created by mutagenesis and recombination Plant Breeding 2004 123 490 492 10.1111/j.1439-0523.2004.01012.x

[B12] Velasco L Fernández-Martínez JM Identification and genetic characterization of new sources of beta- and gamma-tocopherol in sunflower germplasm Helia 2003 26 17 24 10.2298/HEL0338017V

[B13] García-Moreno MJ Vera-Ruiz EM Fernández-Martínez JM Velasco L Pérez-Vich B Genetic and molecular analysis of high gamma-tocopherol content in sunflower Crop Sci 2006 46 2015 2021 10.2135/cropsci2005.10.0388

[B14] Hass CG Tang S Leonard S Traber M Miller JF Knapp SJ Three non-allelic epistatically interacting methyltransferase mutations produce novel tocopherol (vitamin E) profiles in sunflower Theor Appl Genet 2006 113 767 782 10.1007/s00122-006-0320-4 16896719

[B15] Bergmüller E Porfirova S Dörmann P Characterization of an Arabidopsis mutant deficient in γ-tocopherol methyltransferase Plant Mol Biol 2003 52 1181 1190 14682617 10.1023/b:plan.0000004307.62398.91

[B16] Demurin Y Efimenko SG Peretyagina TM Genetic identification of tocopherol mutations in sunflower Helia 2004 27 113 116

[B17] García-Moreno MJ Fernández-Martínez JM Pérez-Vich B Velasco L Velasco L A modifying gene affecting gamma-tocopherol content in sunflower 2008 International Sunflower Association, Paris 601 604

[B18] Lacombe S Kaan F Léger L Bervillé A An oleate desaturase and a suppressor loci direct high oleic acid content of sunflower (Helianthus annuus L.) oil in the Pervenets mutant Comptes Rendus de l’Academie des Sciences, Series III, Sciences de la Vie 2001 324 839 845 10.1016/S0764-4469(01)01353-1 11558330

[B19] Velasco L Pérez-Vich B Jan CC Fernández-Martínez JM Inheritance of resistance to broomrape (Orobanche cumana Wallr.) race F in a sunflower line derived from wild sunflower species Plant Breeding 2007 126 67 71 10.1111/j.1439-0523.2006.01278.x

[B20] Tang S Hass C Knapp S Ty3/gypsy-like retrotransposon knockout of a 2-methyl-6-phytyl-1,4-benzoquinone methyltransferase is non-lethal, uncovers a cryptic paralogous mutation, and produces novel tocopherol (vitamin E) profiles in sunflower Theor Appl Genet 2006 113 783 799 10.1007/s00122-006-0321-3 16902787

[B21] Pérez-Vich B Berry ST Velasco L Fernández-Martínez JM Gandhi S Freeman C Heesacker A Knapp SJ Leon AJ Molecular mapping of nuclear male sterility genes in sunflower Crop Sci 2005 54 1851 1857

[B22] Del Moral L Fernández-Martínez JM Pérez-Vich B Velasco L Expression of modified tocopherol content and profile in sunflower tissues J Sci Food Agric 2012 92 351 357 10.1002/jsfa.4585 21815166

[B23] Briggs FD Knowles PF Introduction to Plant Breeding 1967 Reinhold Publishing Corporation, New York

[B24] Urie AL Inheritance of high oleic acid in sunflower Crop Sci 1985 25 986 989 10.2135/cropsci1985.0011183X002500060021x

[B25] Toulouse, France Paris: International Sunflower Association June 2000 2000 A31 A36

[B26] García-Moreno MJ Fernández-Martínez JM Velasco L Pérez-Vich B Molecular tagging and candidate gene analysis of the high gamma-tocopherol trait in safflower (Carthamus tinctorius L.) Mol Breed 2011 28 367 379 10.1007/s11032-010-9489-y

[B27] Endrigkeit J Wang X Cai D Zhang C Long Y Meng J Jung C Genetic mapping, cloning, and functional characterization of the BnaX.VTE4 gene encoding a gamma-tocopherol methyltransferase from oilseed rape Theor Appl Genet 2009 119 567 575 10.1007/s00122-009-1066-6 19479236

[B28] Fernández-Martínez JM Jiménez A Domínguez J García JM Garcés R Mancha M Genetic analysis of the high oleic content in cultivated sunflower (Helianthus annuus L.) Euphytica 1989 41 39 51 10.1007/BF00022409

[B29] Jiang H Gu Z Growth of novel epistatic interactions by gene duplication Genome Biology and Evolution 2011 3 295 301 10.1093/gbe/evr016 21402864 PMC3274824

[B30] Prelich G Suppresion mechanisms Trends in Genetics 1999 15 261 266 10.1016/S0168-9525(99)01749-7 10390624

[B31] Miller JF Vick BA Registration of four mid-range oleic acid sunflower genetic stocks Crop Sci 2002 42 994 10.2135/cropsci2002.0994

[B32] Jan CC Inheritance and allelism of mitomycin C- and streptomycin-induced recessive genes for male sterility in cultivated sunflower Crop Sci 1992 32 317 320 10.2135/cropsci1992.0011183X003200020006x

[B33] Berry ST Leon AJ Hanfrey CC Challis P Burkholz A Barnes SR Rufener GK Lee M Caligari PDS Molecular marker analysis of Helianthus annuus L. 2. Construction of an RFLP linkage map for cultivated sunflower Theor Appl Genet 1995 91 195 199 10.1007/BF00220877 24169763

[B34] Del Moralsnm> L Pérez-Vich B Fernández-Martínez JM Velasco L Inheritance of increased seed tocopherol content in sunflower line IAST-413 Plant Breeding 2011 130 540 543 10.1111/j.1439-0523.2011.01865.x

[B35] Fernández-Martínez JM Perez-Vich B Velasco L Vollmann J, Rajcan I Sunflower 2009 Springer, New York 155 232

[B36] Goffman FD Velasco L Thies W Quantitative determination of tocopherols in single seeds of rapeseed (Brassica napus L.) Fett-Lipid 1999 101 142 145 10.1002/(SICI)1521-4133(199904)101:4<142::AID-LIPI142>3.0.CO;2-J

[B37] Tang S Kishore VK Knapp SJ PCR-multiplexes for a genome-wide framework of simple sequence repeat marker loci in cultivated sunflower Theor Appl Genet 2003 107 6 19 12835928 10.1007/s00122-003-1233-0

[B38] Yu JK Tang S Slabaugh MB Heesacker A Cole G Herring M Soper J Han F Chu W-C Webb DM Thompson L Edwards KJ Berry S Leon AJ Grondona M Olungu C Maes N Knapp SJ Towards a saturated molecular genetic linkage map for cultivated sunflower Crop Sci 2003 43 367 387 10.2135/cropsci2003.0367

[B39] Tang S Yu JK Slabaugh MB Shintani DK Knapp SJ Simple sequence repeat map of the sunflower genome Theor Appl Genet 2002 105 1124 1136 10.1007/s00122-002-0989-y 12582890

[B40] Pérez-Vich B Akhtouch B Knapp SJ Leon AJ Velasco L Fernández-Martínez JM Berry ST Quantitative trait loci for broomrape (Orobanche cumana Wallr.) resistance in sunflower Theor Appl Genet 2004 109 92 102 10.1007/s00122-004-1599-7 14968309

[B41] Lander ES Green P Abrahamson J Barlow A Daly MJ Lincoln SE Newburg L MAPMAKER: An interactive computer package for constructing primary genetic linkage maps of experimental and natural populations Genomics 1987 1 174 181 10.1016/0888-7543(87)90010-3 3692487

[B42] Voorrips RE MapChart: Software for the graphical presentation of linkage maps and QTL J Hered 2002 93 77 78 10.1093/jhered/93.1.77 12011185

[B43] Jansen RC Stam P High resolution of quantitative traits into multiple loci via interval mapping Genetics 1994 136 1447 1455 8013917 10.1093/genetics/136.4.1447 PMC1205923

[B44] Zeng ZB Precision mapping of quantitative trait loci Genetics 1994 136 1457 1468 8013918 10.1093/genetics/136.4.1457 PMC1205924

[B45] Utz HF Melchinger AE PLABQTL: A program for composite interval mapping of QTL Journal of Quantitative Trait Loci 1996 2 1 5

[B46] Doerge RW Churchill GA Permutation tests for multiple loci affecting a quantitative character Genetics 1996 142 285 294 8770605 10.1093/genetics/142.1.285 PMC1206957

[B47] Bohn M Khairallah MM González-de-León D Hoisington DA Utz HF Deutsch JA Jewell DC Mihm JA Melchinger AE QTL mapping in tropical maize: I. Genomic regions affecting leaf feeding resistance to sugarcane borer and other traits Crop Sci 1996 36 1352 1361 10.2135/cropsci1996.0011183X003600050045x

[B48] Falconer DS Introduction to quantitative genetics 1989 3 Longman, London

