# Supplementary material for: Genetic basis of unstable expression of high gamma-tocopherol content in sunflower seeds
Source: BMC Plant Biol. 2012 May 18;12:71. doi: 10.1186/1471-2229-12-71 (PMC3463442; doi:10.1186/1471-2229-12-71)
Supplement: Additional file 1 — Figure S1. Gamma-TMT sequence alignment. Sunflower gamma-tocopherol methyltransferase genomic DNA sequence alignment for the high gamma-tocopherol lines IAST-1 and nmsT2100 as well as genbank sequences from gamma-TMT haplotypes 1 (DQ DQ229828), 2 (DQ DQ229829), 3 (DQ DQ229830), 4 (DQ DQ229831 and DQ DQ229832), and 5 (DQ DQ229833 and DQ DQ229834) and the cDNA gamma-TMT EF495161 sequence. [file 1471-2229-12-71-S1.pdf]

```
                                                                                                            Section 1
                                (1)   1        10        20        30        40        50        60                 79
EF495161 HELIANT-g-TMT_ mRNA    (1)   --------------------------------------ACGTGCCATTGTTGACACACATCACCACCACCACGCCAAA
DQ229828_F1R92                  (1)   ATGTCTATTTGAACACCACGTAAACTCTGTGAAGCTTTACGTGCCATTGTTGACACACATCACCACCACCACGCCAAA
DQ229829_F1R92                  (1)   ATGTCTATTTGAACACCACGTAAACTCTGTGAAGCTTTACGTGCCATTGTTGACACACATCACCACC------GCCAAA
DQ229830_F1R92                  (1)   ATGTCTATTTGAACACCACGTAAACTCTGTGAAGCTTTACGTGCCATTGTTGACACACGTCACCACCACCACGCCAAA
DQ229831_F1R92                  (1)   ATGTCTATTTGAACACCACGTAAACTCTGTGAAGCTTTACGTGCCATTGTTGACACACATCACCACCACCACGCCAAA
DQ229832_F1R92                  (1)   ATGTCTATTTGAACACCACGTAAACTCTGTGAAGCTTTACGTGCCATTGTTGACACACATCACCACCACCACGCCAAA
IAST-1_haplotype_4              (1)   ATGTCTATTTGAACACCACGTAAACTCTGTGAAGCTTTACGTGCCATTGTTGACACACATCACCACCACCACGCCAAA
nmsT2100_haplotype_4            (1)   ATGTCTATTTGAACACCACGTAAACTCTGTGAAGCTTTACGTGCCATTGTTGACACACATCACCACCACCACGCCAAA
DQ229833_F1R92                  (1)   ATGTCTATTTGAACACCACGTAAACTCTGTGAAGCTTTACGTGCCATTGTTGACACACGTCACCACCACCACGCCAAA
DQ229834_F1R92                  (1)   ATGTCTATTTGAACACCACGTAAACTCTGTGAAGCTTTACGTGCCATTGTTGACACACGTCACCACCACCACGCCAAA
IAST-1_haplotype_5              (1)   ATGTCTATTTGAACACCACGTAAACTCTGTGAAGCTTTACGTGCCATTGTTGACACACGTCACCACCACCACGCCAAA
nmsT2100_haplotype_5            (1)   ATGTCTATTTGAACACCACGTAAACTCTGTGAAGCTTTACGTGCCATTGTTGACACACATCACCACCACCACGCCAAA
Consensus                       (1)   ATGTCTATTTGAACACCACGTAAACTCTGTGAAGCTTTACGTGCCATTGTTGACACACATCACCACCACCACGCCAAA
                                                                                                            Section 2
                               (80)   80        90        100       110       120       130       140              158
EF495161 HELIANT-g-TMT_ mRNA   (42)   TTCACCACTCACTCACACAACCTGCTATGGCTACGACGGCAGTTGGCGTATCGGCGACGCCGATGACGGAGAAGCTGAC
DQ229828_F1R92                 (80)   TTCACCACTCACTCACACAACCTGCTATGGCTACGACGGCAGTTGGCGTATCGGCGACGCCGATGACGGAGAAGCTGAC
DQ229829_F1R92                 (74)   TTCACCACTCACTCACACAACCTGCTATGGCTACGACGGCAGTTGGCGTATCGGCGACGCCGATGACGGAGAAGCTGAC
DQ229830_F1R92                 (80)   TTCACCACTCACTCACACAACCTGCTATGGCTACGACGGCAGTTGGCGTATCGGCGACGCCGATGACGGAGAAGCTGAC
DQ229831_F1R92                 (80)   TTCACCACTCACTCACACAACCTGCTATGGCTACGACGGCAGTTGGCGTATCGGCGACGCCGATGACGGAGAAGCTGAC
DQ229832_F1R92                 (80)   TTCACCACTCACTCACACAACCTGCTATGGCTACGACGGCAGTTGGCGTATCGGCGACGCCGATGACGGAGAAGCTGAC
IAST-1_haplotype_4             (80)   TTCACCACTCACTCACACAACCTGCTATGGCTACGACGGCAGTTGGCGTATCGGCGACGCCGATGACGGAGAAGCTGAC
nmsT2100_haplotype_4           (80)   TTCACCACTCACTCACACAACCTGCTATGGCTACGACGGCAGTTGGCGTATCGGCGACGCCGATGACGGAGAAGCTGAC
DQ229833_F1R92                 (80)   TTCACCACTCACTCACACAACCTGCTATGGCTACGACGGCAGTTGGCGTATCGGCGACGCCGATGACGGAGAAGCTGAC
DQ229834_F1R92                 (80)   TTCACCACTCACTCACACAACCTGCTATGGCTACGACGGCAGTTGGCGTATCGGCGACGCCGATGACGGAGAAGCTGAC
IAST-1_haplotype_5             (80)   TTCACCACTCACTCACACAACCTGCTATGGCTACGACGGCAGTTGGCGTATCGGCGACGCCGATGACGGAGAAGCTGAC
nmsT2100_haplotype_5           (80)   TTCACCACTCACTCACACAACCTGCTATGGCTACGACGGCAGTTGGCGTATCGGCGACGCCGATGACGGAGAAGCTGAC
Consensus                      (80)   TTCACCACTCACTCACACAACCTGCTATGGCTACGACGGCAGTTGGCGTATCGGCGACGCCGATGACGGAGAAGCTGAC
```

Section 3

		159 170 180 190 200 210 220 237
	(159)	
EF495161 HELIANT-g-TMT_ mRNA	(121)	GGCGGCAGATGATGACCAGCAGCAGCAGAAGCTCAAAAAAGGAATCGCAGAGTTCTACGACGAATCCTCAGGTATGTGG
DQ229828_F1R92	(159)	GGCGGCAGATGATGACCAGCAACAGCAGAAGCTCAAAAAAGGAATCGCAGAGTTCTACGACGAATCCTCAGGTATGTGG
DQ229829_F1R92	(153)	GGCGGCAGATGATGACCAGCAACAGCAGAAGCTCAAAAAAGGAATCGCAGAGTTCTACGACGAATCCTCAGGTATGTGG
DQ229830_F1R92	(159)	GGCGGCAGATGATGACCAGCAACAGCAGAAGCTCAAAAAAGGAATCGCAGAGTTCTACGACGAATCCTCAGGTATGTGG
DQ229831_F1R92	(159)	GGCGGCAGATGATGACCAGCAGCAGCAGAAGCTCAAAAAAGGAATCGCAGAGTTCTACGACGAATCCTCAGGTATGTGG
DQ229832_F1R92	(159)	GGCGGCAGATGATGACCAGCAGCAGCAGAAGCTCAAAAAAGGAATCGCAGAGTTCTACGACGAATCCTCAGGTATGTGG
IAST-1_haplotype_4	(159)	GGCGGCAGATGATGACCAGCAGCAGCAGAAGCTCAAAAAAGGAATCGCAGAGTTCTACGACGAATCCTCAGGTATGTGG
nmsT2100_haplotype_4	(159)	GGCGGCAGATGATGACCAGCAGCAGCAGAAGCTCAAAAAAGGAATCGCAGAGTTCTACGACGAATCCTCAGGTATGTGG
DQ229833_F1R92	(159)	GGCGGCAGATGATGACCAGCAGCAGCAGAAGCTCAAAAAAGGAATCGCAGAGTTCTACGACGAATCCTCAGGTATGTGG
DQ229834_F1R92	(159)	GGCGGCAGATGATGACCAGCAGCAGCAGAAGCTCAAAAAAGGAATCGCAGAGTTCTACGACGAATCCTCAGGTATGTGG
IAST-1_haplotype_5	(159)	GGCGGCAGATGATGACCAGCAGCAGCAGAAGCTCAAAAAAGGAATCGCAGAGTTCTACGACGAATCCTCAGGTATGTGG
nmsT2100_haplotype_5	(159)	GGCGGCAGATGATGACCAGCAGCAGCAGAAGCTCAAAAAAGGAATCGCAGAGTTCTACGACGAATCCTCAGGTATGTGG
Consensus	(159)	GGCGGCAGATGATGACCAGCAGCAGCAGAAGCTCAAAAAAGGAATCGCAGAGTTCTACGACGAATCCTCAGGTATGTGG

Section 4

		238 250 260 270 280 290 300 316
	(238)	
EF495161 HELIANT-g-TMT_ mRNA	(200)	GAGAACATATGGGGAGAACACATGCATCACGGATATTATAACTCCGACGACGTCGTTGAACTCTCCGATCACCGTTCTG
DQ229828_F1R92	(238)	GAGAACATATGGGGAGAACACATGCATCACGGATATTATAACTCCGACGACGTCGTTGAACTCTCCGATCACCGTTCTG
DQ229829_F1R92	(232)	GAGAACATATGGGGAGAACACATGCATCACGGATATTATAACTCCGACGACGTCGTTGAACTCTCCGATCACCGTTCTG
DQ229830_F1R92	(238)	GAGAACATTTGGGGAGAACACATGCATCACGGATATTATAACTCCGACGACGTCGTTGAACTCTCCGATCACCGTTCTG
DQ229831_F1R92	(238)	GAGAACATATGGGGAGAACACATGCATCACGGATATTATAACTCCGACGACGTCGTTGAACTCTCCGATCACCGTTCTG
DQ229832_F1R92	(238)	GAGAACATATGGGGAGAACACATGCATCACGGATATTATAACTCCGACGACGTCGTTGAACTCTCCGATCACCGTTCTG
IAST-1_haplotype_4	(238)	GAGAACATATGGGGAGAACACATGCATCACGGATATTATAACTCCGACGACGTCGTTGAACTCTCCGATCACCGTTCTG
nmsT2100_haplotype_4	(238)	GAGAACATATGGGGAGAACACATGCATCACGGATATTATAACTCCGACGACGTCGTTGAACTCTCCGATCACCGTTCTG
DQ229833_F1R92	(238)	GAGAACATATGGGGAGAACACATGCATCACGGATATTATAACTCCGACGACGTCGTTGAACTCTCCGATCACCGTTCTG
DQ229834_F1R92	(238)	GAGAACATATGGGGAGAACACATGCATCACGGATATTATAACTCCGACGACGTCGTTGAACTCTCCGATCACCGTTCTG
IAST-1_haplotype_5	(238)	GAGAACATATGGGGAGAACACATGCATCACGGATATTATAACTCCGACGACGTCGTTGAACTCTCCGATCACCGTTCTG
nmsT2100_haplotype_5	(238)	GAGAACATATGGGGAGAACACATGCATCACGGATATTATAACTCCGACGACGTCGTTGAACTCTCCGATCACCGTTCTG
Consensus	(238)	GAGAACATATGGGGAGAACACATGCATCACGGATATTATAACTCCGACGACGTCGTTGAACTCTCCGATCACCGTTCTG

```
                                                                                                   Section 5
                          (317) 317          330       340       350       360       370       380            395
EF495161 HELIANT-g-TMT_ mRNA (279) CTCAGATCCGTATGATTGAACAAGCCCTAACGTTCGCCTCTGTTTCAG-------------------------------
        DQ229828_F1R92 (317) CTCAGATCCGTATGATTGAACAAGCCCTAACGTTCGCCTCTGTTTCAGGTAGTTATCAGTGGATTTATTCTGTTATTGT
        DQ229829_F1R92 (311) CTCAGATCCGTATGATTGAACAAGCCCTAACGTTCGCCTATGTTTCAGGTAGTTATCAGTTGATTTATTCTGTTATTGT
        DQ229830_F1R92 (317) CTCAGATCCGTATGATTGAACAAGCCCTAACGTTCGCCTCTGTTTCAGGTAGTTATCAGTTGATTTATTCTGTTATTGT
        DQ229831_F1R92 (317) CTCAGATCCGTATGATTGAACAAGCCCTAACGTTCGCCTCTGTTTCAGGTAGTTATCAGTGGATTTATTCTGTTATTGT
        DQ229832_F1R92 (317) CTCAGATCCGTATGATTGAACAAGCCCTAACGTTCGCCTCTGTTTCAGGTAGTTATCAGTGGATTTATTCTGTTATTGT
     IAST-1_haplotype_4 (317) CTCAGATCCGTATGATTGAACAAGCCCTAACGTTCGCCTCTGTTTCAGGTAGTTATCAGTGGATTTATTCTGTTATTGT
   nmsT2100_haplotype_4 (317) CTCAGATCCGTATGATTGAACAAGCCCTAACGTTCGCCTCTGTTTCAGGTAGTTATCAGTGGATTTATTCTGTTATTGT
        DQ229833_F1R92 (317) CTCAGATCCGTATGATTGAACAAGCCCTAACGTTCGCCTCTGTTTCAGGTAGTTATCAGTGGATTTATTCTGTTATTGT
        DQ229834_F1R92 (317) CTCAGATCCGTATGATTGAACAAGCCCTAACGTTCGCCTCTGTTTCAGGTAGTTATCAGTGGATTTATTCTGTTATTGT
     IAST-1_haplotype_5 (317) CTCAGATCCGTATGATTGAACAAGCCCTAACGTTCGCCTCTGTTTCAGGTAGTTATCAGTGGATTTATTCTGTTATTGT
   nmsT2100_haplotype_5 (317) CTCAGATCCGTATGATTGAACAAGCCCTAACGTTCGCCTCTGTTTCAGGTAGTTATCAGTGGATTTATTCTGTTATTGT
              Consensus (317) CTCAGATCCGTATGATTGAACAAGCCCTAACGTTCGCCTCTGTTTCAGGTAGTTATCAGTGGATTTATTCTGTTATTGT

                                                                                                   Section 6
                          (396) 396           410       420       430       440       450       460           474
EF495161 HELIANT-g-TMT_ mRNA (327) -------------------------------------------------------------------------------
        DQ229828_F1R92 (396) TTAATTGAATCTGACGGTTTAGAACTTTTAGATTTGTATAGATGAAATTATGAAAA-GCGCTTTCTGAACGGAGTTTTC
        DQ229829_F1R92 (390) TTAATTGAATCTGACGGTTTAGAACTTTTAGATTTGTATAGATGAAGTTATGAAAAAGCGGTTTCTGAACGGAGTTTTC
        DQ229830_F1R92 (396) TTAATTGAATCTGACGGTTTAGAACTTTTAGATTTGTATAGATGAAGTTATGAAAAAGCGGTTTCTGAACGGAGTTTTC
        DQ229831_F1R92 (396) TTAATTGAATCTGACGGTTTAGAACTTTTAGATTTGTATAGATGAAATTATGAAAA-GCGCTTTCTGAACGGAGTTTTC
        DQ229832_F1R92 (396) TTAATTGAATCTGACGGTTTAGAACTTTTAGATTTGTATAGATGAAATTATGAAAA-GCGCTTTCTGAACGGAGTTTTC
     IAST-1_haplotype_4 (396) TTAATTGAATCTGACGGTTTAGAACTTTTAGATTTGTATAGATGAAATTATGAAAA-GCGCTTTCTGAACGGAGTTTTC
   nmsT2100_haplotype_4 (396) TTAATTGAATCTGACGGTTTAGAACTTTTAGATTTGTATAGATGAAATTATGAAAA-GCGCTTTCTGAACGGAGTTTTC
        DQ229833_F1R92 (396) TTAATTGAATCTGACGGTTTAGAACTTTTAGATTTGTAT---------TATGAAAA-GCGCTTTCTGAACGGAGTTTTC
        DQ229834_F1R92 (396) TTAATTGAATCTGACGGTTTAGAACTTTTAGATTTGTAT---------TATGAAAA-GCGCTTTCTGAACGGAGTTTTC
     IAST-1_haplotype_5 (396) TTAATTGAATCTGACGGTTTAGAACTTTTAGATTTGTAT---------TATGAAAA-GCGGTGTCTGAACGGAGTTTTC
   nmsT2100_haplotype_5 (396) TTAATTGAATCTGACGGTTTAGAACTTTTAGATTTGTAT---------TATGAAAA-GCGGTGTCTGAACGGAGTTTTC
              Consensus (396) TTAATTGAATCTGACGGTTTAGAACTTTTAGATTTGTATAGATGAA TTATGAAAA GCGCTTTCTGAACGGAGTTTTC
```

```
                                                                                        Section 7
                            (475) 475  480       490       500       510       520       530       540      553
EF495161 HELIANT-g-TMT_ mRNA (327) -------------------------------------------------------------------------------
             DQ229828_F1R92 (474) GATGTTTCGATGCGGTTTTTGAGTTTTATAATTTGTACTCACTTTTTCTGAATGATTTTATTTGCTATTAGTTTCGATG
             DQ229829_F1R92 (469) GATGTTTCGATGCGGTTTTTGAGTTTTAAAATTTGTACTCACTTTTTCTGAATGATTTTATTTGCTATTAGTTTCGATG
             DQ229830_F1R92 (475) GATGTTTCGATGCGGTTTTTGAGTTTTAAAATTTGTACTCACTTTTTCTGAATGATTTTATTTGCTATTAGTTTCGATG
             DQ229831_F1R92 (474) GATGTTTCGATGCGGTTTTTGAGTTTTAAAATTTCTACTCACTTTTTCTGAATGATTTTATTTGCTATTAGTTTCGATG
             DQ229832_F1R92 (474) GATGTTTCGATGCGGTTTTTGAGTTTTAAAATTTCTACTCACTTTTTCTGAATGATTTTATTTGCTATTAGTTTCGATG
         IAST-1_haplotype_4 (474) GATGTTTCGATGCGGTTTTTGAGTTTTAAAATTTCTACTCACTTTTTCTGAATGATTTTATTTGCTATTAGTTTCGATG
       nmsT2100_haplotype_4 (474) GATGTTTCGATGCGGTTTTTGAGTTTTAAAATTTCTACTCACTTTTTCTGAATGATTTTATTTGCTATTAGTTTCGATG
             DQ229833_F1R92 (465) GA--------TGCGGTTTTTGAGTTTTATAATTTGTACTCACTTTTTCTGAATGATTTTATTTGCTATTAGTTTCGATG
             DQ229834_F1R92 (465) GA--------TGCGGTTTTTGAGTTTTATAATTTGTACTCACTTTTTCTGAATGATTTTATTTGCTATTAGTTTCGATG
         IAST-1_haplotype_5 (465) GA--------TGCGGTTTTTGAGTTTTATAATTTGTACTCACTTTTTCTGAATGATTTTATTTGCTATTAGTTTCGATG
       nmsT2100_haplotype_5 (465) GA--------TGCGGTTTTTGAGTTTTATAATTTGTACTCACTTTTTCTGAATGATTTTATTTGCTATTAGTTTCGATG
                  Consensus (475) GATGTTTCGATGCGGTTTTTGAGTTTTAAAATTTGTACTCACTTTTTCTGAATGATTTTATTTGCTATTAGTTTCGATG

                                                                                        Section 8
                            (554) 554    560       570       580       590       600       610       620      632
EF495161 HELIANT-g-TMT_ mRNA (327) -------------------------------------------------------------------------------
             DQ229828_F1R92 (553) GTGCTATAGTTGATTGTGAATGATATCCTTACTGATTATGTGTTTGTTATAAGATTTGTAATTGTTAATTAGGTTTTTG
             DQ229829_F1R92 (548) GTGCTATAGTTGATTGTGAATGATATCCTTACTGATTATGTGTTTGTTATAAGATTTGTAATTGTTAATTAGGTTTTTG
             DQ229830_F1R92 (554) GTGCTATAGTTGATTGTGAATGATATCCTTACTGATTATGTGTTTGTTATAAGATTTGTAATTGTTAATTAGGTTTTTG
             DQ229831_F1R92 (553) GTGCTATAGTTGATTGTGAATGATATCCTTACTGATTATGTGTTTGTTATAAGATTTGTAATTGTTAATTAGGTTTTTG
             DQ229832_F1R92 (553) GTGCTATAGTTGATTGTGAATGATATCCTTACTGATTATGTGTTTGTTATAAGATTTGTAATTGTTAATTAGGTTTTTG
         IAST-1_haplotype_4 (553) GTGCTATAGTTGATTGTGAATGATATCCTTACTGATTATGTGTTTGTTATAAGATTTGTAATTGTTAATTAGGTTTTTG
       nmsT2100_haplotype_4 (553) GTGCTATAGTTGATTGTGAATGATATCCTTACTGATTATGTGTTTGTTATAAGATTTGTAATTGTTAATTAGGTTTTTG
             DQ229833_F1R92 (536) GTGCTATAGTTGATTGTGAATGGTATCCTTACTGATTATGTGTTTGTTATAAGATTTGTAATTGTTAATTAGGTTTTTG
             DQ229834_F1R92 (536) GTGCTATAGTTGATTGTGAATGGTATCCTTACTGATTATGTGTTTGTTATAAGATTTGTAATTGTTAATTAGGTTTTTG
         IAST-1_haplotype_5 (536) GTGCTATAGTTGATTGTGAATGGTATCCTTACTGATTATGTGTTTGTTATAAGATTTGTAATTGTTAATTAGGTTTTTG
       nmsT2100_haplotype_5 (536) GTGCTATAGTTGATTGTGAATGGTATCCTTACTGATTATGTGTTTGTTATAAGATTTGTAATTGTTAATTAGGTTTTTG
                  Consensus (554) GTGCTATAGTTGATTGTGAATGATATCCTTACTGATTATGTGTTTGTTATAAGATTTGTAATTGTTAATTAGGTTTTTG
```

```
                                                                                    Section 9
                            (633) 633    640       650       660       670       680       690       700       711
 EF495161 HELIANT-g-TMT_ mRNA (327) -------------------------------------------------------------------------------
            DQ229828_F1R92 (632) AGAACTAAGTTTGAGTTATAGCTGAATAATCTATGCCGATTTTGAATTAGACGTGTGTTTTCGAACGGAGATTTCGAAG
            DQ229829_F1R92 (627) AGAACTAAGTTTGAGTTATAGCTGAATAATCTATGCCGATTTTGAATTAGACGTGTGTTTTCGAACGGAGGTTTCGAAG
            DQ229830_F1R92 (633) AGAACTAAGTTTGAGTTATAGCTGAATAATCTATGCCGATTTTGAATTAGACGTGTGTTTTCGAACGGAGGTTTCGAAG
            DQ229831_F1R92 (632) AGAACTATGTTTGAGTTATAGCTGAATAATCTATGCCGATTTTGAATTAGACGTGTGTTTTCGAATGGAGATTTCGAAG
            DQ229832_F1R92 (632) AGAACTATGTTTGAGTTATAGCTGAATAATCTATGCCGATTTTGAATTAGACGTGTGTTTTCGAATGGAGATTTCGAAG
        IAST-1_haplotype_4 (632) AGAACTATGTTTGAGTTATAGCTGAATAATCTATGCCGATTTTGAATTAGACGTGTGTTTTCGAATGGAGATTTCGAAG
      nmsT2100_haplotype_4 (632) AGAACTATGTTTGAGTTATAGCTGAATAATCTATGCCGATTTTGAATTAGACGTGTGTTTTCGAATGGAGATTTCGAAG
            DQ229833_F1R92 (615) AGAACCAAGTTTGAGTTATAGCTGAATAATCTATGCCGATTTTGAATTAGACGTGTGTTTTCGAACGGAGATTTCGAAG
            DQ229834_F1R92 (615) AGAACCAAGTTTGAGTTATAGCTGAATAATCTATGCCGATTTTGAATTAGACGTGTGTTTTCGAACGGAGATTTCGAAG
        IAST-1_haplotype_5 (615) AGAACCAAGTTTGAGTTATAGCTGAATAATCTATGCCGATTTTGAATTAGACGTGTGTTTTCGAACGGAGATTTCGAAG
      nmsT2100_haplotype_5 (615) AGAACTAAGTTTGAGTTATAGCTGAATAATCTATGCCGATTTTGAATTAGACGTGTGTTTTCGAACGGAGATTTCGAAG
                 Consensus (633) AGAACTAAGTTTGAGTTATAGCTGAATAATCTATGCCGATTTTGAATTAGACGTGTGTTTTCGAACGGAGATTTCGAAG

                                                                                    Section 10
                            (712) 712      720       730       740       750       760       770       780      790
 EF495161 HELIANT-g-TMT_ mRNA (327) -------------------------------------------------------------------------------
            DQ229828_F1R92 (711) TTTGATGCGGTTTCTGATTTACAAATATTTTATACTCACTTTTGGTGGATGAATCGTTTGTTATTGAATTTGCTAGTGC
            DQ229829_F1R92 (706) TTTGATGCGGTTTCTGATTTACAAATACTTTAGGCTCGCTTTTGGTGGATGAATCGTTTGTTATTGAATTTGCTAATGC
            DQ229830_F1R92 (712) TTTGATGCGGTTTCTGATTTACAAATACTTTAGGCTCGCTTTTGGTGGATGAATCGTTTGTTATTGAATTTGCTAATGC
            DQ229831_F1R92 (711) TTTGATGCGGTTTCTGATTTACAAATATTTTATACTCACTTTTGGTGGATGAATCGTTTGTTATTGAATTTGCTAATGC
            DQ229832_F1R92 (711) TTTGATGCGGTTTCTGATTTACAAATATTTTATACTCACTTTTGGTGGATGAATCGTTTGTTATTGAATTTGCTAATGC
        IAST-1_haplotype_4 (711) TTTGATGCGGTTTCTGATTTACAAATATTTTATACTCACTTTTGGTGGATGAATCGTTTGTTATTGAATTTGCTAATGC
      nmsT2100_haplotype_4 (711) TTTGATGCGGTTTCTGATTTACAAATATTTTATACTCACTTTTGGTGGATGAATCGTTTGTTATTGAATTTGCTAATGC
            DQ229833_F1R92 (694) TTTGATGCGGTTTCTGATTTACAAATATTTTATACTCACTTTTGGTGGATGAATCGTTTGTTATTGAATTTGCCTATGC
            DQ229834_F1R92 (694) TTTGATGCGGTTTCTGATTTACAAATATTTTATACTCACTTTTGGTGGATGAATCGTTTGTTATTGAATTTGCCTATGC
        IAST-1_haplotype_5 (694) TTTGATGCGGTTTCTGATTTACAAATATTTTATACTCACTTTTGGTGGATGAATCGTTTGTTATTGAATTTGCCTATGC
      nmsT2100_haplotype_5 (694) TTTGATGCGGTTTCTGATTTACAAATATTTTATACTCACTTTTGGTGGATGAATCGTTTGTTATTGAATTTGCCTATGC
                 Consensus (712) TTTGATGCGGTTTCTGATTTACAAATATTTTATACTCACTTTTGGTGGATGAATCGTTTGTTATTGAATTTGCTAATGC
```

```
                                                                                          Section 11
                             (791) 791      800       810       820       830       840       850                  869
EF495161 HELIANT-g-TMT_ mRNA (327) -------------------------------------------------------------------------------
             DQ229828_F1R92  (790) TACAATAGTTTGGGAATGATTGATTACAGGTTATGTGTTT-------GTTGTT---AGATTCATGACTGTCAATTAGGT
             DQ229829_F1R92  (785) TACAATAGTTTGGGAATGATTGGTTACAGGTTATGTGTTTTTTGTTAGTTGTTGTTAGATTCATGACTGTCAATTAGGT
             DQ229830_F1R92  (791) TACAATAGTTTGGGAATGATTGGTTACAGGTTATGTGTTTTTTGTTAGTTGTTGTTAGATTCATGACTGTCAATTAGGT
             DQ229831_F1R92  (790) TACAATAGTTTGTGAATGATTGGTTACAGGTTATGTGTTT-------GTTGTTGTTAGATTCATGACTGTCAATTAGGT
             DQ229832_F1R92  (790) TACAATAGTTTGTGAATGATTGGTTACAGGTTATGTGTTT-------GTTGTTGTTAGATTCATGACTGTCAATTAGGT
         IAST-1_haplotype_4  (790) TACAATAGTTTGTGAATGATTGGTTACAGGTTATGTGTTT-------GTTGTTGTTAGATTCATGACTGTCAATTAGGT
       nmsT2100_haplotype_4  (790) TACAATAGTTTGTGAATGATTGGTTACAGGTTATGTGTTT-------GTTGTTGTTAGATTCATGACTGTCAATTAGGT
             DQ229833_F1R92  (773) TACAATAGTTTGTGAATGATTGGTTACAGGTTATGTGTTTGTTGTTAGTTGTTGTTAGATTCATGACTGTCAATTAGGT
             DQ229834_F1R92  (773) TACAATAGTTTGTGAATGATTGGTTACAGGTTATGTGTTTGTTGTTAGTTGTTGTTAGATTCATGACTGTCAATTAGGT
         IAST-1_haplotype_5  (773) TACAATAGTTTGTGAATGATTGGTTACAGGTTATGTGTTTGTTGTTAGTTGTTGTTAGATTCATGACTGTCAATTAGGT
       nmsT2100_haplotype_5  (773) TACAATAGTTTGTGAATGATTGGTTACAGGTTATGTGTTTGTTGTTAGTTGTTGTTAGATTCATGACTGTCAATTAGGT
                  Consensus  (791) TACAATAGTTTGTGAATGATTGGTTACAGGTTATGTGTTT TTGTTAGTTGTTGTTAGATTCATGACTGTCAATTAGGT
                                                                                          Section 12
                             (870) 870       880       890       900       910       920       930               948
EF495161 HELIANT-g-TMT_ mRNA (327) -------------------------------------------------------------------------------
             DQ229828_F1R92  (859) TTTTGAAAATTGAAGTTGAATTCATGGCTGAAATCTCACAAGGATTGAAATTAAATGAAAATTGTACTTTTACTGAGTG
             DQ229829_F1R92  (864) TTTTGAAAATTGAAGTTGATTTCATGGCTGTAATCTCACAAGGATTGAAATTAAATGAAAATTGTACTTTTACTGAATG
             DQ229830_F1R92  (870) TTTTGAAAATTGAAGTTGATTTCATGGCTGTAATCTCACAAGGATTGAAATTAAATGAAAATTGTACTTTTACTGAATG
             DQ229831_F1R92  (862) TTTTGATAATTGAAGTTGAATTCATGGCTGGAATCTCACAAGGATTGAAATAAAC-GAAAATTGTACTTTTACTGAGTG
             DQ229832_F1R92  (862) TTTTGATAATTGAAGTTGAATTCATGGCTGGAATCTCACAAGGATTGAAATAAAC-GAAAATTGTACTTTTACTGAGTG
         IAST-1_haplotype_4  (862) TTTTGATAATTGAAGTTGAATTCATGGCTGGAATCTCACAAGGATTGAAATAAAC-GAAAATTGTACTTTTACTGAGTG
       nmsT2100_haplotype_4  (862) TTTTGATAATTGAAGTTGAATTCATGGCTGGAATCTCACAAGGATTGAAATAAAC-GAAAATTGTACTTTTACTGAGTG
             DQ229833_F1R92  (852) TTTTGAAAATTGAAGTTGATTTCATGGCTGTAATCTCACAAGGATTGAAATTAAATGAAAATTGTACTTTTACTGAGTG
             DQ229834_F1R92  (852) TTTTGAAAATTGAAGTTGATTTCATGGCTGTAATCTCACAAGGATTGAAATTAAATGAAAATTGTACTTTTACTGAGTG
         IAST-1_haplotype_5  (852) TTTTGAAAATTGAAGTTGATTTCATGGCTGTAATCTCACAAGGATTGAAATTAAATGAAAATTGTACTTTTACTGAGTG
       nmsT2100_haplotype_5  (852) TTTTGAAAATTGAAGTTGATTTCATGGCTGTAATCTCACAAGGATTGAAATTAAATGAAAATTGTACTTTTACTGAGTG
                  Consensus  (870) TTTTGAAAATTGAAGTTGATTTCATGGCTGTAATCTCACAAGGATTGAAATTAAATGAAAATTGTACTTTTACTGAGTG
```

Section 13

```
                            (949) 949        960       970       980       990       1000      1010            1027
EF495161 HELIANT-g-TMT_ mRNA (327) -------------------------------------------------------------------------------
            DQ229828_F1R92  (938) AACTCATTTGCTAATGCTACTGCAGTTTGTGAATGATATGCTCAGTGGCGAAGCTTGACCTAAAATTATACCTAAAAAT
            DQ229829_F1R92  (943) AATTCATTTGCTAATGCTACTGCAGTTTGTGAATGATATGGTCAGTGGCGAAGCTTGACCTAAAATTATACCTAAAAAA
            DQ229830_F1R92  (949) AATTCATTTGCTAATGCTACTGCAGTTTGTGAATGATATGGTCAGTGGCGAAGCTTGACCTAAAATTATACCTAAAAAA
            DQ229831_F1R92  (940) AACTCATTTGCTAATGCTACTGCAGTTTGTGAATGATATGGTCAATGGCGAAGCTTGACCTAAAATTATACCTAAAAAA
            DQ229832_F1R92  (940) AACTCATTTGCTAATGCTACTGCAGTTTGTGAATGATATGGTCAATGGCGAAGCTTGACCTAAAATTATACCTAAAAAA
          IAST-1_haplotype_4 (940) AACTCATTTGCTAATGCTACTGCAGTTTGTGAATGATATGGTCAATGGCGAAGCTTGACCTAAAATTATACCTAAAAAA
       nmsT2100_haplotype_4 (940) AACTCATTTGCTAATGCTACTGCAGTTTGTGAATGATATGGTCAATGGCGAAGCTTGACCTAAAATTATACCTAAAAAA
            DQ229833_F1R92  (931) AACTCATTTGCTAATGCTACTGCAGTTTGTGAATGATATGGTCAGTGGCGAAGCTTGACCTAAAATTATACCTAAAAAA
            DQ229834_F1R92  (931) AACTCATTTGCTAATGCTACTGCAGTTTGTGAATGATATGGTCAGTGGCGAAGCTTGACCTAAAATTATACCTAAAAAA
          IAST-1_haplotype_5 (931) AACTCATTTGCTAATGCTACTGCAGTTTGTGAATGATATGGTCAGTGGCGAAGCTTGACCTAAAATTATACCTAAAAAA
       nmsT2100_haplotype_5 (931) AACTCATTTGCTAATGCTACTGCAGTTTGTGAATGATATGGTCAGTGGCGAAGCTTGACCTAAAATTATACCTAAAAAA
                  Consensus (949) AACTCATTTGCTAATGCTACTGCAGTTTGTGAATGATATGGTCAGTGGCGAAGCTTGACCTAAAATTATACCTAAAAAA
```

Section 14

```
                           (1028) 1028        1040      1050      1060      1070      1080      1090            1106
EF495161 HELIANT-g-TMT_ mRNA (327) -------------------------------------------------------------------------------
            DQ229828_F1R92 (1017) TTATAAAACCGGGGGGACGAAAACGTATATACCTAAAAAATTATATACGAAAACTACATACCGGACACTACTGAGCGAA
            DQ229829_F1R92 (1022) TTATAAAACC-GGGGGACGAAAACGTATATACCTAAAAAATTCTATACGAAAACTACATACCGGACACTACTGAGCGAA
            DQ229830_F1R92 (1028) TTATAAAACC-GGGGGACGAAAACGTATATACCTAAAAAATTCTATACGAAAACTACATACCGGACACTACTGAGCGAA
            DQ229831_F1R92 (1019) TTATAAAACTGGGGGGACGAAAACGTATATACCTAAAAAATTCTATACGAAAACTACATACCGGACACTACTGAGCGAA
            DQ229832_F1R92 (1019) TTATAAAACTGGGGGGACGAAAACGTATATACCTAAAAAATTCTATACGAAAACTACATACCGGACACTACTGAGCGAA
          IAST-1_haplotype_4 (1019) TTATAAAACTGGGGGGACGAAAACGTATATACCTAAAAAATTCTATACGAAAACTACATACCGGACACTACTGAGCGAA
       nmsT2100_haplotype_4 (1019) TTATAAAACTGGGGGGACGAAAACGTATATACCTAAAAAATTCTATACGAAAACTACATACCGGACACTACTGAGCGAA
            DQ229833_F1R92 (1010) TTATAAAACCGGGGGGACGAAAACGTATATACCTAAAAAATTCTATACGAAAACTACATACTGGACACTACTGAGCGAA
            DQ229834_F1R92 (1010) TTATAAAACCGGGGGGACGAAAACGTATATACCTAAAAAATTCTATACGAAAACTACATACTGGACACTACTGAGCGAA
          IAST-1_haplotype_5 (1010) TTATAAAACCGGGGGGACGAAAACGTATATACCTAAAAAATTCTATACGAAAACTACATACTGGACACTACTGAGCGAA
       nmsT2100_haplotype_5 (1010) TTATAAAACCGGGGGGACGAAAACGTATATACCTAAAAAATTCTATACGAAAACTACATACTGGACACTACTGAGCGAA
                 Consensus (1028) TTATAAAACCGGGGGGACGAAAACGTATATACCTAAAAAATTCTATACGAAAACTACATACCGGACACTACTGAGCGAA
```

Section 15

```
                              (1107) 1107         1120      1130      1140      1150      1160      1170          1185
EF495161 HELIANT-g-TMT_ mRNA   (327) -------------------------------------------------------------------------------
          DQ229828_F1R92 (1096) AAGTTCGGAGGGTCGGACGCCCCCCGGCCCCCACTATGCTACGCCCATGGATATGGTTACAGATTATGTATTTGTTATT
          DQ229829_F1R92 (1100) AAGTTCGGAGGGTCGGACGCCCCCCGGCCCCCACTATGCTACGCCCATGGATATGGTTACAGATTATGTATTTGTTATT
          DQ229830_F1R92 (1106) AAGTTCGGAGGGTCGGACGCCCCCCGGCCCCCACTATGCTACGCCCATGGATATGGTTACAGATTATGTATTTGTTATT
          DQ229831_F1R92 (1098) AAGTTCGGAGGGTCGGACGCCCCCCGGCCCCCACTATGCTACGCTCATGGATATGGTTACAGATTATGTATTTGTTATT
          DQ229832_F1R92 (1098) AAGTTCGGAGGGTCGGACGCCCCCCGGCCCCCACTATGCTACGCTCATGGATATGGTTACAGATTATGTATTTGTTATT
     IAST-1_haplotype_4 (1098) AAGTTCGGAGGGTCGGACGCCCCCCGGCCCCCACTATGCTACGCTCATGGATATGGTTACAGATTATGTATTTGTTATT
   nmsT2100_haplotype_4 (1098) AAGTTCGGAGGGTCGGACGCCCCCCGGCCCCCACTATGCTACGCTCATGGATATGGTTACAGATTATGTATTTGTTATT
          DQ229833_F1R92 (1089) AAGTTCGGAGGGTCGGACGCCCCTCGGCCCCCACTATGCTACGCCTATGGATATGGTTACTGATTATGTATTTGTTATT
          DQ229834_F1R92 (1089) AAGTTCGGAGGGTCGGACGCCCCTCGGCCCCCACTATGCTACGCCTATGGATATGGTTACTGATTATGTATTTGTTATT
     IAST-1_haplotype_5 (1089) AAGTTCGGAGGGTCGGACGCCCCTCGGCCCCCACTATGCTACGCCTATGGATATGGTTACTGATTATGTATTTGTTATT
   nmsT2100_haplotype_5 (1089) AAGTTCGGAGGGTCGGACGCCCCTCGGCCCCCACTATGCTACGCCTATGGATATGGTTACTGATTATGTATTTGTTATT
               Consensus (1107) AAGTTCGGAGGGTCGGACGCCCCCCGGCCCCCACTATGCTACGCCCATGGATATGGTTACAGATTATGTATTTGTTATT
```

Section 16

```
                              (1186) 1186           1200      1210      1220      1230      1240      1250          1264
EF495161 HELIANT-g-TMT_ mRNA   (327) -------------------------------------------------------------------------------
          DQ229828_F1R92 (1175) AGTTTTTGTTTCATGACTGTCAATTAGGTTTTTGAAGATTAGCTTTGAATTAATGGTGAAATTTTTCAGGATTGAAAGT
          DQ229829_F1R92 (1179) AGTTTTTGTTTCATGACTGTCAATTAGGTTTTTGAAGATTAGCTTTG--------------------------------
          DQ229830_F1R92 (1185) AGTTTTTGTTTCATGACTGTCAATTAGGTTTTTGAAGATTAGCTTTG--------------------------------
          DQ229831_F1R92 (1177) AGTTTTTGTTTCATGACTGTCAATTAGGTTTTTGAAGATTAGCTTTGAATTAATGGTGAAATTTTTCAGGATTGAAAGT
          DQ229832_F1R92 (1177) AGTTTTTGTTTCATGACTGTCAATTAGGTTTTTGAAGATTAGCTTTGAATTAATGGTGAAATTTTTCAGGATTGAAAGT
     IAST-1_haplotype_4 (1177) AGTTTTTGTTTCATGACTGTCAATTAGGTTTTTGAAGATTAGCTTTGAATTAATGGTGAAATTTTTCAGGATTGAAAGT
   nmsT2100_haplotype_4 (1177) AGTTTTTGTTTCATGACTGTCAATTAGGTTTTTGAAGATTAGCTTTGAATTAATGGTGAAATTTTTCAGGATTGAAAGT
          DQ229833_F1R92 (1168) AGTTTTTGTTTCATGACTGTCAATTAGGTTATTGAAGATTAGCTTTGAATTAATGGTGAAATTTTTCAGGATTGAAAGT
          DQ229834_F1R92 (1168) AGTTTTTGTTTCATGACTGTCAATTAGGTTATTGAAGATTAGCTTTGAATTAATGGTGAAATTTTTCAGGATTGAAAGT
     IAST-1_haplotype_5 (1168) AGTTTTTGTTTCATGACTGTCAATTAGGTTATTGAAGATTAGCTTTGAATTAATGGTGAAATTTTTCAGGATTGAAAGT
   nmsT2100_haplotype_5 (1168) AGTTTTTGTTTCATGACTGTCAATTAGGTTATTGAAGATTAGCTTTGAATTAATGGTGAAATTTTTCAGGATTGAAAGT
               Consensus (1186) AGTTTTTGTTTCATGACTGTCAATTAGGTTTTTGAAGATTAGCTTTGAATTAATGGTGAAATTTTTCAGGATTGAAAGT
```

```
                                                                                                    Section 17
                           (1265) 1265 1270      1280      1290      1300      1310      1320      1330      1343
EF495161 HELIANT-g-TMT_ mRNA (327) -------------------------------------------------------------------------------
           DQ229828_F1R92 (1254) TGAAATTAAATAACTTGTCGGATGCAAATTATACTTTGAATGTACTGTCAAAAAAAAGTACTGTCACATTAAGAGTGTT
           DQ229829_F1R92 (1226) -------------------------------------------------------------------------------
           DQ229830_F1R92 (1232) -------------------------------------------------------------------------------
           DQ229831_F1R92 (1256) TGAAATTAAATAACTTGTCGGATGCAAATTATACTTTTAATATACTGTCAAAAAAAAGTACTGTCACATTAAGAGTGTT
           DQ229832_F1R92 (1256) TGAAATTAAATAACTTGTCGGATGCAAATTATACTTTTAATATACTGTCAAAAAAAAGTACTGTCACATTAAGAGTGTT
       IAST-1_haplotype_4 (1256) TGAAATTAAATAACTTGTCGGATGCAAATTATACTTTTAATATACTGTCAAAAAAAAGTACTGTCACATTAAGAGTGTT
     nmsT2100_haplotype_4 (1256) TGAAATTAAATAACTTGTCGGATGCAAATTATACTTTTAATATACTGTCAAAAAAAAGTACTGTCACATTAAGAGTGTT
           DQ229833_F1R92 (1247) TGAAATTAAATAACTTGTCGGATGCAAATTATACTTTTAATGTACTGTCAAAAAAAAGTACTGTCACATTAAGAGTGTT
           DQ229834_F1R92 (1247) TGAAATTAAATAACTTGTCGGATGCAAATTATACTTTTAATGTACTGTCAAAAAAAAGTACTGTCACATTAAGAGTGTT
       IAST-1_haplotype_5 (1247) TGAAATTAAATAACTTGTCGGATGCAAATTATACTTTTAATGTACTGTCAAAAAAAAGTACTGTCACATTAAGAGTGTT
     nmsT2100_haplotype_5 (1247) TGAAATTAAATAACTTGTCGGATGCAAATTATACTTTTAATGTACTGTCAAAAAAAAGTACTGTCACATTAAGAGTGTT
                Consensus (1265) TGAAATTAAATAACTTGTCGGATGCAAATTATACTTTTAAT TACTGTCAAAAAAAAGTACTGTCACATTAAGAGTGTT
                                                                                                    Section 18
                           (1344) 1344 1350      1360      1370      1380      1390      1400      1410      1422
EF495161 HELIANT-g-TMT_ mRNA (327) -------------------------------------------------------------------------------
           DQ229828_F1R92 (1333) TTCTTACAGTAGATTGTGATTTGTCAGTCTTGTGATTGTGACTTGTGACTCCCTCACTTACCATTTTGCCCTTCGTTGC
           DQ229829_F1R92 (1226) -----------------------TCAGTCTTGTGATTGTGACTTGTGACTCCCTCACTTACCATTTTGCCCTTCGTTGC
           DQ229830_F1R92 (1232) -----------------------TCAGTCTTGTGATTGTGACTTGTGACTCCCTCACTTACCATTTTGCCCTTCGTTGC
           DQ229831_F1R92 (1335) TTCTTACAGTAGATTGTGATTTGTCAGTCTTGTGATTGTGGCTTGTGACTCCCTCACTTACGATTTTGCCCTTCGTTGC
           DQ229832_F1R92 (1335) TTCTTACAGTAGATTGTGATTTGTCAGTCTTGTGATTGTGGCTTGTGACTCCCTCACTTACGATTTTGCCCTTCGTTGC
       IAST-1_haplotype_4 (1335) TTCTTACAGTAGATTGTGATTTGTCAGTCTTGTGATTGTGGCTTGTGACTCCCTCACTTACGATTTTGCCCTTCGTTGC
     nmsT2100_haplotype_4 (1335) TTCTTACAGTAGATTGTGATTTGTCAGTCTTGTGATTGTGGCTTGTGACTCCCTCACTTACGATTTTGCCCTTCGTTGC
           DQ229833_F1R92 (1326) TTCTTACAGTAGATTGTGATTTGTCAGTCTTGTGATTTTGACTTGTGACTCCCTCGCTTACCATTTTGCCATTCGTTGC
           DQ229834_F1R92 (1326) TTCTTACAGTAGATTGTGATTTGTCAGTCTTGTGATTTTGACTTGTGACTCCCTCGCTTACCATTTTGCCATTCGTTGC
       IAST-1_haplotype_5 (1326) TTCTTACAGTAGATTGTGATTTGTCAGTCTTGTGATTTTGACTTGTGACTCCCTCGCTTACCATTTTGCCATTCGTTGC
     nmsT2100_haplotype_5 (1326) TTCTTACAGTAGATTGTGATTTGTCAGTCTTGTGATTTTGACTTGTGACTCCCTCGCTTACCATTTTGCCATTCGTTGC
                Consensus (1344) TTCTTACAGTAGATTGTGATTTGTCAGTCTTGTGATTGTGACTTGTGACTCCCTCACTTACCATTTTGCCCTTCGTTGC
```

```
                                                                                                    Section 19
                                 (1423) 1423   1430       1440       1450       1460       1470       1480       1490     1501
EF495161 HELIANT-g-TMT_ mRNA      (327) --ATGATCCGGAAAAGAAACCTAAAACCATAGTTGATGTCGGGTGTGGTATAGGAGGTAGTTCAAGGTATCTAGCAAGA
              DQ229828_F1R92     (1412) AGATGATCCGGAAAAGAAACCTAAAACAATAGTTGATGTCGGGTGTGGTATAGGAGGTAGCTCAAGGTATCTAGCAAGA
              DQ229829_F1R92     (1282) AGATGATCCGGAAAAGAAACCTAAAACCATAGTTGATGTCGGGTGTGGTATAGGAGGTAGCTCAAGGTATCTAGCAAGA
              DQ229830_F1R92     (1288) AGATGATCCGGAAAAGAAACCTAAAACCATAGTTGATGTCGGGTGTGGTATAGGAGGTAGCTCAAGGTATCTAGCAAGA
              DQ229831_F1R92     (1414) AGATGATCTGGAAAAGAAACCTAAAACCATAGTTGATGTCGGGTGTGGTATAGGAGGTAGCTCAAGGTATCTAGCAAGA
              DQ229832_F1R92     (1414) AGATGATCTGGAAAAGAAACCTAAAACCATAGTTGATGTCGGGTGTGGTATAGGAGGTAGCTCAAGGTATCTAGCAAGA
          IAST-1_haplotype_4     (1414) AGATGATCTGGAAAAGAAACCTAAAACCATAGTTGATGTCGGGTGTGGTATAGGAGGTAGCTCAAGGTATCTAGCAAGA
        nmsT2100_haplotype_4     (1414) AGATGATCTGGAAAAGAAACCTAAAACCATAGTTGATGTCGGGTGTGGTATAGGAGGTAGCTCAAGGTATCTAGCAAGA
              DQ229833_F1R92     (1405) AGATGATCCGGAAAAGAAACCTAAAACCATAGTTGATGTCGGGTGTGGTATAGGAGGTAGTTCAAGGTATCTAGCAAGA
              DQ229834_F1R92     (1405) AGATGATCCGGAAAAGAAACCTAAAACCATAGTTGATGTCGGGTGTGGTATAGGAGGTAGTTCAAGGTATCTAGCAAGA
          IAST-1_haplotype_5     (1405) AGATGATCCGGAAAAGAAACCTAAAACCATAGTTGATGTCGGGTGTGGTATAGGAGGTAGTTCAAGGTATCTAGCAAGA
        nmsT2100_haplotype_5     (1405) AGATGATCCGGAAAAGAAACCTAAAACCATAGTTGATGTCGGGTGTGGTATAGGAGGTAGTTCAAGGTATCTAGCAAGA
                   Consensus     (1423) AGATGATCCGGAAAAGAAACCTAAAACCATAGTTGATGTCGGGTGTGGTATAGGAGGTAGCTCAAGGTATCTAGCAAGA

                                                                                                    Section 20
                                 (1502) 1502    1510       1520       1530       1540       1550       1560       1570     1580
EF495161 HELIANT-g-TMT_ mRNA      (404) AAATACGGAGCCGAATGTCACGGAATCACCCTCAGCCCTGTGCAAGCTGAGAGAGCTAATGCCCTTGCTGCGGCCCAAG
              DQ229828_F1R92     (1491) AAATACGGAGCCGAATGTCACGGAATCACCCTCAGCCCTGTGCAAGCTGAGAGAGCTAATGCCCTTGCTGCGGCCCAAG
              DQ229829_F1R92     (1361) AAATACGGAGCCGAATGTCACGGAATCACCCTCAGCCCTGTGCAAGCTGAGAGAGCTAATGCCCTTGCTGCGGCCCAAG
              DQ229830_F1R92     (1367) AAATACGGAGCCGAATGTCACGGAATCACCCTCAGCCCTGTGCAAGCTGAGAGAGCTAATGCCCTTGCTGCGGCCCAAG
              DQ229831_F1R92     (1493) AAATACGGAGCCGAATGTCACGGAATCACCCTCAGCCCTGTGCAAGCTGAGAGAGCTAATGCCCTTGCTGCGGCCCAAG
              DQ229832_F1R92     (1493) AAATACGGAGCCGAATGTCACGGAATCACCCTCAGCCCTGTGCAAGCTGAGAGAGCTAATGCCCTTGCTGCGGCCCAAG
          IAST-1_haplotype_4     (1493) AAATACGGAGCCGAATGTCACGGAATCACCCTCAGCCCTGTGCAAGCTGAGAGAGCTAATGCCCTTGCTGCGGCCCAAG
        nmsT2100_haplotype_4     (1493) AAATACGGAGCCGAATGTCACGGAATCACCCTCAGCCCTGTGCAAGCTGAGAGAGCTAATGCCCTTGCTGCGGCCCAAG
              DQ229833_F1R92     (1484) AAATACGGAGCCGAATGTCACGGAATCACCCTCAGCCCTGTGCAAGCTGAGAGAGCTAATGCCCTTGCTGCGGCCCAAG
              DQ229834_F1R92     (1484) AAATACGGAGCCGAATGTCACGGAATCACCCTCAGCCCTGTGCAAGCTGAGAGAGCTAATGCCCTTGCTGCGGCCCAAG
          IAST-1_haplotype_5     (1484) AAATACGGAGCCGAATGTCACGGAATCACCCTCAGCCCTGTGCAAGCTGAGAGAGCTAATGCCCTTGCTGCGGCCCAAG
        nmsT2100_haplotype_5     (1484) AAATACGGAGCCGAATGTCACGGAATCACCCTCAGCCCTGTGCAAGCTGAGAGAGCTAATGCCCTTGCTGCGGCCCAAG
                   Consensus     (1502) AAATACGGAGCCGAATGTCACGGAATCACCCTCAGCCCTGTGCAAGCTGAGAGAGCTAATGCCCTTGCTGCGGCCCAAG
```

```
Section 21
                                (1581) 1581     1590      1600      1610      1620      1630      1640              1659
EF495161 HELIANT-g-TMT_ mRNA     (483) GGTTGGCCGATAAG-----------------------------------------------------------------
DQ229828_F1R92                  (1570) GGTTGGCCGATAAGGTACCGCAGTTTGTCAAGATTTTTGGGG-AGAATATTGCACGTCAATTGTTCTATTTGACTTTTT
DQ229829_F1R92                  (1440) GGTTGGCCGATAAGGTACCACAGTTTGTCAATATTTTTGGGG-AGAATATTGCACATCAATTGTTCTATTTGACTTTTT
DQ229830_F1R92                  (1446) GGTTGGCCGATAAGGTACCACAGTTTGTCAATATTTTTGGGG-AGAATATTGCACATCAATTGTTCTATTTGACTTTTT
DQ229831_F1R92                  (1572) GGTTGGCCGATAAGGTACCGCAGT----CAAGATTTTTGGGG-AGAATATTGCACATCAATTCTTCTATTTGACTTTTT
DQ229832_F1R92                  (1572) GGTTGGCCGATAAGGTACCGCAGT----CAAGATTTTTGGGG-AGAATATTGCACATCAATTCTTCTATTTGACTTTTT
IAST-1_haplotype_4              (1572) GGTTGGCCGATAAGGTACCGCAGT----CAAGATTTTTGGGG-AGAATATTGCACATCAATTCTTCTATTTGACTTTTT
nmsT2100_haplotype_4            (1572) GGTTGGCCGATAAGGTACCGCAGT----CAAGATTTTTGGGG-AGAATATTGCACATCAATTCTTCTATTTGACTTTTT
DQ229833_F1R92                  (1563) GGTTGGCCGATAAGGTACCACAGTTTGTCAAGATTTTTGGGGGAGAATATTGCACATCAATTCTTCTATTTGACTTTTT
DQ229834_F1R92                  (1563) GGTTGGCCGATAAGGTACCACAGTTTGTCAAGATTTTTGGGGGAGAATATTGCACATCAATTCTTCTATTTGACTTTTT
IAST-1_haplotype_5              (1563) GGTTGGCCGATAAGGTACCACAGTTTGTCAAGATTTTTGGGGGAGAATATTGCACATCAATTCTTCTATTTGACTTTTT
nmsT2100_haplotype_5            (1563) GGTTGGCCGATAAGGTACCACAGTTTGTCAAGATTTTTGGGGGAGAATATTGCACATCAATTCTTCTATTTGACTTTTT
Consensus                       (1581) GGTTGGCCGATAAGGTACCACAGTTTGTCAAGATTTTTGGGG AGAATATTGCACATCAATTCTTCTATTTGACTTTTT

Section 22
                                (1660) 1660       1670      1680      1690      1700      1710      1720             1738
EF495161 HELIANT-g-TMT_ mRNA     (497) -------------------------------------------------------------------------------
DQ229828_F1R92                  (1648) GCACATCAAATTCGTATAATTGAGTCGAGCTATCTCTAAAAGCCTGCACACATACTACGATTATTAACGAAGTTAACTG
DQ229829_F1R92                  (1518) GCACATCAAATTCGTATAATTGAGTCGAGCTATCTCTAAAAGCCTGCACACGTAATACGATTATTAACGAAGTCAACTG
DQ229830_F1R92                  (1524) GCACATCAAATTCGTATAATTGAGTCGAGCTATCTCTAAAAGCCTGCACACGTAATACGATTATTAACGAAGTCAACTG
DQ229831_F1R92                  (1646) GCACATCAAATTCGTATAATTGAGTCGAGCTATCTCTAAAAGCCTGCACACATAATACGATTATTAACAAAGTCAACTG
DQ229832_F1R92                  (1646) GCACATCAAATTCGTATAATTGAGTCGAGCTATCTCTAAAAGCCTGCACACATAATACGATTATTAACAAAGTCAACTG
IAST-1_haplotype_4              (1646) GCACATCAAATTCGTATAATTGAGTCGAGCTATCTCTAAAAGCCTGCACACATAATACGATTATTAACAAAGTCAACTG
nmsT2100_haplotype_4            (1646) GCACATCAAATTCGTATAATTGAGTCGAGCTATCTCTAAAAGCCTGCACACATAATACGATTATTAACAAAGTCAACTG
DQ229833_F1R92                  (1642) GCACATCAAATTCGTATAATTGAGT--AGCTATCTCTAAAAGCCTGTACACATACTACGATTATTAACGAAGTTAACTG
DQ229834_F1R92                  (1642) GCACATCAAATTCGTATAATTGAGT--AGCTATCTCTAAAAGCCTGTACACATACTACGATTATTAACGAAGTTAACTG
IAST-1_haplotype_5              (1642) GCACATCAAATTCGTATAATTGAGT--AGCTATCTCTAAAAGCCTGTACACATACTACGATTATTAACGAAGTTAACTG
nmsT2100_haplotype_5            (1642) GCACATCAAATTCGTATAATTGAGT--AGCTATCTCTAAAAGCCTGTACACATACTACGATTATTAACGAAGTTAACTG
Consensus                       (1660) GCACATCAAATTCGTATAATTGAGTCGAGCTATCTCTAAAAGCCTGCACACATAATACGATTATTAACGAAGTCAACTG
```

```
                                                                                                   Section 23
                              (1739) 1739      1750      1760      1770      1780      1790      1800             1817
EF495161 HELIANT-g-TMT_ mRNA   (497) --------------------------------------------------------GTTTCATTTCAAGTTGCTGATGC
           DQ229828_F1R92 (1727) TTTTTCTTGGTTATTACGAGCATATAATTCTTGAACTCACGAAGAGGGGTTTGCAGGTTTCATTTCAAGTTGCTGATGC
           DQ229829_F1R92 (1597) TTTTTCATGGTTATTACGAGCATATAATTCTTGAACTCACCATGAGGGGTTTGCAGGTTTCATTTCAAGTTGCTGATGC
           DQ229830_F1R92 (1603) TTTTTCATGGTTATTACGAGCATATAATTCTTGAACTCACCATGAGGGGTTTGCAGGTTTCATTTCAAGTTGCTGATGC
           DQ229831_F1R92 (1725) TTTTTCATGGTTATTACGAGCATATAATTCTTGAACTCACCATGAGGGGTTTGCAGGTTTCATTTCAAGTTGCTGATGC
           DQ229832_F1R92 (1725) TTTTTCATGGTTATTACGAGCATATAATTCTTGAACTCACCATGAGGGGTTTGCAGGTTTCATTTCAAGTTGCTGATGC
        IAST-1_haplotype_4 (1725) TTTTTCATGGTTATTACGAGCATATAATTCTTGAACTCACCATGAGGGGTTTGCAGGTTTCATTTCAAGTTGCTGATGC
      nmsT2100_haplotype_4 (1725) TTTTTCATGGTTATTACGAGCATATAATTCTTGAACTCACCATGAGGGGTTTGCAGGTTTCATTTCAAGTTGCTGATGC
           DQ229833_F1R92 (1719) TTTTTCATGGTTATTACGAGCATATAATTCTTGAATTCACCATGAGGGGTTTGCAGGTTTCATTTCAAGTTGCTGATGC
           DQ229834_F1R92 (1719) TTTTTCATGGTTATTACGAGCATATAATTCTTGAATTCACCATGAGGGGTTTGCAGGTTTCATTTCAAGTTGCTGATGC
        IAST-1_haplotype_5 (1719) TTTTTCATGGTTATTACGAGCATATAATTCTTGAATTCACCATGAGGGGTTTGCAGGTTTCATTTCAAGTTGCTGATGC
      nmsT2100_haplotype_5 (1719) TTTTTCATGGTTATTACGAGCATATAATTCTTGAATTCACCATGAGGGGTTTGCAGGTTTCATTTCAAGTTGCTGATGC
                Consensus (1739) TTTTTCATGGTTATTACGAGCATATAATTCTTGAACTCACCATGAGGGGTTTGCAGGTTTCATTTCAAGTTGCTGATGC
                                                                                                   Section 24
                              (1818) 1818        1830      1840      1850      1860      1870      1880           1896
EF495161 HELIANT-g-TMT_ mRNA   (520) TTTGAACCAGCCGTTTCCTGATGGAAAGTTTGACCTGGTTTGGTCAATGGAGAGTGGAGAGCACATGCCTGACAAACTT
           DQ229828_F1R92 (1806) TTTGAACCAGCCGTTTTCTGATGGAAAGTTTGACCTGGTTTGGTCAATGGAGAGTGGAGAGCACATGCCTGACAAACTT
           DQ229829_F1R92 (1676) TTTAAACCAGCCGTTTCCTGATGGAAAGTTTGACCTTGTTTGGTCAATGGAGAGTGGAGAGCACATGCCTGACAAACTT
           DQ229830_F1R92 (1682) TTTAAACCAGCCGTTTCCTGATGGAAAGTTTGACCTTGTTTGGTCAATGGAGAGTGGAGAGCACATGCCTGACAAACTT
           DQ229831_F1R92 (1804) TTTAAACCAGCCGTTTCCTGATGGAAAGTTTGACCTGGTTTGGTCAATGGAGAGTGGAGAGCACATGCCTGACAAACTT
           DQ229832_F1R92 (1804) TTTAAACCAGCCGTTTCCTGATGGAAAGTTTGACCTGGTTTGGTCAATGGAGAGTGGAGAGCACATGCCTGACAAACTT
        IAST-1_haplotype_4 (1804) TTTAAACCAGCCGTTTCCTGATGGAAAGTTTGACCTGGTTTGGTCAATGGAGAGTGGAGAGCACATGCCTGACAAACTT
      nmsT2100_haplotype_4 (1804) TTTAAACCAGCCGTTTCCTGATGGAAAGTTTGACCTGGTTTGGTCAATGGAGAGTGGAGAGCACATGCCTGACAAACTT
           DQ229833_F1R92 (1798) TTTGAACCAGCCGTTTCCTGATGGAAAGTTTGACCTGGTTTGGTCAATGGAGAGTGGAGAGCACATGCCTGACAAACTT
           DQ229834_F1R92 (1798) TTTGAACCAGCCGTTTCCTGATGGAAAGTTTGACCTGGTTTGGTCAATGGAGAGTGGAGAGCACATGCCTGACAAACTT
        IAST-1_haplotype_5 (1798) TTTGAACCAGCCGTTTCCTGATGGAAAGTTTGACCTGGTTTGGTCAATGGAGAGTGGAGAGCACATGCCTGACAAACTT
      nmsT2100_haplotype_5 (1798) TTTGAACCAGCCGTTTCCTGATGGAAAGTTTGACCTGGTTTGGTCAATGGAGAGTGGAGAGCACATGCCTGACAAACTT
                Consensus (1818) TTT AACCAGCCGTTTCCTGATGGAAAGTTTGACCTGGTTTGGTCAATGGAGAGTGGAGAGCACATGCCTGACAAACTT
```

Section 25

```
                          (1897) 1897         1910      1920      1930      1940      1950      1960          1975
EF495161 HELIANT-g-TMT_ mRNA (599) AAG----------------------------------------------------------------------------
          DQ229828_F1R92 (1885) AAGGTTCTTGTTTTCCTTCACATATTTTAAAATTCTTATCATATCATAGTTGTCAATAGCGATCGC-------GATGGT
          DQ229829_F1R92 (1755) AAGGTTCTTGTTTTCCTTCACATATTTTAAATTTCTTATCATATCATAGTTGTCAATAGCGATCGCCGATCACTATGGT
          DQ229830_F1R92 (1761) AAGGTTCTTGTTTTCCTTCACATATTTTAAATTTCTTATCATATCATAGTTGTCAATAGCGATCGCCGATCACTATGGT
          DQ229831_F1R92 (1883) AAGGTTCTTGTTTTCCTTCACATAT----AATTTCTTATCATATCATAGTTGTCAATAGCGATCGC-------TATGGT
          DQ229832_F1R92 (1883) AAGGTTCTTGTTTTCCTTCACATAT----AATTTCTTATCATATCATAGTTGTCAATAGCGATCGC-------TATGGT
      IAST-1_haplotype_4 (1883) AAGGTTCTTGTTTTCCTTCACATAT----AATTTCTTATCATATCATAGTTGTCAATAGCGATCGC-------TATGGT
    nmsT2100_haplotype_4 (1883) AAGGTTCTTGTTTTCCTTCACATAT----AATTTCTTATCATATCATAGTTGTCAATAGCGATCGC-------TATGGT
          DQ229833_F1R92 (1877) AAGGTTCTTGTTTTCCTTCACATAGTTTAAATTTCTTATCATATCGTAGTTGTCAATAGCGATCGC-------TATGGT
          DQ229834_F1R92 (1877) AAGGTTCTTGTTTTCCTTCACATAGTTTAAATTTCTTATCATATCGTAGTTGTCAATAGCGATCGC-------TATGGT
      IAST-1_haplotype_5 (1877) AAGGTTCTTGTTTTCCTTCACATAGTTTAAATTTCTTATCATATCGTAGTTGTCAATAGCGATCGC-------TATGGT
    nmsT2100_haplotype_5 (1877) AAGGTTCTTGTTTTCCTTCACATAGTTTAAATTTCTTATCATATCGTAGTTGTCAATAGCGATCGC-------TATGGT
               Consensus (1897) AAGGTTCTTGTTTTCCTTCACATATTTTAAATTTCTTATCATATCATAGTTGTCAATAGCGATCGC       TATGGT
```

Section 26

```
                          (1976) 1976          1990      2000      2010      2020      2030      2040          2054
EF495161 HELIANT-g-TMT_ mRNA (602) -------------------------------------------------------------------------------
          DQ229828_F1R92 (1957) CGCTATAGCAAATGGCGTAGCGTATAAGTCGAAGGGAAGGTCGCTACAGGATATCTCGTCATAAATAGCGGGATTTCAG
          DQ229829_F1R92 (1834) CGCTATAGCAAATAGCATAGCGTATAGGTCGAA-----GGTCGCTACAGGATATCTGGCCATAAATAGCGGGATTTCAG
          DQ229830_F1R92 (1840) CGCTATAGCAAATAGCATAGCGTATAGGTCGAA-----GGTCGCTACAGGATATCTGGCCATAAATAGCGGGATTTCAG
          DQ229831_F1R92 (1951) CGCTATAGCAAATAGCGTAGCGTATAGATCGAA-----GGTCGCTACAGGATATCTAGCCATAAATAGCGGGATTTCAG
          DQ229832_F1R92 (1951) CGCTATAGCAAATAGCGTAGCGTATAGATCGAA-----GGTCGCTACAGGATATCTAGCCATAAATAGCGGGATTTCAG
      IAST-1_haplotype_4 (1951) CGCTATAGCAAATAGCGTAGCGTATAGATCGAA-----GGTCGCTACAGGATATCTAGCCATAAATAGCGGGATTTCAG
    nmsT2100_haplotype_4 (1951) CGCTATAGCAAATAGCGTAGCGTATAGATCGAA-----GGTCGCTACAGGATATCTAGCCATAAATAGCGGGATTTCAG
          DQ229833_F1R92 (1949) CGCTATAGCAAATAGCGTAGCGTATAGGTCGAA-----GGTCGCTACAGGATATCTAGCCATAAATAGCGGGATTTCAG
          DQ229834_F1R92 (1949) CGCTATAGCAAATAGCGTAGCGTATAGGTCGAA-----GGTCGCTACAGGATATCTAGCCATAAATAGCGGGATTTCAG
      IAST-1_haplotype_5 (1949) CGCTATAGCAAATAGCGTAGCGTATAGGTCGAA-----GGTCGCTACAGGATATCTAGCCATAAATAGCGGGATTTCAG
    nmsT2100_haplotype_5 (1949) CGCTATAGCAAATAGCGTAGCGTATAGGTCGAA-----GGTCGCTACAGGATATCTAGCCATAAATAGCGGGATTTCAG
               Consensus (1976) CGCTATAGCAAATAGCGTAGCGTATAGGTCGAA     GGTCGCTACAGGATATCTAGCCATAAATAGCGGGATTTCAG
```

```
                                                                                         Section 27
                            (2055) 2055 2060      2070      2080      2090      2100      2110      2120     2133
EF495161 HELIANT-g-TMT_ mRNA (602) -------------------------------------------------------------------------------
           DQ229828_F1R92 (2036) TTTTTTTT-AAATATAGATGGCAATTAAATGTAGATAGCTGTTATATACATGTAAAATAGCGTATATACTAGGGTATTT
           DQ229829_F1R92 (1908) TTTTTTTTTAAATACAGATAGCAATTAAATATAGATAGCGGTTATATACATGTAAAATAGCGTATATACTAGGGTATTT
           DQ229830_F1R92 (1914) TTTTTTTTTAAATACAGATAGCAATTAAATATAGATAGCGGTTATATACATGTAAAATAGCGTATATACTAGGGTATTT
           DQ229831_F1R92 (2025) TTTTTTTT-AAATATAGATAGCAATTAAATATAGATAGCGGTTATATACATGTAAAATAGCTTATATACTAGGGTATTT
           DQ229832_F1R92 (2025) TTTTTTTT-AAATATAGATAGCAATTAAATATAGATAGCGGTTATATACATGTAAAATAGCTTATATACTAGGGTATTT
        IAST-1_haplotype_4 (2025) TTTTTTTT-AAATATAGATAGCAATTAAATATAGATAGCGGTTATATACATGTAAAATAGCTTATATACTAGGGTATTT
      nmsT2100_haplotype_4 (2025) TTTTTTTT-AAATATAGATAGCAATTAAATATAGATAGCGGTTATATACATGTAAAATAGCTTATATACTAGGGTATTT
           DQ229833_F1R92 (2023) TTTTTTTT-AAATATAGATAGCACTTAAATATAGATAGCGGTTATATACATGTAAAATAGCGTATATACTAGGGTATTT
           DQ229834_F1R92 (2023) TTTTTTTT-AAATATAGATAGCACTTAAATATAGATAGCGGTTATATACATGTAAAATAGCGTATATACTAGGGTATTT
        IAST-1_haplotype_5 (2023) TTTTTTTT-AAATATAGATAGCACTTAAATATAGATAGCGGTTATATACATGTAAAATAGCGTATATACTAGGGTATTT
      nmsT2100_haplotype_5 (2023) TTTTTTTT-AAATATAGATAGCACTTAAATATAGATAGCGGTTATATACATGTAAAATAGCGTATATACTAGGGTATTT
                Consensus (2055) TTTTTTTT AAATATAGATAGCAATTAAATATAGATAGCGGTTATATACATGTAAAATAGCGTATATACTAGGGTATTT
                                                                                         Section 28
                            (2134) 2134 2140      2150      2160      2170      2180      2190      2200     2212
EF495161 HELIANT-g-TMT_ mRNA (602) --------------------------------------TTTGTTAGTGAGTTGACTCGGGTGGCTGCCCCCGGAGCTAC
           DQ229828_F1R92 (2114) TGATATAATATTTTAATTTTAATTATTGAATTCACCAGTTTGTTAGTGAGTTGACTCGGGTGGCTGCCCCCGGAGCCAC
           DQ229829_F1R92 (1987) TGATATAATATTTTAATTTTAATTATTGAATTCACCAGTTTGTTAGTGAGTTGACTCGGGTGGCTGCCCCCGGAGCCAC
           DQ229830_F1R92 (1993) TGATATAATATTTTAATTTTAATTATTGAATTCACCAGTTTGTTAGTGAGTTGACTCGGGTGGCTGCCCCCGGAGCCAC
           DQ229831_F1R92 (2103) TGATATAATATTTTAATTTTAATTATTGAATTCACCAGTTTGTTAGTGAGTTGACTCGGGTGGCTGCCCCCGGAGCCAC
           DQ229832_F1R92 (2103) TGATATAATATTTTAATTTTAATTATTGAATTCACCAGTTTGTTAGTGAGTTGACTCGGGTGGCTGCCCCCGGAGCCAC
        IAST-1_haplotype_4 (2103) TGATATAATATTTTAATTTTAATTATTGAATTCACCAGTTTGTTAGTGAGTTGACTCGGGTGGCTGCCCCCGGAGCCAC
      nmsT2100_haplotype_4 (2103) TGATATAATATTTTAATTTTAATTATTGAATTCACCAGTTTGTTAGTGAGTTGACTCGGGTGGCTGCCCCCGGAGCCAC
           DQ229833_F1R92 (2101) TGATATAATATTTTAATTTTAATTATTGAATTCATCAGTTTGTTAGTGAGTTGACTCGGGTGGCTGCCCCCGGAGCTAC
           DQ229834_F1R92 (2101) TGATATAATATTTTAATTTTAATTATTGAATTCATCAGTTTGTTAGTGAGTTGACTCGGGTGGCTGCCCCCGGAGCTAC
        IAST-1_haplotype_5 (2101) TGATATAATATTTTAATTTTAATTATTGAATTCATCAGTTTGTTAGTGAGTTGACTCGGGTGGCTGCCCCCGGAGCTAC
      nmsT2100_haplotype_5 (2101) TGATATAATATTTTAATTTTAATTATTGAATTCATCAGTTTGTTAGTGAGTTGACTCGGGTGGCTGCCCCCGGAGCTAC
                Consensus (2134) TGATATAATATTTTAATTTTAATTATTGAATTCACCAGTTTGTTAGTGAGTTGACTCGGGTGGCTGCCCCCGGAGCCAC
```

```
                                                                                                Section 29
                            (2213) 2213   2220      2230      2240      2250      2260      2270      2280     2291
 EF495161 HELIANT-g-TMT_ mRNA (643) CATTATCATAGTTACATGGTGCCACAGAGATCTTAACCCCGGAGAAAAATCCCTTCGCCCCGAGGAAGAAAAAATCTTG
          DQ229828_F1R92 (2193) CATTATCATAGTTACATGGTGCCACAGAGATCTTAACCCCGGAGAAAAATCCCTTCGCCCCGAGGAAGAAAAAATCTTG
          DQ229829_F1R92 (2066) CATTATCATAGTTACATGGTGCCACAGAGATCTTAACCCCGGAGAAAAATCCCTTCGCCCCGAGGAAGAAAAAATCTTG
          DQ229830_F1R92 (2072) CATTATCATAGTTACATGGTGCCACAGAGATCTTAACCCCGGAGAAAAATCCCTTCGCCCCGAGGAAGAAAAAATCTTG
          DQ229831_F1R92 (2182) CATTATCATAGTTACATGGTGCCACAGAGATCTTAACCCCGGAGAAAAATCCCTTCGCCCCGAGGAAGAAAAAATCTTG
          DQ229832_F1R92 (2182) CATTATCATAGTTACATGGTGCCACAGAGATCTTAACCCCGGAGAAAAATCCCTTCGCCCCGAGGAAGAAAAAATCTTG
     IAST-1_haplotype_4 (2182) CATTATCATAGTTACATGGTGCCACAGAGATCTTAACCCCGGAGAAAAATCCCTTCGCCCCGAGGAAGAAAAAATCTTG
   nmsT2100_haplotype_4 (2182) CATTATCATAGTTACATGGTGCCACAGAGATCTTAACCCCGGAGAAAAATCCCTTCGCCCCGAGGAAGAAAAAATCTTG
          DQ229833_F1R92 (2180) CATTATCATAGTTACATGGTGCCACAGAGATCTTAACCCCGGAGAAAAATCCCTTCGCCCCGAGGAAGAAAAAATCTTG
          DQ229834_F1R92 (2180) CATTATCATAGTTACATGGTGCCACAGAGATCTTAACCCCGGAGAAAAATCCCTTCGCCCCGAGGAAGAAAAAATCTTG
     IAST-1_haplotype_5 (2180) CATTATCATAGTTACATGGTGCCACAGAGATCTTAACCCCGGAGAAAAATCCCTTCGCCCCGAGGAAGAAAAAATCTTG
   nmsT2100_haplotype_5 (2180) CATTATCATAGTTACATGGTGCCACAGAGATCTTAACCCCGGAGAAAAATCCCTTCGCCCCGAGGAAGAAAAAATCTTG
               Consensus (2213) CATTATCATAGTTACATGGTGCCACAGAGATCTTAACCCCGGAGAAAAATCCCTTCGCCCCGAGGAAGAAAAAATCTTG
                                                                                                Section 30
                            (2292) 2292    2300      2310      2320      2330      2340      2350      2360     2370
 EF495161 HELIANT-g-TMT_ mRNA (722) AATAAGATTTGTTCCAGCTTTTATCTTCCTGCTTGGTGTTCTACAGCTGATTATGTAAAGTTACTAGAATCCCTTTCTC
          DQ229828_F1R92 (2272) AATAAGATTTGTTCCAGCTTTTATCTTCCCGCTTGGTGTTCTACAGCTGATTATGTAAAGTTACTAGAATCCCTTTCTC
          DQ229829_F1R92 (2145) AATAAGATTTGTTCCAGCTTTTATCTTCCCGCTTGGTGTTCTACAGCTGATTATGTAAAGTTACTAGAATCCCTTTCTC
          DQ229830_F1R92 (2151) AATAAGATTTGTTCCAGCTTTTATCTTCCCGCTTGGTGTTCTACAGCTGATTATGTAAAGTTACTAGAATCCCTTTCTC
          DQ229831_F1R92 (2261) AATAAGATTTGTTCCAGCTTTTATCTTCCCGCTTGGTGTTCTACAGCTGATTATGTTAAGTTACTAGAATCCCTTTCTC
          DQ229832_F1R92 (2261) AATAAGATTTGTTCCAGCTTTTATCTTCCCGCTTGGTGTTCTACAGCTGATTATGTTAAGTTACTAGAATCCCTTTCTC
     IAST-1_haplotype_4 (2261) AATAAGATTTGTTCCAGCTTTTATCTTCCCGCTTGGTGTTCTACAGCTGATTATGTTAAGTTACTAGAATCCCTTTCTC
   nmsT2100_haplotype_4 (2261) AATAAGATTTGTTCCAGCTTTTATCTTCCCGCTTGGTGTTCTACAGCTGATTATGTTAAGTTACTAGAATCCCTTTCTC
          DQ229833_F1R92 (2259) AATAAGATTTGTTCCAGCTTTTATCTTCCTGCTTGGTGTTCTACAGCTGATTATGTAAAGTTACTAGAATCCCTTTCTC
          DQ229834_F1R92 (2259) AATAAGATTTGTTCCAGCTTTTATCTTCCTGCTTGGTGTTCTACAGCTGATTATGTAAAGTTACTAGAATCCCTTTCTC
     IAST-1_haplotype_5 (2259) AATAAGATTTGTTCCAGCTTTTATCTTCCTGCTTGGTGTTCTACAGCTGATTATGTAAAGTTACTAGAATCCCTTTCTC
   nmsT2100_haplotype_5 (2259) AATAAGATTTGTTCCAGCTTTTATCTTCCTGCTTGGTGTTCTACAGCTGATTATGTAAAGTTACTAGAATCCCTTTCTC
               Consensus (2292) AATAAGATTTGTTCCAGCTTTTATCTTCCCGCTTGGTGTTCTACAGCTGATTATGTAAAGTTACTAGAATCCCTTTCTC
```

Section 31

```
(2371)                           2371     2380      2390      2400      2410      2420      2430             2449
EF495161 HELIANT-g-TMT_ mRNA (801) TTCAG--------------------------------------------------------------------------
DQ229828_F1R92 (2351)            TTCAGGTAAACTTCATTATTAATCGCCCAAAACCTAGTTTAATTTCGGAGTTTATATTAATACTATTTGCAAACATTTA
DQ229829_F1R92 (2224)            TTCAGGTAAACTTCATTATTAATCACCCAAAACCTAGTTTAATTTCGGAGTTTATATTGATA---TTTGCAAACATTTA
DQ229830_F1R92 (2230)            TTCAGGTAAACTTCATTATTAATCACCCAAAACCTAGTTTAATTTCGGAGTTTATATTGATA---TTTGCAAACATTTA
DQ229831_F1R92 (2340)            TTCAGGTAAACTTCATTATTAATCACCCAAAACCTAGTTTAATTTCGGAGTTTATATTAATA---TTTGCAAACATTTA
DQ229832_F1R92 (2340)            TTCAGGTAAACTTCATTATTAATCACCCAAAACCTAGTTTAATTTCGGAGTTTATATTAATA---TTTGCAAACATTTA
IAST-1_haplotype_4 (2340)        TTCAGGTAAACTTCATTATTAATCACCCAAAACCTAGTTTAATTTCGGAGTTTATATTAATA---TTTGCAAACATTTA
nmsT2100_haplotype_4 (2340)      TTCAGGTAAACTTCATTATTAATCACCCAAAACCTAGTTTAATTTCGGAGTTTATATTAATA---TTTGCAAACATTTA
DQ229833_F1R92 (2338)            TTCAGGTAAACTTCATTATTAATCACCCAAAACCTAGTTTAATTTCAGAGTTTATATTAATA---TTTGCAAACATTTA
DQ229834_F1R92 (2338)            TTCAGGTAAACTTCATTATTAATCACCCAAAACCTAGTTTAATTTCAGAGTTTATATTAATA---TTTGCAAACATTTA
IAST-1_haplotype_5 (2338)        TTCAGGTAAACTTCATTATTAATCACCCAAAACCTAGTTTAATTTCAGAGTTTATATTAATA---TTTGCAAACATTTA
nmsT2100_haplotype_5 (2338)      TTCAGGTAAACTTCATTATTAATCACCCAAAACCTAGTTTAATTTCAGAGTTTATATTAATA---TTTGCAAACATTTA
Consensus (2371)                 TTCAGGTAAACTTCATTATTAATCACCCAAAACCTAGTTTAATTTCGGAGTTTATATTAATA   TTTGCAAACATTTA
```

Section 32

```
(2450)                           2450      2460      2470      2480      2490      2500      2510            2528
EF495161 HELIANT-g-TMT_ mRNA (806) -------------------------------------------------------------------------------
DQ229828_F1R92 (2430)            GTTACCTTTAGGAATCTTGAGGGGG-CTACCAACTATCATACACTCCAACCACCTCTCTATCTTTGTCGGCCTGTGAAT
DQ229829_F1R92 (2300)            GTTACCTTTAGGAATCTTGAGGGGGGCTACCAACTATCATACACTCCAACCACCTCTCTATCTTTGTCGGTCTGTGAAT
DQ229830_F1R92 (2306)            GTTACCTTTAGGAATCTTGAGGGGGGCTACCAACTATCATACACTCCAACCACCTCTCTATCTTTGTCGGTCTGTGAAT
DQ229831_F1R92 (2416)            GTTAACTTTAAGAATCTTGAGGGGGGCTACCAACTACCGTACACTCCAACCACCTCTCTATGTCTGTCGGTCTGTGAAT
DQ229832_F1R92 (2416)            GTTAACTTTAAGAATCTTGAGGGGGGCTACCAACTACCGTACACTCCAACCACCTCTCTATGTCTGTCGGTCTGTGAAT
IAST-1_haplotype_4 (2416)        GTTAACTTTAAGAATCTTGAGGGGGGCTACCAACTACCGTACACTCCAACCACCTCTCTATGTCTGTCGGTCTGTGAAT
nmsT2100_haplotype_4 (2416)      GTTAACTTTAAGAATCTTGAGGGGGGCTACCAACTACCGTACACTCCAACCACCTCTCTATGTCTGTCGGTCTGTGAAT
DQ229833_F1R92 (2414)            GTTAACTTTAAGAATCTTGAGGGGGGCTACCAACTACCGTACACTCCAACCACCTCTCTATGTCTGTCGGTCTGTGAAT
DQ229834_F1R92 (2414)            GTTAACTTTAAGAATCTTGAGGGGGGCTACCAACTACCGTACACTCCAACCACCTCTCTATGTCTGTCGGTCTGTGAAT
IAST-1_haplotype_5 (2414)        GTTAACTTTAAGAATCTTGAGGGGGGCTACCAACTACCGTACACTCCAACCACCTCTCTATGTCTGTCGGTCTGTGAAT
nmsT2100_haplotype_5 (2414)      GTTAACTTTAAGAATCTTGAGGGGGGCTACCAACTACCGTACACTCCAACCACCTCTCTATGTCTGTCGGTCTGTGAAT
Consensus (2450)                 GTTAACTTTAAGAATCTTGAGGGGGGCTACCAACTACCGTACACTCCAACCACCTCTCTATGTCTGTCGGTCTGTGAAT
```

```
                                                                                                    Section 33
                           (2529) 2529       2540      2550      2560      2570      2580      2590          2607
EF495161 HELIANT-g-TMT_ mRNA (806) -------------------------------------------------------------------------------
        DQ229828_F1R92 (2508) TGGCAAACTAAAGGGTCGTACGGGTGGGGTAACATATAATAAAGAGGTGGTTCGAAAACAGTTAATTGATTTT-TATGT
        DQ229829_F1R92 (2379) TGGCAAACTAAAGGGTCGTACGGCAGGGTAACAGATAATAAAGAGTTGGTTCGAAAACATTTATTGATTTTT--ATGT
        DQ229830_F1R92 (2385) TGGCAAACTAAAGGGTCGTACGGCAGGGTAACAGATAATAAAGAGTTGGTTCGAAAACATTTATTGATTTTT--ATGT
        DQ229831_F1R92 (2495) TGGCAAACTAAAGGGTCGTACAGGCGGGGTAACGGATAATAAAGAGTTGGTTCAAAAACAGTTATTGATTTTTATGTAT
        DQ229832_F1R92 (2495) TGGCAAACTAAAGGGTCGTACAGGCGGGGTAACGGATAATAAAGAGTTGGTTCAAAAACAGTTATTGATTTTTATGTAT
     IAST-1_haplotype_4 (2495) TGGCAAACTAAAGGGTCGTACAGGCGGGGTAACGGATAATAAAGAGTTGGTTCAAAAACAGTTATTGATTTTTATGTAT
   nmsT2100_haplotype_4 (2495) TGGCAAACTAAAGGGTCGTACAGGCGGGGTAACGGATAATAAAGAGTTGGTTCAAAAACAGTTATTGATTTTTATGTAT
        DQ229833_F1R92 (2493) TGGCAAACTAAAGGGTCGTACAGGCAGGGTAACGGATAATAAAGAGTTGGTTCGAAAACAGTTATTGATTTTT--ATGT
        DQ229834_F1R92 (2493) TGGCAAACTAAAGGGTCGTACAGGCAGGGTAACGGATAATAAAGAGTTGGTTCGAAAACAGTTATTGATTTTT--ATGT
     IAST-1_haplotype_5 (2493) TGGCAAACTAAAGGGTCGTACAGGCAGGGTAACGGATAATAAAGAGTTGGTTCGAAAACAGTTATTGATTTTT--ATGT
   nmsT2100_haplotype_5 (2493) TGGCAAACTAAAGGGTCGTACAGGCAGGGTAACGGATAATAAAGAGTTGGTTCGAAAACAGTTATTGATTTTT--ATGT
              Consensus (2529) TGGCAAACTAAAGGGTCGTACAGGCAGGGTAACGGATAATAAAGAGTTGGTTCGAAAACAGTTATTGATTTTT  ATGT
                                                                                                    Section 34
                           (2608) 2608        2620      2630      2640      2650      2660      2670          2686
EF495161 HELIANT-g-TMT_ mRNA (806) -------------------------------------------------------------------------------
        DQ229828_F1R92 (2586) AGTTAGATTGACCTGAAACACGTTTTGTGTATTTATTTATTTTTTGTAAATAGTTATCGCTTCATCATCATCATACTCA
        DQ229829_F1R92 (2456) AGTTAGATTGACCTGAAACACGTTTTGTTTATTTATTTATTTTTTGTAAATAGTTATCGCTTCATCATCATCATACTCA
        DQ229830_F1R92 (2462) AGTTAGATTGACCTGAAACACGTTTTGTTTATTTATTTATTTTTTGTAAATAGTTATCGCTTCATCATCATCATACTCA
        DQ229831_F1R92 (2574) AGTTAGATTGACCTGAAACACGTTTTGTTTATTTATTTATTTTTTGTAAATAGTTATCGCTTCATCATCATCATACTCG
        DQ229832_F1R92 (2574) AGTTAGATTGACCTGAAACACGTTTTGTTTATTTATTTATTTTTTGTAAATAGTTATCGCTTCATCATCATCATACTCG
     IAST-1_haplotype_4 (2574) AGTTAGATTGACCTGAAACACGTTTTGTTTATTTATTTATTTTTTGTAAATAGTTATCGCTTCATCATCATCATACTCG
   nmsT2100_haplotype_4 (2574) AGTTAGATTGACCTGAAACACGTTTTGTTTATTTATTTATTTTTTGTAAATAGTTATCGCTTCATCATCATCATACTCG
        DQ229833_F1R92 (2570) AGTTCGATTGACCTGAAACACGTTTTGTTTATTTATTTATTTTTTGTAAATAGTTATCGCTTCATCATCATCATACTCA
        DQ229834_F1R92 (2570) AGTTCGATTGACCTGAAACACGTTTTGTTTATTTATTTATTTTTTGTAAATAGTTATCGCTTCATCATCATCATACTCA
     IAST-1_haplotype_5 (2570) AGTTCGATTGACCTGAAACACGTTTTGTTTATTTATTTATTTTTTGTAAATAGTTATCGCTTCATCATCATCATACTCA
   nmsT2100_haplotype_5 (2570) AGTTCGATTGACCTGAAACACGTTTTGTTTATTTATTTATTTTTTGTAAATAGTTATCGCTTCATCATCATCATACTCA
              Consensus (2608) AGTTAGATTGACCTGAAACACGTTTTGTTTATTTATTTATTTTTTGTAAATAGTTATCGCTTCATCATCATCATACTCA
```

Section 35

(2687)	2687 … 2700 … 2710 … 2720 … 2730 … 2740 … 2750 … 2765
EF495161 HELIANT-g-TMT_ mRNA (806)	---
DQ229828_F1R92 (2665)	GTATACACAACCAATAGCAGAGCTAACGTAGGGTATGAGGTGGGTAAGATGTAAATAGCCTTACCTACCCCATAGGAAT
DQ229829_F1R92 (2535)	GTAAACACCACCAATAGCAAAGCTAACGTAGGGTATGAGGGGGGTAAAATGTATATAGCCTTACCTATCCCGTAGGAAT
DQ229830_F1R92 (2541)	GTAAACACCACCAATAGCAAAGCTAACGTAGGGTATGAGGGGGGTAAAATGTAAATAGCCTTACCTATCCCGTAGGAAT
DQ229831_F1R92 (2653)	GTATACACAACCAGTAGCAAAGCTAACGTAGGGTATGAGGAGGGTAAGATGTAAATAGCCTTACCTACCCCGTAGGAAT
DQ229832_F1R92 (2653)	GTATACACAACCAGTAGCAAAGCTAACGTAGGGTATGAGGAGGGTAAGATGTAAATAGCCTTACCTACCCCGTAGGAAT
IAST-1_haplotype_4 (2653)	GTATACACAACCAGTAGCAAAGCTAACGTAGGGTATGAGGAGGGTAAGATGTAAATAGCCTTACCTACCCCGTAGGAAT
nmsT2100_haplotype_4 (2653)	GTATACACAACCAGTAGCAAAGCTAACGTAGGGTATGAGGAGGGTAAGATGTAAATAGCCTTACCTACCCCGTAGGAAT
DQ229833_F1R92 (2649)	ATAAACACCACCAATAGCAAAGCTAACGTAGGGTATGAGGAGGGTAAAATGTAAATAGCCTTACCTACCCCGTAGGAAT
DQ229834_F1R92 (2649)	ATAAACACCACCAATAGCAAAGCTAACGTAGGGTATGAGGAGGGTAAAATGTAAATAGCCTTACCTACCCCGTAGGAAT
IAST-1_haplotype_5 (2649)	ATAAACACCACCAATAGCAAAGCTAACGTAGGGTATGAGGAGGGTAAAATGTAAATAGCCTTACCTACCCCGTAGGAAT
nmsT2100_haplotype_5 (2649)	ATAAACACCACCAATAGCAAAGCTAACGTAGGGTATGAGGAGGGTAAAATGTAAATAGCCTTACCTACCCCGTAGGAAT
Consensus (2687)	GTAAACACCACCAATAGCAAAGCTAACGTAGGGTATGAGGAGGGTAAAATGTAAATAGCCTTACCTACCCCGTAGGAAT

Section 36

(2766)	2766 … 2780 … 2790 … 2800 … 2810 … 2820 … 2830 … 2844
EF495161 HELIANT-g-TMT_ mRNA (806)	---
DQ229828_F1R92 (2744)	AGAAAGACTGCTTCCAGTAAGACCCC-AGCTCGATTCTATTGCTTAAATTATTTAATATGTATTACACATTTTAATTAC
DQ229829_F1R92 (2614)	AGAAAGACTGCTTCCAGT----CCCC-AGCTCGATTCTATTGCTTAAATTATTTAATATGTATTACACATTTTAATTAC
DQ229830_F1R92 (2620)	AGAAAGACTGCTTCCAGT----CCCC-AGCTCGATTCTATTGCTTAAATTATTTAATATGTATTACACATTTTAATTAC
DQ229831_F1R92 (2732)	AGAAAAATTGCTTCCAGTGAGACCCCCAACTCGATTCTATTGCTTAAATTATTTAATATGTATTACACATTTTAATTAC
DQ229832_F1R92 (2732)	AGAAAAATTGCTTCCAGTGAGACCCCCAACTCGATTCTATTGCTTAAATTATTTAATATGTATTACACATTTTAATTAC
IAST-1_haplotype_4 (2732)	AGAAAAATTGCTTCCAGTGAGACCCCCAACTCGATTCTATTGCTTAAATTATTTAATATGTATTACACATTTTAATTAC
nmsT2100_haplotype_4 (2732)	AGAAAAATTGCTTCCAGTGAGACCCCCAACTCGATTCTATTGCTTAAATTATTTAATATGTATTACACATTTTAATTAC
DQ229833_F1R92 (2728)	AGAAAGACTGCTTCCAGTGAGACCCC-AGCTTGATTTTATTGCTTAAATTATTTAATATGTATTACACATTTTAGTTAC
DQ229834_F1R92 (2728)	AGAAAGACTGCTTCCAGTGAGACCCC-AGCTTGATTTTATTGCTTAAATTATTTAATATGTATTACACATTTTAGTTAC
IAST-1_haplotype_5 (2728)	AGAAAGACTGCTTCCAGTGAGACCCC-AGCTCGATTCTATTGCTTAAATTATTTAATATGTATTACACATTTTAGTTAC
nmsT2100_haplotype_5 (2728)	AGAAAGACTGCTTCCAGTGAGACCCC-AGCTCGATTCTATTGCTTAAATTATTTAATATGTATTACACATTTTAGTTAC
Consensus (2766)	AGAAAGACTGCTTCCAGTGAGACCCC AGCTCGATTCTATTGCTTAAATTATTTAATATGTATTACACATTTTAATTAC

Section 37

```
                          (2845) 2845 2850      2860      2870      2880      2890      2900      2910     2923
EF495161 HELIANT-g-TMT_ mRNA (806) -------------------------------------------------------------------------------
          DQ229828_F1R92 (2822) GTGTTAGGAGACTTTTGACCCGTTATCATTTAAGCTAT----------------------TTTTTTTTTTAATTCGACT
          DQ229829_F1R92 (2688) ATGTTAAGAGACTTTTGACCCGTTATCATTTAAGCTAT----------------------TTTTTTTT-CAATTTGACT
          DQ229830_F1R92 (2694) ATGTTAAGAGACTTTTGACCCGTTATCATTTAAGCTAT----------------------TTTTTTTT-CAATTTGACT
          DQ229831_F1R92 (2811) GTGTTAAGAGACTTTTGACCCGTTATCATTTAAGCTAT----------------------TTTTTTT--CAATTTGACT
          DQ229832_F1R92 (2811) GTGTTAAGAGACTTTTGACCCGTTATCATTTAAGCTAT----------------------TTTTTTT--CAATTTGACT
       IAST-1_haplotype_4 (2811) GTGTTAAGAGACTTTTGACCCGTTATCATTTAAGCTAT------------------------TTTTTTTCAATTTGACT
      nmsT2100_haplotype_4 (2811) GTGTTAAGAGACTTTTGACCCGTTATCATTTAAGCTAT------------------------TTTTTTTCAATTTGACT
          DQ229833_F1R92 (2806) GTGTTAGGAGACTTTTGACCCGTTATCATTTAAAAAAAAAAAAAAAA--TATATATATATTTTTTTTTTAATTTGACT
          DQ229834_F1R92 (2806) GTGTTAGGAGACTTTTGACCCGTTATCATTTAAAAAAAAAAAAAAAA--TATATATATATTTTTTTTTTAATTTGACT
       IAST-1_haplotype_5 (2806) GTGTTAGGAGACTTTTGACCCGTTATCATTTAAAAAAAAAAAAAAAAA-TATATATATATTTTTTTTTTAATTTGACT
      nmsT2100_haplotype_5 (2806) GTGTTAGGAGACTTTTGACCCGTTATCATTTAAAAAAAAAAATATATATATATATATATATTTTTTTTTTAATTTGACT
               Consensus (2845) GTGTTAAGAGACTTTTGACCCGTTATCATTTAAGCTAT                      TTTTTTTTTCAATTTGACT
```

Section 38

```
                          (2924) 2924  2930      2940      2950      2960      2970      2980      2990     3002
EF495161 HELIANT-g-TMT_ mRNA (806) -------------------------------------------------------------------------------
          DQ229828_F1R92 (2879) CATTTAATATTAAGATAGACCCTGAACCAAATAATTTATAAGTAAATTCCATTTACACCTCACGTTA------------
          DQ229829_F1R92 (2744) CATTTAATACTAAGATAGACCCTGAACCAAATAATGTATAAGTAAATTCCATTTACACCTCACGTTA------------
          DQ229830_F1R92 (2750) CATTTAATACTAAGATAGACCCTGAACCAAATAATGTATAAGTAAATTCCATTTACACCTCACGTTA------------
          DQ229831_F1R92 (2866) CATTTAATACTAAGATAGACCCTGAACCAAATAATGTATAAGTAAATTCCATTTACACCTCACGTTA------------
          DQ229832_F1R92 (2866) CATTTAATACTAAGATAGACCCTGAACCAAATAATGTATAAGTAAATTCCATTTACACCTCACGTTA------------
       IAST-1_haplotype_4 (2866) CATTTAATACTAAGATAGACCCTGAACCAAATAATGTATAAGTAAATTCCATTTACACCTCACGTTA------------
      nmsT2100_haplotype_4 (2866) CATTTAATACTAAGATAGACCCTGAACCAAATAATGTATAAGTAAATTCCATTTACACCTCACGTTA------------
          DQ229833_F1R92 (2883) CATTTAATATTAAGATAGACCCTGAACCAAATAATGTATAAGTAAATTCCATTTACACCTCACGTTATAAATAGCTTGA
          DQ229834_F1R92 (2883) CATTTAATATTAAGATAGACCCTGAACCAAATAATGTATAAGTAAATTCCATTTACACCTCACGTTATAAATAGCTTGA
       IAST-1_haplotype_5 (2884) CATTTAATATTAAGATAGACCCTGAACCAAATAATGTATAAGTAAATTCCATTTACACCTCACGTTATAAATAGCTTGA
      nmsT2100_haplotype_5 (2885) CATTTAATATTAAGATAGACCCTGAACCAAATAATGTATAAGTAAATTCCATTTACACCTCACGTTATAAATAGCTTGA
               Consensus (2924) CATTTAATACTAAGATAGACCCTGAACCAAATAATGTATAAGTAAATTCCATTTACACCTCACGTTA
```

```
                                                                                                  Section 39
                              (3003) 3003   3010      3020      3030      3040      3050      3060      3070     3081
EF495161 HELIANT-g-TMT_ mRNA   (806) ---------------GACATAAAATCCGCAGACTGGTCTGGCAATGTGGCCCCGTTTTGGCCTGCTGTAATAAAAACAG
DQ229828_F1R92                (2946) -----CACTTTGCAGGACATAAAATCCGCAGACTGGTCTGGCAATGTGGCCCCATTTTGGCCTGCTGTAATAAAAACAG
DQ229829_F1R92                (2811) -----CACTTTGCAGGACATAAAATCCGCAGACTGGTCTGGCAATGTGGCCCCATTTTGGCCTGCTGTAATAAAAACAG
DQ229830_F1R92                (2817) -----CACTTTGCAGGACATAAAATCCGCAGACTGGTCTGGCAATGTGGCCCCATTTTGGCCTGCTGTAATAAAAACAG
DQ229831_F1R92                (2933) -----CACTTTGCAGGACATAAAATCCGCAGACTGGTCTGGCAATGTGGCCCCATTTTGGCCTGCTGTAATAAAAACAG
DQ229832_F1R92                (2933) -----CACTTTGCAGGACATAAAATCCGCAGACTGGTCTGGCAATGTGGCCCCATTTTGGCCTGCTGTAATAAAAACAG
IAST-1_haplotype_4            (2933) -----CACTTTGCAGGACATAAAATCCGCAGACTGGTCTGGCAATGTGGCCCCATTTTGGCCTGCTGTAATAAAAACAG
nmsT2100_haplotype_4          (2933) -----CACTTTGCAGGACATAAAATCCGCAGACTGGTCTGGCAATGTGGCCCCATTTTGGCCTGCTGTAATAAAAACAG
DQ229833_F1R92                (2962) AGTGTCACTTTGCAGGACATAAAATCCGCAGACTGGTCTGGCAATGTGGCCCCGTTTTGGCCTGCTGTAATAAAAACAG
DQ229834_F1R92                (2962) AGTGTCACTTTGCAGGACATAAAATCCGCAGACTGGTCTGGCAATGTGGCCCCGTTTTGGCCTGCTGTAATAAAAACAG
IAST-1_haplotype_5            (2963) AGTGTCACTTTGCAGGACATAAAATCCGCAGACTGGTCTGGCAATGTGGCCCCGTTTTGGCCTGCTGTAATAAAAACAG
nmsT2100_haplotype_5          (2964) AGTGTCACTTTGCAGGACATAAAATCCGCAGACTGGTCTGGCAATGTGGCCCCGTTTTGGCCTGCTGTAATAAAAACAG
Consensus                     (3003)      CACTTTGCAGGACATAAAATCCGCAGACTGGTCTGGCAATGTGGCCCCATTTTGGCCTGCTGTAATAAAAACAG
                                                                                                  Section 40
                              (3082) 3082    3090      3100      3110      3120      3130      3140      3150     3160
EF495161 HELIANT-g-TMT_ mRNA   (870) CATTGTCTTGGAAGGGCATTACTTCATTGCTACGTAGTG----------------------------------------
DQ229828_F1R92                (3020) CGTTGTCTTGGAAGGGCATTACTTCATTGCTACGTAGTGGTAATGCAAACTTTTTTACTTAAAGACACACACACACA
DQ229829_F1R92                (2885) CATTGTCTTGGAAGGGCATTACTTCATTGCTACGTAGTGGTAATGCAAACTTTTTTACTTAAACACACACACACACACA
DQ229830_F1R92                (2891) CATTGTCTTGGAAGGGCATTACTTCATTGCTACGTAGTGGTAATGCAAACTTTTTTACTTAAACACACACACACACACA
DQ229831_F1R92                (3007) CATTGTCTTGGAAGGGCATTACTTCATTGCTACGTAGTGGTAATGCAAACTTTTTTACTTAAACACACACACACACACA
DQ229832_F1R92                (3007) CATTGTCTTGGAAGGGCATTACTTCATTGCTACGTAGTGGTAATGCAAACTTTTTTACTTAAACACACACACACACACA
IAST-1_haplotype_4            (3007) CATTGTCTTGGAAGGGCATTACTTCATTGCTACGTAGTGGTAATGCAAACTTTTTTACTTAAACACACACACACACACA
nmsT2100_haplotype_4          (3007) CATTGTCTTGGAAGGGCATTACTTCATTGCTACGTAGTGGTAATGCAAACTTTTTTACTTAAACACACACACACACACA
DQ229833_F1R92                (3041) CATTGTCTTGGAAGGGCATTACTTCATTGCTACGTAGTGGTAATGCAAACTTTTTTACTTAAACACACACACA------
DQ229834_F1R92                (3041) CATTGTCTTGGAAGGGCATTACTTCATTGCTACGTAGTGGTAATGCAAACTTTTTTACTTAAACACACACACA------
IAST-1_haplotype_5            (3042) CATTGTCTTGGAAGGGCATTACTTCATTGCTACGTAGTGGTAATGCAAACTTTTTTACTTAAACACACACACA------
nmsT2100_haplotype_5          (3043) CATTGTCTTGGAAGGGCATTACTTCATTGCTACGTAGTGGTAATGCAAACTTTTTTACTTAAACACACACACA------
Consensus                     (3082) CATTGTCTTGGAAGGGCATTACTTCATTGCTACGTAGTGGTAATGCAAACTTTTTTACTTAAACACACACACACACACA
```

```
                                                                                                   Section 41
                           (3161) 3161     3170      3180      3190      3200      3210      3220          3239
EF495161 HELIANT-g-TMT_ mRNA (909) -------------------------------------------------------------------------------
         DQ229828_F1R92 (3099) CATATATATATGGTATTAGGATCAAATACAAATAGGTTTTACTGTAGAAGCGTACACACCAAAGAGAAATAACACGCGG
         DQ229829_F1R92 (2964) CATATA----------------------------GTATTACTGTAAGAAGCTTACACACCAAAGAGAAGTAACACGCGG
         DQ229830_F1R92 (2970) CATATA----------------------------GTATTACTGTAAGAAGCTTACACACCAAAGAGAAGTAACACGCGG
         DQ229831_F1R92 (3086) CACATATA--------------------------GTATTACTGTAAGAAGCTTACACACCAAAGAGAAGTAACACGCGG
         DQ229832_F1R92 (3086) CACATATA--------------------------GTATTACTGTAAGAAGCTTACACACCAAAGAGAAGTAACACGCGG
     IAST-1_haplotype_4 (3086) CACATATA--------------------------GTATTACTGTAAGAAGCTTACACACCAAAGAGAAGTAACACGCGG
   nmsT2100_haplotype_4 (3086) CACATATA--------------------------GTATTACTGTAAGAAGCTTACACACCAAAGAGAAGTAACACGCGG
         DQ229833_F1R92 (3114) --TATATA---GGATTAGGATCAACTACAAATAGGTTTTACTGTAAGAAGCGTACACACCAAAGAGAAATAACACGCGG
         DQ229834_F1R92 (3114) --TATATA---GGATTAGGATCAACTACAAATAGGTTTTACTGTAAGAAGCGTACACACCAAAGAGAAATAACACGCGG
     IAST-1_haplotype_5 (3115) --TATATA---GGATTAGGATCAACTACAAATAGGTTTTACTGTAAGAAGCGTACACACCAAAGAGAAATAACACGCGG
   nmsT2100_haplotype_5 (3116) --TATATA---GGATTAGGATCAACTACAAATAGGTTTTACTGTAAGAAGCGTACACACCAAAGAGAAATAACACGCGG
              Consensus (3161) CATATATA                          GTATTACTGTAAGAAGCTTACACACCAAAGAGAAGTAACACGCGG
                                                                                                   Section 42
                           (3240) 3240      3250      3260      3270      3280      3290      3300          3318
EF495161 HELIANT-g-TMT_ mRNA (909) -------------------------------------------------------------------------------
         DQ229828_F1R92 (3178) TGACATTTTTCTAAATAGTGTAATTACACTACAACAAAATTAAACTAGAGTAATTATTATTACGCCATAGTTTTCAAGT
         DQ229829_F1R92 (3015) TGACATTTCTCTAAATAGTGTAATTACACTACAACAAAATTAAACTAGAGTAATCATTATTACGC--------------
         DQ229830_F1R92 (3021) TGACATTTCTCTAAATAGTGTAATTACACTACAACAAAATTAAACTAGAGTAATCATTATTACGC--------------
         DQ229831_F1R92 (3139) TGACATTTTTCTAAATAGTGTAATTACACTACAACAAAATTAAACTAGAGTAATCATTATTACGC--------------
         DQ229832_F1R92 (3139) TGACATTTTTCTAAATAGTGTAATTACACTACAACAAAATTAAACTAGAGTAATCATTATTACGC--------------
     IAST-1_haplotype_4 (3139) TGACATTTTTCTAAATAGTGTAATTACACTACAACAAAATTAAACTAGAGTAATCATTATTACGC--------------
   nmsT2100_haplotype_4 (3139) TGACATTTTTCTAAATAGTGTAATTACACTACAACAAAATTAAACTAGAGTAATCATTATTACGC--------------
         DQ229833_F1R92 (3188) TGACATTTTTCTAAATAGTGTAATTACACTACAACAAAATTAAACTAGAGTAATTATTATTACGCCATAGTTTTCAAGT
         DQ229834_F1R92 (3188) TGACATTTTTCTAAATAGTGTAATTACACTACAACAAAATTAAACTAGAGTAATTATTATTACGCCATAGTTTTCAAGT
     IAST-1_haplotype_5 (3189) TGACATTTTTCTAAATAGTGTAATTACACTACAACAAAATTAAACTAGAGTAATTATTATTACGCCATAGTTTTCAAGT
   nmsT2100_haplotype_5 (3190) TGACATTTTTCTAAATAGTGTAATTACACTACAACAAAATTAAACTAGAGTAATTATTATTACGCCATAGTTTTCAAGT
              Consensus (3240) TGACATTTTTCTAAATAGTGTAATTACACTACAACAAAATTAAACTAGAGTAATCATTATTACGC
```

Section 43

```
                         (3319) 3319       3330      3340      3350      3360      3370      3380            3397
EF495161 HELIANT-g-TMT_ mRNA (909) -------------------------------------------------------------------------------
         DQ229828_F1R92 (3257) AGCGTAACGATAGATATGCTACTTCATTTGTTGTATTGTATCACACGCTACAAGAAAATTGAACTAGAGTAATTATTAT
         DQ229829_F1R92 (3080) ------------------------------------------------TACTTGAAAACTATGACGCAATAATAATTAC
         DQ229830_F1R92 (3086) ------------------------------------------------TACTTGAAAACTATGACGCAATAATAATTAC
         DQ229831_F1R92 (3204) ------------------------------------------------TACTTGAAAACTATGACGCAATAATAATTAC
         DQ229832_F1R92 (3204) ------------------------------------------------TACTTGAAAACTATGACGCAATAATAATTAC
      IAST-1_haplotype_4 (3204) ------------------------------------------------TACTTGAAAACTATGACGCAATAATAATTAC
    nmsT2100_haplotype_4 (3204) ------------------------------------------------TACTTGAAAACTATGACGCAATAATAATTAC
         DQ229833_F1R92 (3267) AGCGTAATGATAGATATGCTACTTCATTTGTTGTAGTGTATCATACGCTACAACAAAATTGAACTAGAGTAATTATTAT
         DQ229834_F1R92 (3267) AGCGTAATGATAGATATGCTACTTCATTTGTTGTAGTGTATCATACGCTACAACAAAATTGAACTAGAGTAATTATTAT
      IAST-1_haplotype_5 (3268) AGCGTAATGATAGATATGCTACTTCATTTGTTGTAGTGTATCATACGCTACAACAAAATTGAACTAGAGTAATTATTAT
    nmsT2100_haplotype_5 (3269) AGCGTAATGATAGATATGCTACTTCATTTGTTGTAGTGTATCATACGCTACAACAAAATTGAACTAGAGTAATTATTAT
              Consensus (3319)                                                 TACTTGAAAACTATGACGCAATAATAATTAC
```

Section 44

```
                         (3398) 3398        3410      3420      3430      3440      3450      3460          3476
EF495161 HELIANT-g-TMT_ mRNA (909) -------------------------------------------------------------------------------
         DQ229828_F1R92 (3336) TACGCTACTTGAAAACTATGACGCAATAATAATTACTCTAGTTCAATTTTTAGCAGCGTAATTTCTTAGCACGCTAAAG
         DQ229829_F1R92 (3111) T----------------------------CTAGTAGTCTAGTTCAATTTTTAGCAGCGTGATTTCTCAGCACGCTAAAG
         DQ229830_F1R92 (3117) T----------------------------CTAGTAGTCTAGTTCAATTTTTAGCAGCGTGATTTCTCAGCACGCTAAAG
         DQ229831_F1R92 (3235) T----------------------------CTAGTAGTCTAGTTCAATTTTTAGCAGCGTAATTTCTCAGCACGCTAAAG
         DQ229832_F1R92 (3235) T----------------------------CTAGTAGTCTAGTTCAATTTTTAGCAGCGTAATTTCTCAGCACGCTAAAG
      IAST-1_haplotype_4 (3235) T----------------------------CTAGTAGTCTAGTTCAATTTTTAGCAGCGTAATTTCTCAGCACGCTAAAG
    nmsT2100_haplotype_4 (3235) T----------------------------CTAGTAGTCTAGTTCAATTTTTAGCAGCGTAATTTCTCAGCACGCTAAAG
         DQ229833_F1R92 (3346) TACGCTACTTGAAAACTATGACGCAATAATAATTACTCTAGCTCAATTTTTAGCAGCGTAATTTCTTAGCACGCTAAAG
         DQ229834_F1R92 (3346) TACGCTACTTGAAAACTATGACGCAATAATAATTACTCTAGCTCAATTTTTAGCAGCGTAATTTCTTAGCACGCTAAAG
      IAST-1_haplotype_5 (3347) TACGCTACTTGAAAACTATGACGCAATAATAATTACTCTAGCTCAATTTTTAGCAGCGTAATTTCTTAGCACGCTAAAG
    nmsT2100_haplotype_5 (3348) TACGCTACTTGAAAACTATGACGCAATAATAATTACTCTAGCTCAATTTTTAGCAGCGTAATTTCTTAGCACGCTAAAG
              Consensus (3398) T                            CTAGTAGTCTAGTTCAATTTTTAGCAGCGTAATTTCTCAGCACGCTAAAG
```

```
                                                                                     Section 45
                           (3477) 3477         3490      3500      3510      3520      3530      3540           3555
 EF495161 HELIANT-g-TMT_ mRNA (909) -------------------------------------------------------------------------------
           DQ229828_F1R92 (3415) TTTTGTTTGAAAAAAAT---TTTCACCCCTACATATATACACACACACACACACACACGCACACACACAATTCTAAGTA
           DQ229829_F1R92 (3162) TTTTGTTTGAAAAAAAAA--TTTCATCCCTACATATACACACACACACGCGCGCGCGCGCACACACACAATTCTAAGTA
           DQ229830_F1R92 (3168) TTTTGTTTGAAAAAAAAA--TTTCATCCCTACATATACACACACACACGCGCGCGCGCGCACACACACAATTCTAAGTA
           DQ229831_F1R92 (3286) TTTTGTTTGAAAAAAAAA--TTTCATCCCTACATATACACACACAC--GCGCGCGCGCACACACACACAATTCTAAGTA
           DQ229832_F1R92 (3286) TTTTGTTTGAAAAAAAAA--TTTCATCCCTACATATACACACACAC--GCGCGCGCGCACACACACACAATTCTAAGTA
      IAST-1_haplotype_4 (3286) TTTTGTTTGAAAAAAAAA--TTTCATCCCTACATATACACACACAC--GCGCGCGCGCACACACACACAATTCTAAGTA
    nmsT2100_haplotype_4 (3286) TTTTGTTTGAAAAAAAAA--TTTCATCCCTACATATACACACACAC--GCGCGCGCGCACACACACACAATTCTAAGTA
           DQ229833_F1R92 (3425) TTTTGTTTGAAAAAAAAAAATT-CACCCCTACATATATACACACAC------------------ACACAATTCTAAGTA
           DQ229834_F1R92 (3425) TTTTGTTTGAAAAAAAAAAATT-CACCCCTACATATATACACACAC------------------ACACAATTCTAAGTA
      IAST-1_haplotype_5 (3426) TTTTGTTTGAAAAAAAAAAATT-CACCCCTACATATATACACACAC------------------ACACAATTCTAAGTA
    nmsT2100_haplotype_5 (3427) TTTTGTTTGAAAAAAAAAAATT-CACCCCTACATATATACACACAC------------------ACACAATTCTAAGTA
                Consensus (3477) TTTTGTTTGAAAAAAAAA  TTTCATCCCTACATATACACACACAC  GCGCGCGCGC CACACACACAATTCTAAGTA
                                                                                     Section 46
                           (3556) 3556           3570      3580      3590      3600      3610      3620          3634
 EF495161 HELIANT-g-TMT_ mRNA (909) -------------------------------------------------------------------------------
           DQ229828_F1R92 (3491) GAGAATTGTGAGAACTAATATAATCACATTTACTTATCCAAGACATAAGTTAATCGTGTAACTGCCTCACTTTCTCTCG
           DQ229829_F1R92 (3239) GAGAATTGTGAGAACTAATATAATCACGTTTACTTATCTAAGACATAAGTTAATTGTGTAACTGCCTCACTTTCTCTCG
           DQ229830_F1R92 (3245) GAGAATTGTGAGAACTAATATAATCACGTTTACTTATCTAAGACATAAGTTAATTGTGTAACTGCCTCACTTTCTCTCG
           DQ229831_F1R92 (3361) GAGAATTGTGAGAACTAATATAATCACGTTTACTTATCTAAGACATAAGTTAATTGTGTAACTGCCTCACTTTCTCTCG
           DQ229832_F1R92 (3361) GAGAATTGTGAGAACTAATATAATCACGTTTACTTATCTAAGACATAAGTTAATTGTGTAACTGCCTCACTTTCTCTCG
      IAST-1_haplotype_4 (3361) GAGAATTGTGAGAACTAATATAATCACGTTTACTTATCTAAGACATAAGTTAATTGTGTAACTGCCTCACTTTCTCTCG
    nmsT2100_haplotype_4 (3361) GAGAATTGTGAGAACTAATATAATCACGTTTACTTATCTAAGACATAAGTTAATTGTGTAACTGCCTCACTTTCTCTCG
           DQ229833_F1R92 (3485) GAGAATTGTGAGAACTAATATAATCACGTTTACTTATCTAAGACATAAGTTAATCGTGTAACTGCCTCACTTTCTCTCG
           DQ229834_F1R92 (3485) GAGAATTGTGAGAACTAATATAATCACGTTTACTTATCTAAGACATAAGTTAATCGTGTAACTGCCTCACTTTCTCTCG
      IAST-1_haplotype_5 (3486) GAGAATTGTGAGAACTAATATAATCACGTTTACTTATCTAAGACATAAGTTAATCGTGTAACTGCCTCACTTTCTCTCG
    nmsT2100_haplotype_5 (3487) GAGAATTGTGAGAACTAATATAATCACGTTTACTTATCTAAGACATAAGTTAATCGTGTAACTGCCTCACTTTCTCTCG
                Consensus (3556) GAGAATTGTGAGAACTAATATAATCACGTTTACTTATCTAAGACATAAGTTAATTGTGTAACTGCCTCACTTTCTCTCG
```

```
                                                                                                     Section 47
                              (3635) 3635 3640      3650      3660      3670      3680      3690      3700        3713
EF495161 HELIANT-g-TMT_ mRNA  (909)  -------------------------------------------------------------------------------
         DQ229828_F1R92 (3570)       TCCACTAAATTTGACATCCACAACATACAATTATTTAAGTATAAATTGAGGTTCCCGACTTAATAATTGTTATGTATTA
         DQ229829_F1R92 (3318)       TCCACTAAATTTGACATCCACAACATACAATTGTTT----------TAAG--------------------TATATTTTG
         DQ229830_F1R92 (3324)       TCCACTAAATTTGACATCCACAACATACAATTGTTT----------TAAG--------------------TATATTTTG
         DQ229831_F1R92 (3440)       TCCACTAAATTTGACATCCACAACATACAATTGTTT----------TAAG--------------------TATATTTTG
         DQ229832_F1R92 (3440)       TCCACTAAATTTGACATCCACAACATACAATTGTTT----------TAAG--------------------TATATTTTG
     IAST-1_haplotype_4 (3440)       TCCACTAAATTTGACATCCACAACATACAATTGTTT----------TAAG--------------------TATATTTTG
   nmsT2100_haplotype_4 (3440)       TCCACTAAATTTGACATCCACAACATACAATTGTTT----------TAAG--------------------TATATTTTG
         DQ229833_F1R92 (3564)       TCCACTAAATTTGACATCCACAACATACAATTATTTAAGTATAAATTGAGGTTCCCGACTTAATAATTGTTATGTATTA
         DQ229834_F1R92 (3564)       TCCACTAAATTTGACATCCACAACATACAATTATTTAAGTATAAATTGAGGTTCCCGACTTAATAATTGTTATGTATTA
     IAST-1_haplotype_5 (3565)       TCCACTAAATTTGACATCCACAACATACAATTATTTAAGTATAAATTGAGGTTCCCGACTTAATAATTGTTATGTATTA
   nmsT2100_haplotype_5 (3566)       TCCACTAAATTTGACATCCACAACATACAATTATTTAAGTATAAATTGAGGTTCCCGACTTAATAATTGTTATGTATTA
              Consensus (3635)       TCCACTAAATTTGACATCCACAACATACAATTGTTT          TAAG                    TATATTTTG

                                                                                                     Section 48
                              (3714) 3714  3720      3730      3740      3750      3760      3770      3780       3792
EF495161 HELIANT-g-TMT_ mRNA  (909)  -------------------------------------------------------------------------------
         DQ229828_F1R92 (3649)       AACGCGTCCATATACATGTAAATAGAGAGATAGGGCGCGCTCAGTATACTATCAATTTTAAGGAGAAAACTACAACAAT
         DQ229829_F1R92 (3367)       AACGTGTCCATATACATGTAAATAGAGAGATAGGGCGCGCTCAGTATACTATCAATTTTAAGGAGAAAACTACAAGAAT
         DQ229830_F1R92 (3373)       AACGTGTCCATATACATGTAAATAGAGAGATAGGGCGCGCTCAGTATACTATCAATTTTAAGGAGAAAACTACAAGAAT
         DQ229831_F1R92 (3489)       AACGCGTCCATATACATGTAAATAGAGAGATAGGGCGCGCTGAGTATACTATCAATTTTAAGGAGAAAACTACAAGAAT
         DQ229832_F1R92 (3489)       AACGCGTCCATATACATGTAAATAGAGAGATAGGGCGCGCTGAGTATACTATCAATTTTAAGGAGAAAACTACAAGAAT
     IAST-1_haplotype_4 (3489)       AACGCGTCCATATACATGTAAATAGAGAGATAGGGCGCGCTGAGTATACTATCAATTTTAAGGAGAAAACTACAAGAAT
   nmsT2100_haplotype_4 (3489)       AACGCGTCCATATACATGTAAATAGAGAGATAGGGCGCGCTGAGTATACTATCAATTTTAAGGAGAAAACTACAAGAAT
         DQ229833_F1R92 (3643)       AACGCGTCCATATACATGTAAATAGAGAGATAGGGCACGCTCAGTATACTATCAATTTTAAGGAGAAAACTACAAGAAT
         DQ229834_F1R92 (3643)       AACGCGTCCATATACATGTAAATAGAGAGATAGGGCACGCTCAGTATACTATCAATTTTAAGGAGAAAACTACAAGAAT
     IAST-1_haplotype_5 (3644)       AACGCGTCCATATACATGTAAATAGAGAGATAGGGCACGCTCAGTATACTATCAATTTTAAGGAGAAAACTACAAGAAT
   nmsT2100_haplotype_5 (3645)       AACGCGTCCATATACATGTAAATAGAGAGATAGGGCACGCTCAGTATACTATCAATTTTAAGGAGAAAACTACAAGAAT
              Consensus (3714)       AACGCGTCCATATACATGTAAATAGAGAGATAGGGCGCGCTCAGTATACTATCAATTTTAAGGAGAAAACTACAAGAAT
```

```
                                                                                                        Section 49
                                 (3793) 3793   3800      3810      3820      3830      3840      3850      3860    3871
EF495161 HELIANT-g-TMT_ mRNA     (909)  -------------------------------------------------------------------------------
DQ229828_F1R92                   (3728) CATATAAACATTTTTACGTTGTGGTTATGTTATAAAGTGGAAGAGAGAGATAGTGGAGCGGTT-ATGCAGTTAGATAGC
DQ229829_F1R92                   (3446) CATATAAACATTTTTACGTTGTGGTTATCTTATAAAGTGGAAGAGAGAGATAGTGGAGCGGTT-ATGCGGTTAGATAGC
DQ229830_F1R92                   (3452) CATATAAACATTTTTACGTTGTGGTTATCTTATAAAGTGGAAGAGAGAGATAGTGGAGCGGTT-ATGCGGTTAGATAGC
DQ229831_F1R92                   (3568) CATATAAACATTTTTACGTTGTGGTTATCTTATAAAGTGGAAGAGAGAGATAGTGGAGCGGTTTATGCGGTTAGATAGC
DQ229832_F1R92                   (3568) CATATAAACATTTTTACGTTGTGGTTATCTTATAAAGTGGAAGAGAGAGATAGTGGAGCGGTTTATGCGGTTAGATAGC
IAST-1_haplotype_4               (3568) CATATAAACATTTTTACGTTGTGGTTATCTTATAAAGTGGAAGAGAGAGATAGTGGAGCGGTTTATGCGGTTAGATAGC
nmsT2100_haplotype_4             (3568) CATATAAACATTTTTACGTTGTGGTTATCTTATAAAGTGGAAGAGAGAGATAGTGGAGCGGTTTATGCGGTTAGATAGC
DQ229833_F1R92                   (3722) CATATAAACATTTTTACGTTGTGGTTATGTTATAAAGTGGAAGAGAGAGATAGTGGAGCGGTT-ATGCGGTTAGATAGC
DQ229834_F1R92                   (3722) CATATAAACATTTTTACGTTGTGGTTATGTTATAAAGTGGAAGAGAGAGATAGTGGAGCGGTT-ATGCGGTTAGATAGC
IAST-1_haplotype_5               (3723) CATATAAACATTTTTACGTTGTGGTTATGTTATAAAGTGGAAGAGAGAGATAGTGGAGCGGTT-ATGCGGTTAGATAGC
nmsT2100_haplotype_5             (3724) CATATAAACATTTTTACGTTGTGGTTATGTTATAAAGTGGAAGAGAGAGATAGTGGAGCGGTT-ATGCGGTTAGATAGC
Consensus                        (3793) CATATAAACATTTTTACGTTGTGGTTATCTTATAAAGTGGAAGAGAGAGATAGTGGAGCGGTT ATGCGGTTAGATAGC
                                                                                                        Section 50
                                 (3872) 3872    3880      3890      3900      3910      3920      3930      3940    3950
EF495161 HELIANT-g-TMT_ mRNA     (909)  -------------------------------------------------------------------------------
DQ229828_F1R92                   (3806) CGTC----------------AAGCAAAATGTACCTGTGTTTACGTGGTTCTCACAGTTTTCTAGATTCTGTTTTGAGCA
DQ229829_F1R92                   (3524) CGTCTTTTGCTTTGAGTTTCAAGCAAAATGTACATGTGTTTACGTGGTTCTCACAGTTTTCTAGATTCTGTTTTGAGCA
DQ229830_F1R92                   (3530) CGTCTTTTGCTTTGAGTTTCAAGCAAAATGTACATGTGTTTACGTGGTTCTCACAGTTTTCTAGATTCTGTTTTGAGCA
DQ229831_F1R92                   (3647) CGTCTTTTGCATTGAATTTCAGGCAAAATGTACATGTGTTTACGCGGTTCTCACAGTTTTCTAGATTCTGTTTTGAGCA
DQ229832_F1R92                   (3647) CGTCTTTTGCATTGAATTTCAGGCAAAATGTACATGTGTTTACGCGGTTCTCACAGTTTTCTAGATTCTGTTTTGAGCA
IAST-1_haplotype_4               (3647) CGTCTTTTGCATTGAATTTCAGGCAAAATGTACATGTGTTTACGCGGTTCTCACAGTTTTCTAGATTCTGTTTTGAGCA
nmsT2100_haplotype_4             (3647) CGTCTTTTGCATTGAATTTCAGGCAAAATGTACATGTGTTTACGCGGTTCTCACAGTTTTCTAGATTCTGTTTTGAGCA
DQ229833_F1R92                   (3800) CGTCTTTTGCATTGAATTTCAAGCAAAATGTACATGTGTTTACGTGGTTCTCACAGTTTTCTAGATTCTGGTTTGAGCA
DQ229834_F1R92                   (3800) CGTCTTTTGCATTGAATTTCAAGCAAAATGTACATGTGTTTACGTGGTTCTCACAGTTTTCTAGATTCTGGTTTGAGCA
IAST-1_haplotype_5               (3801) CGTCTTTTGCATTGAATTTCAAGCAAAATGTACATGTGTTTACGTGGTTCTCACAGTTTTCTAGATTCTGGTTTGAGCA
nmsT2100_haplotype_5             (3802) CGTCTTTTGCATTGAATTTCAAGCAAAATGTACATGTGTTTACGTGGTTCTCACAGTTTTCTAGATTCTGGTTTGAGCA
Consensus                        (3872) CGTCTTTTGCATTGAATTTCAAGCAAAATGTACATGTGTTTACGTGGTTCTCACAGTTTTCTAGATTCTGTTTTGAGCA
```

```
                                                                                                  Section 51
                             (3951) 3951     3960      3970      3980      3990      4000      4010              4029
EF495161 HELIANT-g-TMT_ mRNA  (909) ------------------------------------------------------------GTTGGAAGTCCATAAGAGG
        DQ229828_F1R92 (3869) CG-TCCTTTTATATGTAATTTTTTAATTTCCTTATATTGTTAATGGTTAAAATTGATTAGGTTGGAAGTCCATAAGAGG
        DQ229829_F1R92 (3603) CGGTCCTTTTATATGTAATTTTTTAATTTCCTTATATTGTTAATGATTAAAATTGATTAGGTTGGAAGTCCATAAGAGG
        DQ229830_F1R92 (3609) CGGTCCTTTTATATGTAATTTTTTAATTTCCTTATATTGTTAATGATTAAAATTGATTAGGTTGGAAGTCCATAAGAGG
        DQ229831_F1R92 (3726) CG-TCCTTTTATATGTAATTTTTTAATTTCCTTATATTGTTAATGATTAAAATTGATTAGGTTGGAAGTCCATAAGAGG
        DQ229832_F1R92 (3726) CG-TCCTTTTATATGTAATTTTTTAATTTCCTTATATTGTTAATGATTAAAATTGATTAGGTTGGAAGTCCATAAGAGG
    IAST-1_haplotype_4 (3726) CG-TCCTTTTATATGTAATTTTTTAATTTCCTTATATTGTTAATGATTAAAATTGATTAGGTTGGAAGTCCATAAGAGG
  nmsT2100_haplotype_4 (3726) CG-TCCTTTTATATGTAATTTTTTAATTTCCTTATATTGTTAATGATTAAAATTGATTAGGTTGGAAGTCCATAAGAGG
        DQ229833_F1R92 (3879) CG-TCCTTTTATATGTAATTTTTTAATTTCCTTATATTGTTAATGATTAAAATTGATTAGGTTGGAAGTCCATAAGAGG
        DQ229834_F1R92 (3879) CG-TCCTTTTATATGTAATTTTTTAATTTCCTTATATTGTTAATGATTAAAATTGATTAGGTTGGAAGTCCATAAGAGG
    IAST-1_haplotype_5 (3880) CG-TCCTTTTATATGTAATTTTTTAATTTCCTTATATTGTTAATGATTAAAATTGATTAGGTTGGAAGTCCATAAGAGG
  nmsT2100_haplotype_5 (3881) CG-TCCTTTTATATGTAATTTTTTAATTTCCTTATATTGTTAATGATTAAAATTGATTAGGTTGGAAGTCCATAAGAGG
             Consensus (3951) CG TCCTTTTATATGTAATTTTTTAATTTCCTTATATTGTTAATGATTAAAATTGATTAGGTTGGAAGTCCATAAGAGG

                                                                                                  Section 52
                             (4030) 4030      4040      4050      4060      4070      4080      4090              4108
EF495161 HELIANT-g-TMT_ mRNA  (928) GGCAATGGTAATGCCACTAATGATTGAAGGATTTAAGAAGGATGTAATAAAATTCTCCATCATTACATGCAAAAAGCCT
        DQ229828_F1R92 (3947) GGCAATGGTAATGCCACTAATGATTGAAGGATTTAAGAAGGATGTAATTAAATTCTCCATCATTACATGCAAAAAGCCT
        DQ229829_F1R92 (3682) GGCAATGGTAATGCCACTAATGATTGAAGGATTTAAGAAGGATGTAATAAAATTCTCCATCATTACATGCAAAAAGCCT
        DQ229830_F1R92 (3688) GGCAATGGTAATGCCACTAATGATTGAAGGATTTAAGAAGGATGTAATAAAATTCTCCATCATTACATGCAAAAAGCCT
        DQ229831_F1R92 (3804) GGCAATGGTAATGCCACTAATGATTGAAGGATTTAAGAAGGATGTAATAAAATTCTCCATCATTACATGCAAAAAGCCT
        DQ229832_F1R92 (3804) GGCAATGGTAATGCCACTAATGATTGAAGGATTTAAGAAGGATGTAATAAAATTCTCCATCATTACATGCAAAAAGCCT
    IAST-1_haplotype_4 (3804) GGCAATGGTAATGCCACTAATGATTGAAGGATTTAAGAAGGATGTAATAAAATTCTCCATCATTACATGCAAAAAGCCT
  nmsT2100_haplotype_4 (3804) GGCAATGGTAATGCCACTAATGATTGAAGGATTTAAGAAGGATGTAATAAAATTCTCCATCATTACATGCAAAAAGCCT
        DQ229833_F1R92 (3957) GGCAATGGTAATGCCACTAATGATTGAAGGATTTAAGAAGGATGTAATAAAATTCTCCATCATTACATGCAAAAAGCCT
        DQ229834_F1R92 (3957) GGCAATGGTAATGCCACTAATGATTGAAGGATTTAAGAAGGATGTAATAAAATTCTCCATCATTACATGCAAAAAGCCT
    IAST-1_haplotype_5 (3958) GGCAATGGTAATGCCACTAATGATTGAAGGATTTAAGAAGGATGTAATAAAATTCTCCATCATTACATGCAAAAAGCCT
  nmsT2100_haplotype_5 (3959) GGCAATGGTAATGCCACTAATGATTGAAGGATTTAAGAAGGATGTAATAAAATTCTCCATCATTACATGCAAAAAGCCT
             Consensus (4030) GGCAATGGTAATGCCACTAATGATTGAAGGATTTAAGAAGGATGTAATAAAATTCTCCATCATTACATGCAAAAAGCCT
```

```
                                                                                                              Section 53
                           (4109) 4109       4120      4130      4140      4150      4160      4170            4187
EF495161 HELIANT-g-TMT_ mRNA (1007) GAATAAAAATGGATGGAGTCATTCGTATAATCGTATGTATGTATTCTGGAATTATGTCACTGTTTTCCTTTCTTTTATT
              DQ229828_F1R92 (4026) GAATAAAAATGGATGGAGTCATTCGTATAATCGTATGTATGTATTCTGGAATTATGTCACTGTTTTCCTTTCTTTTATT
              DQ229829_F1R92 (3761) GAATAAAAATGGATGGAGTCATTCGTATAATCATATGTATGTATTCTGGAATTATGTCACTGTTTTCCTTTCTTTTATT
              DQ229830_F1R92 (3767) GAATAAAAATGGATGGAGTCATTCGTATAATCATATGTATGTATTCTGGAATTATGTCACTGTTTTCCTTTCTTTTATT
              DQ229831_F1R92 (3883) GAATAAAAATGGATGGAGTCATTCGTATAATCATATGTATGTATTCTAGAATTATGTCACTGTTTTCCTTTCTTTTATT
              DQ229832_F1R92 (3883) GAATAAAAATGGATGGAGTCATTCGTATAATCATATGTATGTATTCTAGAATTATGTCACTGTTTTCCTTTCTTTTATT
          IAST-1_haplotype_4 (3883) GAATAAAAATGGATGGAGTCATTCGTATAATCATATGTATGTATTCTAGAATTATGTCACTGTTTTCCTTTCTTTTATT
         nmsT2100_haplotype_4 (3883) GAATAAAAATGGATGGAGTCATTCGTATAATCATATGTATGTATTCTAGAATTATGTCACTGTTTTCCTTTCTTTTATT
              DQ229833_F1R92 (4036) GAATAAAAATGGATGGAGTCATTCGTATAATCGTATGTATGTATTCTGGAATTATGTCACTGTTTTCCTTTCTTTTATT
              DQ229834_F1R92 (4036) GAATAAAAATGGATGGAGTCATTCGTATAATCGTATGTATGTATTCTGGAATTATGTCACTGTTTTCCTTTCTTTTATT
          IAST-1_haplotype_5 (4037) GAATAAAAATGGATGGAGTCATTCGTATAATCGTATGTATGTATTCTGGAATTATGTCACTGTTTTCCTTTCTTTTATT
         nmsT2100_haplotype_5 (4038) GAATAAAAATGGATGGAGTCATTCGTATAATCGTATGTATGTATTCTGGAATTATGTCACTGTTTTCCTTTCTTTTATT
                   Consensus (4109) GAATAAAAATGGATGGAGTCATTCGTATAATC TATGTATGTATTCTGGAATTATGTCACTGTTTTCCTTTCTTTTATT
                                                                                                              Section 54
                           (4188) 4188        4200      4210      4220      4230      4240      4250            4266
EF495161 HELIANT-g-TMT_ mRNA (1086) TTCGCAAGTCGCCATGTATCTCAA-------------------------------------------------------
              DQ229828_F1R92 (4105) TTCGCAAGTCGCCATGTATCTCTATGTACTATGTTGTGTGCCCTCAAGTTCCGTCGAGTCATAATCCAAGGTTCTACGC
              DQ229829_F1R92 (3840) TTCACAAGTCGCCATATATCTCTATGTACTATGTTGTGTGCCCTCAAGTTCCGTCGAGTCATAATCCAAGGTTCTACGC
              DQ229830_F1R92 (3846) TTCACAAGTCGCCATATATCTCTATGTACTATGTTGTGTGCCCTCAAGTTCCGTCGAGTCATAATCCAAGGTTCTACGC
              DQ229831_F1R92 (3962) TTCGCAAGTCACCATATATCTCTATGTACTATGTTGTGTGCCCTCAAGTTCCGTCGAGTCATAATCCAAGGTTCTACCG
              DQ229832_F1R92 (3962) TTCGCAAGTCACCATATATCTCTATGTACTATGTTGTGTGCCCTCAAGTTCCGTCGAGTCATAATCCAAGGTTCTACCG
          IAST-1_haplotype_4 (3962) TTCGCAAGTCACCATATATCTCTATGTACTATGTTGTGTGCCCTCAAGTTCCGTCGAGTCATAATCCAAGGTTCTACCG
         nmsT2100_haplotype_4 (3962) TTCGCAAGTCACCATATATCTCTATGTACTATGTTGTGTGCCCTCAAGTTCCGTCGAGTCATAATCCAAGGTTCTACCG
              DQ229833_F1R92 (4115) TTCGCAAGTCGCCATGTATCTCTATGTACTATGTTGTGTGCCCTCAAGTTCCGTCGAGTCATAATCCAAGGTTCTACGC
              DQ229834_F1R92 (4115) TTCGCAAGTCGCCATGTATCTCTATGTACTATGTTGTGTGCCCTCAAGTTCCGTCGAGTCATAATCCAAGGTTCTACGC
          IAST-1_haplotype_5 (4116) TTCGCAAGTCGCCATGTATCTCTATGTACTATGTTGTGTGCCCTCAAGTTCCGTCGAGTCATAATCCAAGGTTCTACGC
         nmsT2100_haplotype_5 (4117) TTCGCAAGTCGCCATGTATCTCTATGTACTATGTTGTGTGCCCTCAAGTTCCGTCGAGTCATAATCCAAGGTTCTACGC
                   Consensus (4188) TTCGCAAGTCGCCAT TATCTCTATGTACTATGTTGTGTGCCCTCAAGTTCCGTCGAGTCATAATCCAAGGTTCTACGC
```

Section 55

```
                          (4267)  4267         4280      4290      4300      4310      4320      4330          4345
EF495161 HELIANT-g-TMT_ mRNA (1110)  -------------------------------------------------------------------------------
         DQ229828_F1R92 (4184)  GTATTGATTATGCCGGGACCATCTAGTGGTGATGCATAAGAATCATGAATGTGTACTATTATAGACAATGGCATCCCAA
         DQ229829_F1R92 (3919)  GTATTGATTATGCCGGGACCATCTAGTGGTGATGCATAAGAATCATGAATGTGTACTATTATAGACAATGGCATCCCAA
         DQ229830_F1R92 (3925)  GTATTGATTATGCCGGGACCATCTAGTGGTGATGCATAAGAATCATGAATGTGTACTATTATAGACAATGGCATCCCAA
         DQ229831_F1R92 (4041)  GCATTGATTATACCGGGACCATCTAGTGGTGATGCATAAGAATCATGAATGTGTACTATTATAGACAATGGCATCCCAA
         DQ229832_F1R92 (4041)  GCATTGATTATACCGGGACCATCTAGTGGTGATGCATAAGAATCATGAATGTGTACTATTATAGACAATGGCATCCCAA
     IAST-1_haplotype_4 (4041)  GCATTGATTATACCGGGACCATCTAGTGGTGATGCATAAGAATCATGAATGTGTACTATTATAGACAATGGCATCCCAA
   nmsT2100_haplotype_4 (4041)  GCATTGATTATACCGGGACCATCTAGTGGTGATGCATAAGAATCATGAATGTGTACTATTATAGACAATGGCATCCCAA
         DQ229833_F1R92 (4194)  GTATTGATTATGCCGGGACCATCTAGTGGTGATGCATAAGAATCATGAATGTGTACTATTATGGACAATGGCATCCCAA
         DQ229834_F1R92 (4194)  GTATTGATTATGCCGGGACCATCTAGTGGTGATGCATAAGAATCATGAATGTGTACTATTATGGACAATGGCATCCCAA
     IAST-1_haplotype_5 (4195)  GTATTGATTATGCCGGGACCATCTAGTGGTGATGCATAAGAATCATGAATGTGTACTATTATGGACAATGGCATCCCAA
   nmsT2100_haplotype_5 (4196)  GTATTGATTATGCCGGGACCATCTAGTGGTGATGCATAAGAATCATGAATGTGTACTATTATGGACAATGGCATCCCAA
              Consensus (4267)  GTATTGATTATGCCGGGACCATCTAGTGGTGATGCATAAGAATCATGAATGTGTACTATTATAGACAATGGCATCCCAA
```

Section 56

```
                          (4346)  4346   4352
EF495161 HELIANT-g-TMT_ mRNA (1110)  -------
         DQ229828_F1R92 (4263)  GGAATTA
         DQ229829_F1R92 (3998)  GGAATTA
         DQ229830_F1R92 (4004)  GGAATTA
         DQ229831_F1R92 (4120)  GGAATTA
         DQ229832_F1R92 (4120)  GGAATTA
     IAST-1_haplotype_4 (4120)  GGAATTA
   nmsT2100_haplotype_4 (4120)  GGAATTA
         DQ229833_F1R92 (4273)  GGAATTA
         DQ229834_F1R92 (4273)  GGAATTA
     IAST-1_haplotype_5 (4274)  GGAATTA
   nmsT2100_haplotype_5 (4275)  GGAATTA
              Consensus (4346)  GGAATTA
```
